# Current Knowledge of the Antidepressant Activity of Chemical Compounds from *Crocus sativus* L.

**DOI:** 10.3390/ph16010058

**Published:** 2022-12-30

**Authors:** Renata Matraszek-Gawron, Mirosława Chwil, Karol Terlecki, Michał Marian Skoczylas

**Affiliations:** 1 Department of Botany and Plant Physiology, University of Life Sciences in Lublin, Akademicka 15 Street, 20-950 Lublin, Poland; 2 Department of Vascular Surgery and Angiology, Medical University of Lublin, Racławickie 1 Street, 20-059 Lublin, Poland; 3 Department of Diagnostic Imaging and Interventional Radiology, Pomeranian Medical University in Szczecin, Unii Lubelskiej 1 Street, 71-252 Szczecin, Poland

**Keywords:** anxiety, crocin and crocetin, depression, Iridaceae, neuroprogression and neurotransmitters, adjuvant phytotherapy, safety of saffron treatment, saffron and safranal

## Abstract

Psychotropic effect of *Crocus sativus* L. (family Iridaceae) biologically active chemical compounds are quite well documented and they can therefore be used in addition to the conventional pharmacological treatment of depression. This systematic review on antidepressant compounds in saffron crocus and their mechanisms of action and side effects is based on publications released between 1995–2022 and data indexed in 15 databases under the following search terms: antidepressant effect, central nervous system, *Crocus sativus*, cognitive impairement, crocin, crocetin, depression, dopamine, dopaminergic and serotonergic systems, picrocrocin, phytotherapy, neurotransmitters, safranal, saffron, serotonin, and biologically active compounds. The comparative analysis of the publications was based on 414 original research papers. The investigated literature indicates the effectiveness and safety of aqueous and alcoholic extracts and biologically active chemical compounds (alkaloids, anthocyanins, carotenoids, flavonoid, phenolic, saponins, and terpenoids) isolated from various organs (corms, leaves, flower petal, and stigmas) in adjuvant treatment of depression and anxiety. Monoamine reuptake inhibition, *N*-methyl-d-aspartate (NMDA) receptor antagonism, and gamma-aminobutyric acid (GABA)-α agonism are the main proposed mechanism of the antidepressant action. The antidepressant and neuroprotective effect of extract components is associated with their anti-inflammatory and antioxidant activity. The mechanism of their action, interactions with conventional drugs and other herbal preparations and the safety of use are not fully understood; therefore, further detailed research in this field is necessary. The presented results regarding the application of *C. sativus* in phytotherapy are promising in terms of the use of herbal preparations to support the treatment of depression. This is particularly important given the steady increase in the incidence of this disease worldwide and social effects.

## 1. Introduction

### 1.1. Symptoms of Depression

Major depressive disorder (MDD), known also as depression (lat. *depressio* ‘deepness’ from *deprimere* ‘overwhelm’), is a chronic, recurrent, and potentially life-threatening mental disorder characterised by at least two weeks of omnipresent low mood. It is usually accompanied with persistent feeling of sadness, anhedonia, pain without a clear cause, difficulties in thinking and concentration, loss of interest in doing anything, psychomotor retardation, fatigue, spending time sleeping, feelings of worthlessness or inappropriate guilt, and recurrent thoughts of death. These symptoms cause distress or impairment in social life and are not an effect of the influence of other medical conditions [1,2,3,4,5,6,7,8]. The spectrum of symptoms in individual patients depends on the type of depression and ranges from excessive consumption of chocolate during episodes of seasonal affective disorder to nihilistic delusions of extensive and absurd content characteristic of Cotard syndrome, which is a rare mental disorder in which the affected person holds the delusional belief that they are dead, do not exist, are putrefying, or have lost their blood or internal organs [9,10]. Depression can affect people at any age (children, adolescents, adults and old individuals) and is characterised by high mortality rates throughout one’s lifetime. Depression very often affects women during the menopausal transition, pregnant women, and both parents after childbirth. “Secondary depression” may be a result of a chronic or terminal medical condition, such as asthma, Lyme disease, cancer, COVID-19, or HIV/AIDS [11,12,13,14,15,16,17,18,19,20,21,22].

### 1.2. Pathogenesis of Depression

The major depressive disorder has a neuroprogressive nature [23,24] with accelerated cellular aging [25,26,27,28,29], and a higher risk of co-morbid somatic age-related diseases [25,26,27,28,29,30,31,32,33]. Neuroprogression recognised at the clinical, structural, and biochemical levels in the major depressive disorder includes stage-related neurodegeneration, cell death, reduced neurogenesis, reduced neuronal plasticity, and increased autoimmune responses [33,34,35,36,37,38,39]. Depression in its endogenous form accompanies many organic diseases (including infectious diseases), as well as being related to disease processes or treatment (e.g., pharmacologically induced immune-related depression). It can also be a result of stressful events, chronic lifestyle diseases, pollution (e.g., cadmium), and a reduced ability to adapt to the environment or cultural accommodation [40,41,42,43,44,45,46,47,48,49].

The theories of depression pathophysiology are mainly based on: (i) the monoaminergic hypothesis which indicates insufficient activity of monoamine neurotransmitters, (ii) abnormalities analysed in the limbic cortical model and cortico-striatal model, (iii) hypothalamic–pituitary–adrenal axis dysfunction, (iv) overactivation of proinflammatory cytokines [50,51,52,53,54,55,56,57,58].

Results of genetic and neuroradiological studies suggest that changes in specific genes influencing some parts of the brain (e.g., prefrontal brain regions, hippocampus and white matter tracts) may cause major depressive disorder. Many genes related to this disease have been found and epigenetic factor analyses contribute to a deepening of this research [59,60,61,62,63]. According to Wray et al. [64] all humans carry lesser or greater numbers of genetic risk factors for major depression. It should be added that genetic relationships between depression and other diseases, including Crohn’s disease, are also still studied [62].

### 1.3. Economic and Social Cost of Depression

Depression is one of the most common and still increasing global multidimensional mental health problems, affecting all areas of human life, with high economic and social costs. In 2017, major depressive disorder affected approximately 163 million people (2% of the global population). Now it is estimated that 40 million people suffer from depression across Europe and over 260 million people worldwide. By 2030, depression is supposed to be the leading cause of disease burden in high-income countries. The total direct healthcare cost of depression, depending on the jurisdiction where the analysis was run and the range of cost items included, ranges between €508 and €24,069, whilst indirect costs range between €1963 and €27,364. The economic impact of depression in the European Economic Area (EEA) is thought to be up to €92 billion annually. Decreased productivity is linked to unemployment, poor housing and poverty and therefore many are trapped in a circle of deprivation and illness [65,66,67].

## 2. Phytotherapy for Depression

In addition to psychotherapy and electroconvulsive therapy, pharmacotherapy is one of the methods for treatment of depression. Currently, increasing attention is being paid to the application of phytochemicals and their derivatives as preventive and therapeutic compounds in supportive therapy of patients treated for neuropsychiatric diseases, including neurodegenerative disorders and depression. 

### 2.1. Taxonomic Diversity of Plants Used in Depression Therapy

Given the numerous undesirable effects of antidepressants and electroconvulsive therapy, effective and safer therapeutic options are being explored [68,69]. It is reasonable to draw attention to the potential of the application of drugs based on phytochemicals with lower toxicity and effective action [70,71,72]. Currently, phytotherapy supporting the treatment of depression and alleviating its symptoms is based on various active chemical compounds obtained from many plant taxa from different families of monocotyledons: *Cyperaceae* [73], *Iridaceae* [71,74,75], *Xanthorrhoeaceae* [74,76] as well as dicotyledons, e.g., *Apiaceae* [77], *Aquifoliaceae* [78,79], *Asteraceae* [80], *Capparaceae* [81,82], *Caprifoliaceae* [83,84], *Fabaceae* [85], *Hypericaceae* [86,87,88,89,90,91], *Lamiaceae* [92,93,94], *Lauraceae* [95], *Passifloraceae* [96,97,98], *Polygalaceae* [99], *Rutaceae* [100,101], *Thymelaeaceae* [73], and *Solanaceae* [102] (Table 1).

The biologically active chemical compounds present in these plants have antidepressant activity comparable to that of standard anxiolytics and antidepressants [81,103,104,105,106]. *Crocus sativus* from the Iridaceae family is one of many such plant species. The rationale behind the choice of this plant is not only its well-known medicinal properties and wide use in folk medicine to alleviate symptoms of many diseases, but also its medicinal applications. Especially in the pandemic times, a new search for safe phytochemicals from *Crocus sativus* with antidepressant effects is the focus of clinical trials. Hence, this species was analysed in detail with reference to the current phytotherapeutic and clinical knowledge.

### 2.2. Crocus sativus 

#### 2.2.1. *C. sativus*—Characteristics of Pharmacopoeial Raw Material

*Crocus sativus* L. (family Iridaceae), commonly known as saffron crocus, is a therapeutic plant native to Asia Minor and Southern Europe [133,134,135,136,137,138,139]. The plant is cultivated in Iran, India, Afghanistan, Greece, Morocco, and Italy [133,134,135,136,137,138,139,140,141,142]. It propagates vegetatively. The plant produces an underground tuber and basal, stiff, lanceolate leaves. Its lilac–purple flowers are composed of six tepals, three stamens, and a pistil with a long style and a tripartite dark orange stigma [143,144,145,146,147]. Stigmas, commonly referred to as saffron, are hand-picked during the flowering period and dried immediately after harvesting. Approximately 110,000 to 200,000 flowers are needed to collect 1 kg of stigmas [148,149,150]. *Croci sativi* stigmas (*Stigma Croci*) are a pharmacopoeial raw material [151,152,153]. They have high economic importance and are the most expensive raw material in the world. Currently, saffron retail prices reach up to $11,000 per kilogram, while the petals are much cheaper [142,143,144,145,146,147,148,149,150,151,152,153,154,155,156,157,158,159,160,161].

#### 2.2.2. Biologically Active Chemical Compounds in Various *C. sativus* Organs

Dried *C. sativus* stigmas contain over 150 volatile compounds, mainly terpenes and their esters [162,163,164,165,166]. Detailed information about the *C. sativus* biologically active compounds and their pharmacological activities was compiled by Mykhailenko et al. [167]. Various organs of *C. sativus*, i.e., the corm, leaf, tepal, stigma, and whole flowers, contain bioactive compounds representing different classes, e.g., anthocyanins, carotenoids, phenolic compounds, flavonoids, carotenoids, saponins, and terpenoids (Table 2).

#### 2.2.3. Application of *C. sativus* in Herbal Medicine and Industry and Therapeutic Activity

*Crocus sativus* is used in Asian and, in particular, Indian (Ayurveda) and Persian (Islamic) traditional medicine (ITM) as a sedative agent to strengthen the body against such stresses as trauma and anxiety, an anticonvulsant and memory enhancer, and a remedy for alleviation of chronic fatigue, depression, and inflammation [71,135]. This therapeutic activity of *Crocus*, known since the 6th century BC, has been confirmed in the most recent basic research conducted on animals (rodents) and in human clinical studies [134,175,176,177,178,179,180,181,182,183].

Currently, there is a search for new methods of treatment based on the use of phytochemicals contained in herbal raw materials with significant efficacy in relieving the symptoms of depression confirmed by meta-analyses and clinical trials [75,90,111]. The numerous side effects of antidepressants as well as the attitudes of many patients preferring herbal rather than conventional drugs support the assessment of the impact of saffron crocus stigmas on depression patients [70,71].

Bioactive compounds of *C. sativus* have a wide range of applications due to their valuable health-enhancing properties [184,185,186]. They are used in many branches of industry, including the pharmaceutical [187,188,189,190,191,192] cosmetic [193,194,195] dairy [196,197], and food [198,199,200,201], industries. These phytochemicals are also used in in the production of nutraceuticals [201,202,203,204,205] and in nanotechnology [206,207,208,209,210], e.g., nanomedicine [211,212] and nanocosmetics [213]. Furthermore, they are applied in therapeutic practice [163,214,215], adjuvant therapy [216,217], and chemopreventive treatment [218,219,220] and have great importance in cosmetic marketing [221], genetic studies, and transgenic plant production [222,223,224,225].

Currently, numerous experiments, cell line studies conducted in various biological models, and clinical trials are ongoing in an attempt to assess the pharmacological effectiveness of biologically active chemical compounds from various organs of saffron crocus in the treatment of some diseases (Table 3, Table 4 and Table 5). These compounds exert a wide spectrum of important healing effects, including antidepressant [175,226,227,228,229], anxiolytic [230,231,232,233,234], and anti-inflammatory [189,204,235,236,237,238,239,240,241,242], activities. Biologically active chemical compounds of saffron crocus have also been shown to have a few other kinds of activity resulting in antimicrobial [243], anticancer [244,245,246,247,248,249,250,251,252], analgesic [176,253,254,255], anticonvulsant [256,257], antitussive [258], antigenotoxic and anticytotoxic [245,259,260,261], relaxant [262,263], antihypertensive [264,265], and antioxidative [171,266,267,268,269,270,271,272,273,274] effects. 

In vitro studies have confirmed the antigenotoxic and anticytotoxic effects of active substances isolated from *C. sativus* [245]. This should be emphasised, as other aspects of the pleiotropic activity of some cytokines and a wide spectrum of the impact of the transcription factor called the nuclear factor kappa-light-chain-enhancer of activated B cells (NF-κB) is present in almost all animal type cells [275,276,277,278].

As reported by Wang et al. [226], the antidepressant properties of stigma aqueous extracts are related to the presence of crocin 1, but further studies regarding the precise site and mechanism of the anti-depressive action of chemical compounds isolated from petroleum ether and dichloromethane fractions of *C. sativus* corms are required. Karimi et al. [175] have found that flavonoids and anthocyanins are the main constituents involved in the antidepressant action of *C. sativus* extracts.

Considering the multidirectional phytotherapeutic effect of *C. sativus*, this paper is a review of available literature data and presents the current information about the effectiveness of bioactive chemical compounds contained in this species and the mechanisms of their action in the supportive therapy of depression, with emphasis on the safety of application of these substances. The thesis of the antidepressant effectiveness was verified by an analysis of the results of the latest basic research conducted in animal models, and human clinical trials. Additionally, the study highlights the difficulties and limitations in laboratory analyses and clinical studies of the antidepressant effects of the phytochemicals and indicates further perspectives of research on their use and potential methods for control of treatment in relation to the disease pathogenesis.

Since depression is a serious growing global health problem with social and economic consequences, intensified investigations are being carried out to search for biologically active chemical compounds of plant origin, which will prove effective in supporting the treatment of this disease. *Crocus sativus* L. is a species known for its healing properties and widely used in folk medicine to alleviate the symptoms of many diseases. The thesis on antidepressant effectiveness was verified by analysis of the results of the latest basic research in cell cultures, animal models and human clinical trials.

**Table 3:** Therapeutic effects of selected biologically active chemical compounds from the classes of anthocyanins, terpenoids, and saponins extracted from *C. sativus* corms and flowers.

Classes of Biologically Active Chemical Compounds	Biologically Active Chemical Compounds	Organ	Therapeutic Effects	Reference
Anthocyanins	delphinidin,	tepals	antioxidant	[279,280]
	malvidin			
	petunidin,			
	3,5-di-O-β-glucosides			
Terpenoids	monoterpenoids	corm	antibacterial anticancer	[174]
	sesquiterpenoids			
Saponins	oleanane type:azafrine 1 azafrine 2		antitumor, increase immune responses to protein-based vaccines	[281]
	bidesmosidic type			
	3-O-d-glucopyranosiduronic acid echinocystic acid 28-O-d-galactopyranosyl-(1→2)-l-arabinopyranosyl-(1→2)-[-dxylopyranosyl-(1→4)]-d-rhamnopyranosyl-(1→2)-[4-O-di-L-rhamnopyranosyl-3,16-dihydroxy-10-oxo-hexadecanoyl]-d-fucopyranoside		antitumor against HeLa cells	[282]

**Table 4:** Therapeutic effects of selected *C. sativus* biologically active chemical compounds from the class of phenolic compounds and essential oils.

Classes of Biologically Active Chemical Compounds		Biologically Active Chemical Compounds	Organ	Therapeutic Effects	Reference
Polyphenol		pyrogallol	stigma	antioxidant	[171]
Phenolic acid	benzoic acid derivatives	gallic acid			
		p-hydroxybenzoic acid	corm		[283]
		salicylic acid			
		gentisic acid			
		syringic acid			
	cinnamic acid derivatives	caffeic acid			
		p-coumaric acid			
		t-ferulic acid			
		cinnamic acid			
Polyphenols		catechol			
Phenolic aldehyde		vanillin			
Essential oils		β-isophorone	stigma	has not been presented	[284]
		β-Linalool			
		α-Isophorone			
		palmitic acid methyl ester			
		α, β-dihydro-β-ionone

**Table 5:** Therapeutic effects of selected *C. sativus* biologically active chemical compounds from the classes of carotenoids extracted from stigmas.

Carotenoids	Therapeutic Effects	Reference
Crocin	inhibited xylene-induced swelling of mouse ear and increased capillary permeability and writhing induced by acetic acid in mice; at 50 mg/kg, it inhibited carrageenan- and fresh egg white-induced oedema of the hind paw in rats. It inhibited sheep red blood cells (SRBC)-induced footpad reaction and inhibited picryl chloride-induced contact dermatitis	[285]
	cytotoxic effect on human and animal adenocarcinoma cells (HT-29 and DHD/K12-PROb cells)	[286]
	a prolonged blood coagulation time in mice and markedly inhibited dose-dependent thrombin- and ADP-induced blood platelet aggregation in rabbits (in vivo); an inhibitory effect on thrombus formation in rats with arteriovenous shunt and relieved respiratory distress due to pulmonary thrombosis in mice induced by ADP and AA	[287]
	cardiovascular protective effects; the cardioprotective effects of crocin may be attributed to the attenuation of [Ca^2+^] through inhibition of ICa-L in rat cardiomyocytes as well as negative inotropic effects on myocardial contractility	[288]
	it affected tubulin polymerisation and structure, increased the microtubule nucleation rate, induced conformational changes in tubulin, and affected several cell processes through interaction with tubulin proteins or microtubules	[289]
Crocetin	vasomodulatory effects in hypertension, improvement of endothelium-dependent acetylcholine relaxations via endothelial nitric oxide, improvement of acetylcholine-induced vascular relaxation in hypertension	[290]
Crocetin	interaction of carotenoids with topoisomerase II, an enzyme involved in cellular DNA–protein interaction, immunomodulatory activity on T Helper Cell Type 1 (Th)1 and Th2, anticancer properties	[219]
Crocin		
Carotene	source of vitamin A, preventive agents against cancer and heart disease, antioxidant and memory effect enhancer	[291]
Crocetin		
Licopene		
β-zeaxanthin		

## 3. Methodology 

This review is a presentation of possible treatment methods available across the range of herbal medicines that are relevant to the pathogenesis of depression, with the indication of ways of treatment control with clinical tests used by authors of the cited papers and medical imaging of brain functions for the future scientific purposes. This publication is based on a search in scientific databases of literature reports covering the contemporary research on antidepressant bioactive substances from *Crocus sativus* L.

### 3.1. Bibliographic Databases and Searched Phrases 

The original scientific publications were found in 15 multidisciplinary specialised scientific databases: Web of Knowledge, EBSCO, Google Scholar, ISI Web of Science, Medline, ProQuest Central, ProQuest SciTech Collection, PubMed, ScienceDirect, Scopus, Springer, Taylor & Francis, Web of Knowledge, Web of Science, and Wiley Online Library. The search engines of these databases provided access to original scientific publications mainly in the fields of medical, preclinical, biological, chemical, and social sciences and sociology. The search was performed using the following phrases: antidepressant effect, central nervous system, *Crocus sativus*, crocin, crocetin, depression, dopamine, dopaminergic and serotonergic systems, picrocrocin, phytotherapy, melatonin, neurotransmitters, safranal, saffron, serotonin, and biologically active compounds, safety of saffron treatment, and saffron in depression add-on/adjuvant therapy.

### 3.2. Number and Methods of Analysis of Resources 

In total, 414 thematically coherent scientific reports (cited in this review) were selected, including 408 original publications and 7 other sources, e.g., chapters from monographs and books. The analysis was focused on original scientific publications on *C. sativus* addressing the following issues: (i) biological activity of chemical compounds in various organs, (ii) therapeutic activity, (iii) antidepressant effect of extracts and their components, (iv) mechanisms of antidepressant action, (v) possible future ways for the therapy and its control to proceed in practice, and (vi) challenges for further research. The results of the studies were arranged and presented in the tables according to scheme: (a) animal studies, and (b) human trials.

## 4. Antidepressant Activity of *C. sativus*

### 4.1. Biologically Active Chemical Compounds with Antidepressant Effects

**Figure 1:**
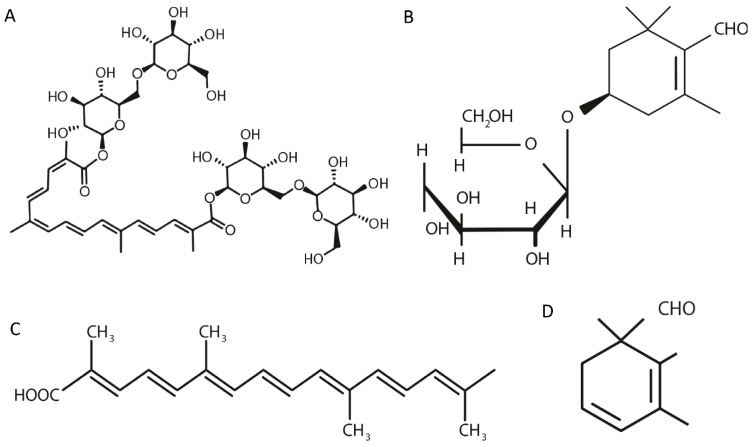
Structural formula of biologically active chemical compounds: crocin (**A**), crocetin (**B**), picrocrocin (**C**), and safranal (**D**) present in *C. sativus* [298,299,300,301].

Among the biologically active chemical compounds identified in various *C. sativus* organs, the antidepressant effects have mainly been ascribed to safranal, crocin, crocetin, and picrocrocin [216,292,293,294,295,296]. The structural formulas of these phytochemicals are shown in Figure 1. The contents of picrocrocin, crocin, crocetin gentiobiose glucose ester and crocetin di-glucose ester in ethanol extracts of *Crocus sativus* L. are about 40, 20, 10 and 2–3%, respectively [297].

The content of safranal in saffron crocus stigmas is in the range of 0.1–0.6% d.w. [302,303,304], however, other authors have reported that the content of this compound ranges from 1.07 to 6.15% d.w. [305]. In turn, there are also reports showing that the content of safranal in red stigma samples were 49.64 and 50.29%, while in threads with yellow styles it was 50.42%, 57.02% and 61.31% [306]. The concentration of crocin was estimated at 20–30% of the total dry matter of the spice [154,303,307], but some study results revealed a much wider range of this compound, i.e., 0.85–32.4% [305]. As reported by Zhang and co-workers [308], the content of crocin varied significantly among saffron populations from seven different production areas, i.e., Nepal, Greece, Morocco, Spain, Iran, and China (Jiande, Chongming), and ranged from 80.59 to 230.36 mg/g. Zeka et al. [309] reported that dried petals contained 0.6% of crocin. As suggested by Acar et al. [302] the crocin content of commercial saffron dried in a freeze dryer and dried naturally under the sun was 900 and 600 mg/g, respectively. Azarabadi and Özdemir [306] found that crocin amount was higher in red stigmas samples (66.67 mg/g) than in yellow stigmas samples (51.66 mg/g). The content of crocetin esters represents 16–30% of saffron stigma [310] Crocin is largely absent from petal extracts [311].

The picrocrocin content found in dried stigmas ranged from 0.8 to 26.6% [312,313]. Some authors propose a slightly narrower range of limits for the content of this compound i.e., 0.79–12.94% [305,314], 7–20% [315] and 5–7 mg/g d.w. [303]. The reasons for such a large discrepancy of limits in the content of safranal, crocin, crocin, crocetin and picrocorocin should be sought for in the different drying methods, and storage and extraction conditions of saffron, which degrade these compounds significantly; the degree of degradation depends on temperature, humidity, light irradiation and other compounds in the environment [305].

Othman and co-workers [316] found markedly various crocin and crocetin content in saffron crocus stigmas from different geographical origins. Iranian saffron was characterised by substantially higher amount of crocin content than Turkish and Kashmiri saffron (11,414.67 and 311.63 µg/g d.m. of crocin, respectively). In turn crocetin was detectable in Iranian and Turkish (1054.73 and 186.64 µg/g d.m. of crocetin, respectively) but not in Kashmiri saffron. These differences were suggested to be related to various environmental factors, e.g., climatic conditions, agricultural practices, and stigma separation, as well as storing and drying processes [316].

The information about physicochemical properties of the saffron crocus bioactive compounds, which are important in the preparation of medicinal formulations were presented in the Table 6. The most important stigma constituents include antioxidative carotenoids (with the water-soluble crocin and its derivatives responsible for the colour: zeaxanthin β-carotenes, lycopene), anthocyanins (delphinidin), terpenes (fat-soluble safranal responsible for the odour and aroma and its monoterpene glycoside precursor picrocrocin responsible for the special bitter flavour), polysaccharides, amino acids, proteins, starch, mineral matter, gums, and other chemical compounds [191,201,214,228,317,318].

α-Crocin (systematic IUPAC name: 8, 8-diapo-8, 8-carotenoic acid), which is primarily responsible for the golden yellow-orange colour of the stigma, is a *trans*-crocetin di-(β-d-) ester. Crocin, underlying the aroma of saffron, is a digentiobiose ester of crocetin. Crocins are hydrophilic carotenoids that are either monoglycosyl or di-glycosyl polyene esters of crocetin. In contrast, crocetin is a hydrophobic and thus oil-soluble conjugated polyene dicarboxylic acid. However, the product of esterification of crocetin with two water-soluble gentiobioses (sugars) is soluble in water [300,317,319].

It is believed that the glycolysed carotenoid crocetin—a natural apocarotenoid dicarboxylic acid—is the most pharmacologically active constituent of stigma extracts, besides the carboxylic carotenoid crocin. Saffron extracts and crocetin had a clear binding capacity at the phencyclidine (PCP) binding side of the *N*-methyl-d-aspartate receptor (NMDA receptor; NMDAR) and at the σ_1_ (sigma-1) receptor, while the crocins and picrocrocin were not effective, which give the biochemical support for the pharmacological effect of saffron including depression treatment [188,191,294,295,296,320].

**Figure 2:**
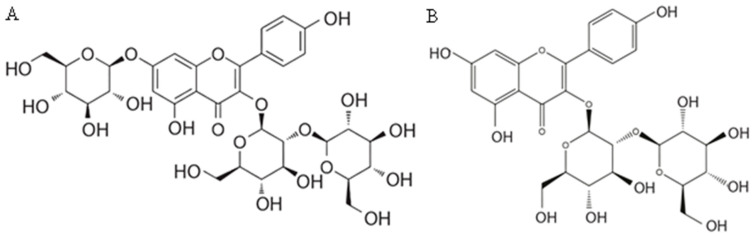
Structural formula of kaempferol 3-*O*-*β*-sophoroside-7-*O*-*β*-glucoside (**A**) and kaempferol 3-*O*-*β*-sophoroside (**B**) from *C. sativus* [166].

Hosseinzadeh et al. [321] postulated that the flavonol kaempferol was responsible for the antidepressant effect of *C. sativus* petals. Kaempferol 3-*O*-*β*-sophoroside-7-*O*-*β*-glucoside is the most important flavonol in saffron. Its relative content ranges from 37% to 63% of total flavonoids, and its absolute content values vary between 1.47 and 2.58 equivalent milligrams of rutin g^−1^. Kaempferol 3-*O*-*β*-sophoroside is the next major flavonol in order of importance, related to the concentration in saffon. Its relative content ranges from 16% to 47% of total flavonoids with absolute content values ranging from 0.61 to 3.12 equivalent milligrams of rutin g^−1^ [166]. This flavonol was extracted from saffron floral bioresidues that were mainly made up of tepals, and an extract yield of 2.3 mg g^−1^ dry weight was obtained. Its content in tepals ranges from 0.69 to 12.60 equivalent milligrams of kaempferol 3-*O*-*β*-glucoside g^1^ dry weight [169,309]. According to other literature data, the content of kaempferol-3-*O*-sophoroside in saffron crocus tepals was 62.19–99.48 mg/g [322]. Another flavonol found in saffron is kaempferol 3,7,4′-tri-*O*-*β*-glucoside. Its relative content ranges from 16% to 22% of total flavonoids, and its absolute content values ranges from 0.59 to 1.09 equivalent milligrams of rutin g^−1^ [166]. In the stamen, the number of flavonoids was lower than in the tepal. The amount of kaempferol-3-*O*-glucoside, as the most abundant compound, ranged between 1.72–7.44 mg/g [322]. Structures of saffron crocus kaempferol 3-*O*-*β*-sophoroside-7-*O*-*β*-glucoside and kaempferol 3-*O*-*β*-sophoroside are presented in Figure 2.

**Table 6:** General characteristics of some biologically active chemical compounds of *C. sativus* showing antidepressant action.

Biologically Active Chemical Compounds		Chemical Formula	Molecular Weight [g/mol]	Physical Description	Melting Point [°C]	Solubility	Reference
Traditional Name	Classes						
Safranal	monoterpene aldehyde	C_10_H_14_O	150.22	pale yellowish oily liquid, tobacco-herbaceous odour	<25	insoluble in water, soluble in oils, miscible in ethanol	[293,323,324,325,326]
Crocin	diterpenoid	C_26_H_34_O_9_ * C_32_H_44_O_14_ ** C_32_H_44_O_14_ *** C_44_H_64_O_24_^†^ C_50_H_24_O_2_^††^	976.96	solid	186	freely soluble in hot water, sparingly soluble in alcohol, ether and other organic solvents	[251,325,327,328,329,330,331]
Crocetin	tetraterpenoid	C_20_H_24_O_4_	328.40	reddish crystals	186	slightly soluble in aqueous solution, soluble in organic bases	[331]
Picrocrocin	monoterpene glycoside	C_16_H_26_O_7_	330.37	ns	164–156	Soluble in water	[251,305,325]

Explanations: no—not specified, * crocin-1, ** crocin-2, *** crocin-3, ^†^ crocin-4, ^††^ crocin-5.

### 4.2. Antidepressant Effect of C. sativus L.

Extracts of *C. sativus* and their active biologically chemical substances have been shown to exert beneficial effects on the activity of the central nervous system. Therefore, they can potentially be used as adjuvant agents in treatment of mental disorders, including depression [204,233,311,332,333,334,335,336]. Literature data have demonstrated in a number of in vitro, in vivo, basic and clinical trials that dried *C. sativus* stigmas and petals as well as their active ingredients exhibit strong antidepressant properties similar to those of the current conventional antidepressant medications from the class of the selective serotonin re-uptake inhibitors (SSRIs), including citalopram [337], fluoxetine (Prozac) [338,339,340,341,342], and sertraline [343], as well as the tricyclic antidepressant imipramine [176,344] and the benzodiazepine diazepam [345,346]. Table 7, Table 8, Table 9 and Table 10 summarise the results of preclinical studies, conducted in animal models and human clinical trials, on the antidepressant effect of extracts and bioactive chemical compounds from the saffron crocus.

Although the results of clinical trials clearly suggest that saffron reduces the severity of depression based on Hamilton Depression Rating Scale (HAM-D) and Beck’s Depression Inventory (BDI) scores, the optimum dose and duration of treatment is still unclear [75].

Saffron and its bioactive constituents (crocetin esters, picrocrocin, and safranal) may be considered as a potential adjuvant in the form of anti-depressants in the future drug formulations. Recently, they seem to be a suitable candidate for the management of anxiety, depression, neuropsychiatric disorders and the other long-term effects including subacute and chronic abnormalities of severe acute respiratory syndrome coronavirus 2 (SARS-CoV-2) infection such as fatigue, dyspnoea, cognitive problems, sleep abnormalities, and deterioration in the quality of life. Detailed research on dosage, methods of administration and others needs to be undertaken to explore the potential of saffron in managing the health issues arising due to the COVID-19 pandemic [397,398]. Moreover, crocin appears to reduce the COVID-19-related cytokine cascade and downregulate angiotensin-converting enzyme 2 (ACE2) gene expression. Lastly, in silico studies suggest that saffron’s astragalin and crocin could have inhibitory actions on the SARS-CoV-2 protease and spike protein, respectively. However, future appropriate randomised clinical trials using biomarkers as surrogates to assess inflammatory status should be designed in order to assess the clinical efficacy of saffron and allow its use as an adjunct treatment modality, particularly in resource-poor settings where access to drugs may be limited [399]. Soheilipur and co-workers claim [400] that the oral use of a single-dose of 40 mg saffron extract is effective in alleviating anxiety among the candidates for coronary angiography (CA), while lippia extract (capsule 40 mg; *Lippia citriodora* Kunth) and saffron–lippia (20 mg:20 mg) extract combination had no significant effects on their anxiety. 

Saffron application is recognised as a promising natural and safe nutritional strategy to improve sleep duration and quality. The investigations carried out by Shahdadi et al. [401] revealed that daily (between 12 noon and 2 pm) intake of a 300 mg saffron capsule after lunch for a week was effective in reducing anxiety and improving the quality of sleep among diabetic patients. Six weeks of saffron extract supplementation (15 mg/day) to the subjects presenting with mild-to-moderate sleep disorders associated with anxiety led to an increased time in bed assessed by actigraphy, to an improved ease of getting to sleep as evaluated by the LSEQ (the Leeds sleep evaluation) questionnaire, and to an improved sleep quality, sleep latency, sleep duration, and global scores evaluated by the PSQI questionnaire (Pittsburgh Sleep Quality Index) [402]. Standardised saffron extract (affron^®^; 14 mg twice daily per 28-days) improved sleep quality in adults with self-reported sleep problems. The beneficial effect of saffron was manifested by improvements in ISI total score (The Insomnia Severity Index), RSQ total score (the Restorative Sleep Questionnaire), and PSD (Pittsburgh Sleep Diary) sleep quality ratings [403]. Further investigations concerning four weeks of treatment with affron^®^ (14 mg, or 28 mg 1 h before bed) revealed improvements in sleep quality ratings assessed with Pittsburgh Sleep Diary, mood ratings after awakening (Profile of Mood States), the ISQ total score (Insomnia Symptom Questionnaire), and ISQ insomnia classifications without affecting the score of the Restorative Sleep Questionnaire and the Functional Outcomes of Sleep Questionnaire. Moreover, saffron supplementation was associated with increases in evening melatonin concentrations but did not affect evening cortisol. Sleep improvements were similar for the two saffron doses with no reported significant adverse effects. [404]. Results of the studies on the effect of crocetin on on sleep quality in healthy adult participants with mild sleep complaint assessed showed that supplementation with this bioactive compound contributes to sleep maintenance, leading to improved subjective sleep quality. This beneficial effect of two intervention periods of 14 days each, separated by a 14 day wash-out period, was manifested with an increase in an objective sleep parameters (delta power) measured using single-channel electroencephalography and improvements in the subjective sleep parameters sleepiness on rising and feeling refreshed assessed with using the Oguri–Shirakawa–Azumi Sleep Inventory, Middle-age and Aged version (OSA-MA).There were no significant differences in the other sleep parameters, including sleep latency, sleep efficiency, total sleep time, and wake after sleep onset [405].

A single study indicated that saffron odor was effective in treating menstrual distress by relieving the symptoms of premenstrual syndrome (PMS) and alleviating dysmenorrhea (menstrual pain) as well as helping to control irregular menstruation. As Fukui and co-workers claim [406], healthy woman with a normal sense of smell exposed to saffron aroma for 20 min experienced a decrease in salivatory cortisol and increase in 17-β estradiol level in both the follicular and luteal phases, which was accompanied with a decrease in anxiety measured using the State-Trait Anxiety Inventory (STAI). It was the first evidence of beneficial psychological and neuroendocrinological effects of saffron odour. 

### 4.3. Mechanism of Antidepressant Action 

**Figure 3:**
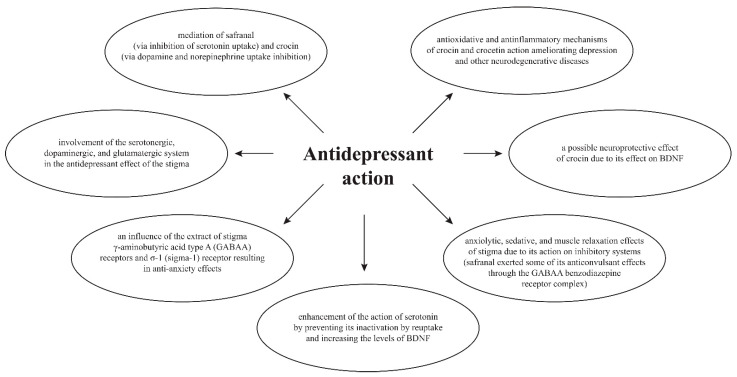
Mechanism of antidepressant action [216,228,231,293,345,364,374,375,410].

The mechanism of the in vitro and in vivo antidepressant action of *C. sativus* stigmas is attributed to e.g., crocin, which inhibits monoamine (noradrenaline and dopamine) reuptake, and safranal, which inhibits serotonin reuptake, and to their action towards GABAergic (gamma-aminobutyric acid) receptors and neurotrophic effects, e.g., through activation of BDNF (brain-derived neurotrophic factor). Stigmas of *C. sativus* (called saffron) have been demonstrated to contain an antagonist of postsynaptic NMDA (N-methyl-d-aspartate) receptors [71,407,408]. It has been proven that *C. sativus* modulates the levels of neurotransmitters, especially serotonin, in the brain by inhibiting serotonin reuptake, thereby retaining serotonin in the brain longer [409] (Figure 3).

In other studies, increased levels of CREB, BDNF, and VGF in the hippocampus were found [229,294]. There is strong evidence that VGF and BDNF are involved in depressive disorders and transcription thereof is CREB-dependent. The neuropeptide VGF enhances hippocampal synaptic activity and is involved in energy balance and homeostasis regulation. In turn, BDNF, which is widely expressed in the mammalian brain, is implicated in the survival of neurons during hippocampal development, neural regeneration, synaptic transmission, synaptic plasticity, and neurogenesis [269,270]. As reported by Asrari et al. [353], there is mediation of the P-CREB protein in cerebellum, which is consistent with the increased expression of this protein in the cerebellum described by Ghasemi et al. [229]. There is evidence that the cerebellum not only plays a role in motor function and coordination of movement, but also contributes to an important role in emotion and cognition processing. To sum up among many different proposed mechanisms explaining *C. sativus* stigma and petals effectiveness in the depression treatment, the most important is the one involving the anti-inflammatory and antioxidant effects, followed by the action on neurotransmitters in favour of the hypothesis of their deficiency in depression. Neurotrophic factors, particularly BDNF, are also of interest since they are involved, even if indirectly, in the regulation of neurotransmitters such as 5- HT, DA, glutamate and GABA and in various types of signalling such as CREB [411,412].

Studies of multiple genes have yielded some positive results regarding the usefulness of genotyping cytochrome P450 enzymes (CYP450) in the treatment of depression in groups of patients, but the choice of medications for a specific patient is not still established [413,414]. The results of investigations conducted on male Wistar albino rats receiving safranal (4, 20, and 100 mg/kg/day) or intraperitoneal injections of crocin (4, 20, and 100 mg/kg/day) indicate that both these compounds increase the total protein content and determine the metabolic activity of liver microsomal CYP450 isoforms (CYP3A, CYP2C11, CYP2B, and CYP2A) [410]. It was found that, in general, crocin markedly reduced and safranal significantly enhanced the metabolic activity of all the CYP enzymes mentioned above, except for changes in CYP2A activity induced by safranal. Therefore, the authors claim that crocin and safranal could increase the risk of interactions with co-administered substances metabolised by cytochrome P450 enzymes.

## 5. Challenges for Further Research

The scientific knowledge of the beneficial or negative impact of herbal treatment of depression is incomplete. Further investigations should focus on: (1) adequate methods of extraction of selected biologically active compounds and practical pharmaceutical applications thereof; (2) promotion of trust in phytotherapy and the use of biotechnological procedures to ensure the biodiversity of the product; (3) the use of genetic technologies to obtain good quality and high concentrations of effective phytochemicals that can be used in the future to support treatment of depression as progress in herbal psychopharmacology; (4) standard medical therapies based on herbal products, including changes in the regulations, standardisation, and financing of research on selected phytochemicals with anti-depressant effects; (5) insightful and more detailed analyses of natural compounds in terms of the basic mechanisms involved in the anti-depressant actions and justifying the application of selected plant species in the therapeutic practice of depression, taking into account antidepressant properties of these plants that have already been confirmed by scientists; (6) thorough clinical trials of selected phytochemicals—effective substances in depression treatment facilitating production of antidepressant drugs and antioxidants from these substances; (7) confirmation of the safety and efficacy of action in the treatment of depression, which will support the decision to use these compounds (as in the case of pharmaceutical drugs).

## 6. Conclusions

*Crocus sativus*, commonly known as saffron crocus, is native to the Western and Eastern Asia and Southern Europe. For centuries, it has been used in traditional Asian medicine as an agent for healing various health problems, including infections, pain, inflammation, chronic fatigue, insomnia, memory impairment, mood and personality disorders (anxiety, depression), and other mental illnesses. The medicinal activity of *C. sativus* extracts in alleviation of inflammation and central nervous system disorders, including depression, has been confirmed in the most recent basic animal (rodent) studies and human clinical trials. A number of in vitro, in vivo, and clinical trials have demonstrated that both dried stigmas and petals of *C. sativus* (water and alcohol extracts) as well as their ingredients are safe and effective antidepressants. Saffron stigma, bulbs and petals and its bioactive compounds may be considered as a potential adjuvant in the form of anti-depressant in future drug formulations. Their efficacy is similar to current antidepressant medications such as fluoxetine, imipramine, and citalopram, but fewer side effects are reported. The active compounds of aqueous and alcoholic crocus extracts exhibiting antidepressant activity include unique hydrophilic crocin carotenoids, i.e., monoglycosyl or di-glycosyl esters of crocetin, hydrophobic crocetin, and terpenoid safranal. The following mechanisms of the antidepressant action of *C. sativus* components are proposed: (1) inhibition of monoamine (dopamine, norepinephrine, serotonin) reuptake, (2) *N*-methyl-d-aspartate (NMDA) receptor antagonism, and (3) gamma-aminobutyric acid (GABA)-α agonism. Crocin acts via inhibition of dopamine and norepinephrine uptake, while safranal acts via serotonin. The antidepressant and neuroprotective effect of *C. sativus* extracts and their components is associated with anti-inflammatory and antioxidant activity. This activity is manifested by e.g., mood improvement, alleviation of anxiety symptoms, beneficial effects on learning and remembering, and a positive influence on the emotional sphere. However, due to many limitations presented in the papers cited in this protocol, there is a need for conducting further experiments to confirm the current results on the effectiveness of the antidepressant activity of *C. sativus* extract and its components and to elucidate the mechanisms of their action fully. Research reported by many authors has documented the application of herbal formulations in treatment of depression, insomnia, and anxiety, but detailed research on dosage, methods of administration and others needs to be undertaken to explore their potential in managing the health issues Although phytochemicals are natural substances and should therefore be safe, side effects have been noted due to contamination of preparation or drug interactions.

## Figures and Tables

**Table 1:** Raw material of selected plant species from various families with antidepressant properties.

Family	Species	Raw Meterial	Reference
**Monocotyledonous plants**			
Cyperaceae	*Cyperus rotundus*	Rhizome	[73]
Iridaceae	*Gladiolus dalenii*	bulb	[107,108]
	*Crocus sativus*	stigma	[70]
Xanthorrhoeaceae	*Hemerocallis citrina*	flowers	[109]
	*Hemerocallis fulva*, *H. hybrida*	flowers	[110]
	*Hemerocallis fulva*	rhizome	[76]
**Dicotyledonous plants**			
Apiaceae	*Bupleurum chinense*	rhizome	[111]
	*Ferulago angulata*	aerial parts	[112]
	*Pimpinella anisum*	fruit	[106]
Asteraceae	*Centaurea kurdica*	branches, leaves, flowers	[113]
	*Matricaria chamomilla*	flower	[114]
	*Tanacetum parthenium*	aerial parts	[115]
Campanulaceae	*Platycodon grandiflorum*	rhizome	[73]
Capparaceae	*Maerua angolensis*	stem, bark	[81]
Caprifoliaceae	*Nardostachys jatamansi*, *Valeriana jatamansi*, *V. officinalis*	rhizome, roots	[116]
	*Acacia seyal*	root bark	[81]
	*Glycyrrhiza uralensis*	rhizome	[73]
	*Saraca asoca*	bark	[117]
Hypericaceae	*Hypericum canariense*, *H. glandulosum*, *H. grandifolium*, *H. reflexum*	aerial parts	[118]
Lamiaceae	*Lavandula officinalis*	aerial parts	[119]
	*Rosmarinus officinalis*	aerial parts	[120]
	*Rosmarinus officinalis*	leaves	[121]
	*Salvia hispanica*	seeds	[122]
	*Salvia macrosiphon*, *S.mirzayanii*	aerial parts	[123]
	*Salvia officinalis*	leaves	[121,124]
	*Vitex megapotamica*	leaves	[125]
Lauraceae	*Lindera aggregata*	rhizome	[73]
	*Litsea floribunda*	leaf, stem, bark	[126]
	*Litsea glaucescens*	leaves	[95]
Passifloraceae	*Hypericum perforatum*	herbal drug	[127]
	*Passiflora edulis*	leaf	[128]
	*Passiflora incarnata*	flower	[127,129]
Polygalaceae	*Polygala paniculata*	whole plant	[130]
Rutaceae	*Citrus aurantium*	fructus	[73]
	*Citrus maxima*	fruits	[131]
	*Zanthoxylum alatum*	seeds	[132]
Solanaceae	*Solanum capsicoides*	aerial parts	[102]
Thymelaeaceae	*Aquilaria agallochum*	aquilariae resinatum lignum	[73]

**Table 2:** Total content of some classes of phytotherapeutic bioactive chemical compounds contained in the dry matter (dry weight d.w.) of different organs of *C. sativus*.

Class of Biologically Active Chemical Compounds	Type of Extract	Organ	Total Content (d.w.)		Reference
Anthocyanins	ethanolic	tepal	136.96	mg/g	[168]
	methanolic		4804	µg/g	[169]
Carotenoids	ethanolic	stigma	546.6	μg/g	[170]
		leaf	171.1		
		corm	45.64		
Flavonoid	aqueous	stigma	3.8	mg GAE/g	[171]
	ethanolic		2.9		
	methanolic		5.8		
	aqueous	stigma	3.61	mg RE/g	[170]
	ethanolic		3.53		
	aqueous	leaf	2.00		
	ethanolic		1.61		
	aqueous	corm	2.56		
	ethanolic		2.46		
	dichloromethane	flower	1.8	mg/g	[172]
	methanolic		9.2		
	water		11.2		
Phenolic compounds	aqueous	stigma	5.7–6.5	mg GAE/g	[170,171]
	ethanolic		6.3–8.3		
	methanolic		6.5		
	aqueous	corm	6.0		[170]
	ethanolic		7.1		
	aqueous	leaf	4.3		
	ethanolic		5.6		
	ethanolic	petals	3.24		[173]
Saponin	aqueous	flower	1.2	mg/g	[172]
	methanolic		3.4		
Terpenoids	dichloromethane	corm	2.8	%	[174]

Explanations: gallic acid equivalent (GAE), rutin equivalent (RE).

**Table 7:** Results of the basic (preclinical) studies of the anti-depressive effects of saffron (*Crocus sativus* L.) stigma extracts or its derivative compounds (crocin, crocetin and safranal) using animal model of depression.

Extract/Biologically Active Chemical Compounds	Treatment Groups and Route of Administartion	Biological Object	Duration of Study	Main Results	Reference
Aqueous (AE), ethanolic extracts (EE) of saffron (stigma), safranal, crocin	saline (10 mL/kg); imipramine (15 mg/kg); fluoxetine (10 mg/kg); AE: 80; 160; 320 mg/kg); EE: 200; 400; 800 mg/kg; crocin: 50; 200; 800 mg/kg; safranal: 0.15; 35; 0.5 mg/kg. Route: intraperitoneal injection (i.p.)	22–25 g male BALB/c mice	The agents were administered 30 min prior to the test. The behaviour was observed for 10 min	AE, EE, safranal (0.5 mg/kg) and crocin (50 and 600 mg/kg) reduced immobility time in FST; saffron extracts increased swimming time; both extracts, safranal at dose (0.5 mg/kg), and crocin at doses of 50 and 800 mg/kg prolonged the climbing time; AE and safranal decreased the total locomotion; ethanolic extract and crocin increased cleaning and grooming activities.	[293]
Crocin	Crocin at doses between 15 and 50 mg/kg, or diazepam (1.5 mg/kg) Route: intraperitoneal injection (i.p.)	adult 250–300 g male Wistar Albino rats	The agents were administered 30 min prior to the test. The behaviour was observed for 10 min	Crocin induced anxiolytic-like effects. Crocins, at a dose which did not influence animals’ motor activity (50 mg/kg) severely increased the latency to enter the dark compartment and prolonged the time spent in the lit chamber (light/dark test). Lower doses of crocins (15–30 mg/kg) did not substantially modify animals’ behaviour.	[230]
Saffron stigma aqueous extract and its constituents, crocin and safranal	10 mL/kg normal saline as vehicle (g 1; negative control for extract and crocin); paraffin as vehicle (g 2; negative control for safranal); diazepam at a dose of 3 mg/kg (g 3; reference group); stigma extracts at doses of 56, 80, 320, and 560 mg/kg (g IV; V; VI, and VII, respectively); crocin at doses of 50, 200, and 600 mg/kg (g VIII; IX and X. respectively); safranal at doses of 0.05, 0.15, and 0.35 mL/kg. (g XI; XII and XIII, respectively). Route of adminstration: intraperitoneal injection (i.p.)	18–22 g Razi male mice obtained from a random bred colony	Duration time according to the time of conducting ethological tests on animals. Hypnotic activity, anxiolytic activity (elevated plus maze test), locomotor activity (OFT) and motor coordination (Rotarod test) were evaluated.	The aqueous stigma extract reduced the locomotor activity dose dependently. Low doses of saffron markedly increased the time on the open arms of the maze. The aqueous extract considerably affected motor coordination. In the hypnotic test, only a dose of 0.56 g/kg of saffron increased the total sleep. Crocin showed no anxiolytic, hypnotic or myorelaxation effects. Safranal increased the total sleep time dose dependently. No involvement of crocin in the sedative or tranquiliser effects of stigma. Safranal at lower doses (0.05 and 0.15 mL/kg) decreased some locomotion activity parameters without significant effects on motor coordination, and at higher doses higher doses (15 and 35 mL/kg) showed anxiolytic effects. Saffron aqueous extract and safranal have anxiolytic and hypnotic effects.	[231]
Saffron water extract Safranal	intra-amygdala (1, 5, and 10 µg/rat) or intraperitoneal (1, 5, and 10 mg/kg) administration of the extract, safranal, or saline, respectively 5 or 30 min before electroshock stress induction.	adult 250–300 g male Wistar Albino rats	5–30 min	saffron water extract and safranal reduced metabolic and behavioural signs of acute stress without the involvement of the amygdala. As opposite to intra-amygdala-treated groups stress did not elevate the corticosterone plasma in groups that received extract or safranal intraperitoneally. Anorexia was reduced only in groups that received the extract or safranal intraperitoneally (50 s). Intraperitoneal but not intra-amygdala administration of saffron extract and safranal counteracted stress-induced increase in sniffing, rearing, locomotion, and coping time.	[347]
Crocin	vehicle (0.9% NaCl) + vehicle (g. 1); vehicle + crocins 30 mg/kg (g. 2); vehicle + crocins 50 mg/kg (g. 3); The non-selective serotonin(5-HT) receptor agonist mCPP 0.6 mg/kg + vehicle (g. 4); mCPP 0.6 mg/kg + crocins 30 mg/kg (g.5); and mCPP 0.6 mg/kg + crocins 50 mg/kg (g. 6). Each treatment grup consisted of 8 rats.	Male adult (3 months old), 250–300 g male Wistar Albino rats	The number and duration of grooming events were recorded for 20 min.	Crocins attendued the mCPP-induced OCD (obsessive-compulsive disorder)-like behaviour (excessive self-grooming) by an antagonistic action at the non selective serotonin (5-HT) 5-HT_2C_ receptor site. The pharmacological mechanism(s) that might account for the effect of crocins on compulsive behaviour has yet to be determined. Active constituents of *C. sativus* L. crocins might play a role in compulsive behaviour that often encompasses anxiety and depressive symptoms and support a functional interaction between crocins and the serotonergic system.	[348]
Saffron stigma aqueous extract (100 g of dried and milled stigma extracted with 1000 mL distilled water by maceration).	a single dose of saffron extract (5, 25, 50, 100, 150, and 250 mg/kg), fluoxetine (10 mg/kg), and/or desipramine (50 mg/kg) or saline (control group). Route of adminstration: intraperitoneal injection (i.p.)	adult 250–300 g male Wistar Albino rats (8 rats were randomly allocated for each group of the experiment)	The level of brain neurotransmitters was assaeyd thirty minutes after drug and/or extract injection; this time is considered to be sufficient for extract action	Active compounds of aqueous saffron extract triggers significant production of neutrotransmitters in brain, which is related to the effect of the extract on depression rehabilitation. Aqueous extracts of saffron (stigma) enhanced release of brain dopamine and glutamate in rats without affecting serotonin or norepinephrine concentration. These results provide a cellular basis for reports concerning the antidepressant properties of saffron extract in humans and animals. To clarify this issue the additional experiments focused on the change in dopamine concentration in brainspecific regions or serotonin concentration in the raphe nuclei are required.	[349]
Saffron (*Crocus sativus* L.) stigmas	Saffron (200, 400 and 800 mg/kg) administered alone or with concomitant administration of submaximal dose of imipramine (7.5 mg/kg) and compared with standard dose of imipramine (15 mg/kg) and normal saline (5 mL/kg) as neutral control. Route of adminstration: intraperitoneal injection (i.p.) A total of 48 animals (*n* = 48) were used for each behavioural test (FST and TST), 6 animals in each of 8 group.	20–30 g. male 3–4 months old, healthy and with normal behaviour and activity swiss albino mice	The experiment was conducted 30 min after injecting the drug. The total duration of immobility in FST and TST was recorded during 4 and 6 min, respectively.	Saffron markedly reduced immobility time. Immobility time of combination of saffron with submaximal dose (7.5 mg/kg) of imipramine was significantly reduced on comparison with control and it was comparable to standard dose of imipramine (15 mg/kg) in both FST and TST. *Crocus sativus* L. stigmas showed significant, comparable to that of imipramine, antidepressant-like activity on its own and also added to the action of a submaximal dose of imipramine. Saffron can be considered as potential antidepressant.	[350]
aqueous extract of dried saffron (*C. sativus* L.) stigma (AE);	a single dose of saffron extract (15, 30 mg/kg) (g 1 1nd 2), fluoxetine (20 mg/kg) (g. 3), and/or desipramine (50 mg/kg) or normal saline (control group; g.4). Each experimental group consisted of a 6 animals. Route of adminstration: intraperitoneal injection (i.p.) Mices were acclimatised to their environment for one week prior to experimentation	Swiss albino mice (20–25 g) of either sex	The experiment was conducted 60 min after injecting the drug. The total duration of immobility in FST and TST was recorded during 4 and 6 min, respectively.	*Crocus sativus* showed marked antidepressant activity and therefore it may be highly effective in antidepressant treatment. Saffron with 15 and 30 mg/kg significantly decreased the immobility period.	[351]
aqueous extract of saffron (AE); Crocin	aqueous extract of crocus stigma (40, 80 and 160 mg/kg/day), imipramine 10 mg/kg/day and saline (1 mL/kg) as neutral control crocin (12,5; 25; 50 mg/kg), imipramine (10 mg/kg; positive control), and saline (1 mL/kg) as neutral control. Route of adminstration: intraperitoneal injection (i.p.)	adult 250–300 g male Wistar Albino rats	3 weeks	antidepressant effects of aqueous extract of saffron and subacute administration of crocin manifested by reduced immobility time in FST. AE and crocin has antidepressant-like action by increasing CREB, BDNF, VGF neuropeptide and nd phospho-CREB (p-CREB), levels in hippocampus.	[229]
Crocin, crocetin	acute treatment: group (g) I (normal saline control gr.), gr. II–IV (crocin: 10; 20; 40 mg/kg), g. V–VII (crocetin: 10; 20; 40 mg/kg), gr. VIII (fluoxetine hydrochloride 10 mg/kg), gr. IX (desipiramine hydrochloride 10 mg/kg); sub-acute treatment: g. I (normal saline control gr.), g. II–IV (crocin: 25; 50; 100 mg/kg), g. V–VII (crocetin: 12.5; 25; 50 mg/kg), g. VIII (fluoxetine hydrochloride 10 mg/kg), and g. IX (desipramine hydrochloride 10 mg/kg) Positive control for acute and sub-acute treatment were fluoxetine (10 mg/kg) and desipiramine (10 mg/kg) Route of adminstration: intraperitoneal injection (i.p.)	male 5 month-old albino mice, 20–30 g,	in the acute treatment, all drugs were given intraperitoneally, 24; 5; and 1 h before the test; in the sub-acute treatment, the drugs were given orally (gavage) once daily for a total of 21 days	antidepressant-like effect in FST without affecting baseline locomotion due to acute treatment with crocin (40 mg/kg i.p.) and crocetin (20 and 40 mg/kg i.p.). A decrease in the immobility time only at the highest dose (100 mg/kg) of crocin administered via the sub-acute oral route, as well as a decrease in the immobility time in FST and tail suspension test (TST) after crocetin (12.5, 25, and 50 mg/kg). Crocetin had a higher efficacy than crocin pretreatment in depressive disorders. At sub-acute treatment locomotor activity (Open Field Test, OFT) and coordination (rotarod tests) were not significantly affected by crocin or crocetin.	[295]
Crocin	An organophosphate insecticide malathion (50 mg/kg/day, i.p.) alone or in combination with crocin (10, 20 and 40 mg/kg/day), imipramine (20 mg/kg/day) and vitamin E (200 mg/kg, three times a week) respectively for 14 days. Neutral control was saline. Route of adminstration: intraperitoneal injection (i.p.)	adult 200–250 g male Wistar Albino rats	2 weeks	Crocin attenuates some neurochemical and behavioural subacute exposure malathion-induced depressive-like behaviour, in particular in the FST test. Crocin ameliorated maltion-induced brain oxidative damages via antioxidant effects, which were manifested by the increased malondialdehyde (MDA) and decreased glutathione (GSH) level in cerebral corthex and hippocampus. The neuroprotective effect of crocin may be in part due to its effect on brain-derived neurotrophic factor (BDNF). Crocin and imipramine prevented the decreasing effect of malathion on the protein level of BDNF in hippocampus.	[344]
Aqueous extract of saffron (AE); Crocin	aqueous extract of crocus stigma (40 and 80 mg/kg/day), imipramine 10 mg/kg/day and saline (1 mL/kg) as neutral control crocin (12,5; 25; 50 mg/kg), imipramine (10 mg/kg; positive control), and saline (1 mL/kg) as neutral control. Route of adminstration: intraperitoneal injection (i.p.)	adult 250–300 g male Wistar Albino rats	3 weeks	Based on the increase in P-CREB protein level together with insignificant increase in the levels of VGF, CREB, and BDNF proteins after stigma extract administration the antidepressant effect of saffron in the cerebellum is related to the enhanced phosphorylation of CREB. The slight increase in protein level of the activated form of CREB indicated that the antidepressant activity of crocin is partially mediated to CREB. Other factors than BDNF and VGF neuropeptides may alter following long term crocin treatment in the cerebellum.	[352,353]
Crocin	Five rats (*n* = 11 per group) received 2 intraperitoneal injections (i.p.) as follows: group 1: DMSO plus normal saline; group 2: DMSO plus crocin (50 mg/kg); group 3: DMSO plus midazolam (1.5 mg/kg); group 4: flumazenil (3 mg/kg) plus crocin (50 mg/kg); and group 5: midazolam (1.5 mg/kg) plus crocin (50 mg/kg). Group 1 was used as the negative control. Group 2 was used to assess for anxiolytic and/or antidepressant effects of crocin. Group 3 was used as the positive anxiolytic control. Group 4 was used to determine in the crocin group the effects attributed to the benzodiazepine binding site of the GABA_A_ (γ-aminobutyric acid type A) receptor; the flumazenil was given 10 min before the crocin. Group 5 was used to assess for any interactions between midazolam and crocin. Route of adminstration: intraperitoneal injection (i.p.)	150–175 g male Sprague-Dawley rats—outbred multipurpose breed of albino rat.	All drugs were administered 30 min before the first test. The elevated plus-maze (EPM) and forced swim test (FST) test lasted 5 min each. 24 h before FST the 15 min habituation session was conducted.	Crocin attenuated the anxiolytic effects of midazolam, but did not affect psychomotor activity (elevated plus-maze EPM test). The orced swim test (FST) showed a significant increase in mean mobile time in the midazolam plus crocin group, suggesting a decrease in behavioural despair because of the interaction between crocin and midazolam. Potential limitations of this study include the one-time administration of the medications instead of the recommended two or three pretest adminstration, as well as the use of much lower doses of crocin (50 mg/kg) compared with other studies (150, 300, and 600 mg/kg—see reference Wang et al. [226]).	[354]
Crocetin	Crocetin (20, 40, 60 mg/kg) or vehicle daily for 21 days. Route of adminstration: intraperitoneal injection (i.p.). For the chronic resistant stress, rats were kept in the plexiglass restrainers for 1 h each day, for 21 consecutive days.	235 ± 15.3 g Wistar Albino rats	3 weeks	Crocetin ameliorated the chronic resistant stress-induced depressive-like behaviour by decreasing oxidative damage in the brain. Crocetin treatment reduced the immobility time in FST and increased the number of crossing in OFT test in the chronic restraint stress rats. Crocetin also reverted the levels of MDA and GSH and the activities of antioxidant enzymes (catalase CAT, superoxide dismutase SOD, glutathione peroxidase GPx and glutathione reductase GR) to the normal levels in the stressed groups. Crocetin may be an effective agent in the progression of alternative medicines for ameliorating stress-induced depression. This active constituent of saffron might inhibit behavioural modifications through alternating endocrine, oxidative, and nervous systems in rodent submitted to the long term stress.	[355]
Crocin	Lipopolysaccharide (LPS; 1.0 mg/kg twice at a 30-min interval) and/or crocin (40 mg/kg for six weeks). Route of adminstration: intraperitoneal injection (i.p.)	5–6 week-oldFemale Kunming mice	6 weeks	Crocin attenuates lipopolysaccharide (LPS)-induced anxiety, depressive-like behaviours and neuroinflammation through suppressing the NF-kB and NLRP3 inflanosome signalling pathway and promoting the M1 (neurotoxic) to M2 (neuroprotective) phenotypic conversion of microglia. This bioactive saffron constituent inhibited LPS-induced production of NO, TNF-α, IL-1β and ROS in BV-2 microglial cells as well as markedly declined the expression of oxygen and nitrogen metabolite-metabolising enzyme iNOS, NF-κB p65 and M1 marker CD16/32 but elevated the expression of M2 macrophage marker CD206 in the BV-2 cell line with decreased LPS-induced anxiety and depressive-like behaviours manifested by improved locomotor activity, reduced sucrose intake, and decreased immobility time in FST and TST. Expression of NLRP3, ASC and caspase-1 by the administration of LPS was neutralised with reductions in levels of IL-1β, IL-18 and TNF-α in the hippocampus.	[356]
crocin	Crocin at the doses of 25 and 50 mg/kg was administrated via i.p. iniection alone or combinedwith voluntary exercise. Rodents in the treatment group were subjeced to chronic restraint stress during adolescent (30–40 days old).	Adolescent, 30–40 day-old, male Wistar Albino rats	Behavioural and morphological deficits were assayed in adult (60 day-old) rodents i.e., 30 and 20 days after stress.	Physical activity and crocin prevented the detrimental symptoms of adolescent stress induced anxiety or depressive-like symptoms and dendritic morphology remodeling in prefrontal cortex in adult male rats. Plasma corticosterone levels increased at 40, but not 60 days old in stressed rats. Stressed rats exhibited enhanced anxiety levels and depression-like behaviours in adulthood accompanied by a decline in apical dendritic length in both infralimbic and prelimbic regions and dendritic branches in infralimbic region of the prefrontal cortex. Treatment with crocin, exposure to wheel running activity, and the combined interventions alleviated both behavioural and morphological deficits induced by adolescent stress. These treatments exerted positive neuronal morphological effects in the prefrontal cortex in non-stressed animals. Exercise as a non-pharmacological intervention and crocin treatment during the pre-pubertal period can protect against adolescent stress-induced behavioural and morphological abnormalities in adulthood.	[357]
Crocin	In a chronic unpredictable mild stress (CUMS) mouse in vivo model used to assessment of depression-like behaviour in OFT, TST, FST, SPT, and NSF tests the mice were assigned randomly to four groups (*n* = 10 each): control (sodium chloride 0.9%), CUMS, CUMS plus crocin 30 mg/kg (intragastric administration, i.g.), and CUMS plus fluoxetine 20 mg/kg (intraperitoneal injection, i.p.). In a corticosterone (CORT) in vitro model of PC12 set up to explore the antidepressant mechanism of crocin the PC12 cells were pretreated with gradient concentration of crocin (12.5, 25, and 50 μmol/L) for 1 h and then stimulated with CORT (200 μmol/L) for 24 h. Cell survival was detected by Hoechst staining and 3-(4,5-Dimethylthiazol-2-yl)-2,5-diphenyl tetrazolium bromide MTT assay.	18–24 g, 8–10 weeks old male Balb/cJ albino mice	4 weeks From the third week mice were acclimatised for a week before the CUMS procedures were initiated—crocin and fluoxetine were administered once daily until the CUMS paradigm end.	Crocin significantly alleviated CUMS induced depression-like behaviours, reversed the decrease of body weight and elevation of serum CORT, and protected PC12 cells against CORT-induced injury by increasing the expression of pituitary adenylate cyclase-activating polypeptide (PACAP) and thereby enhanced the photophosporylation of its downstream ERK and CREB signalling pathways. It is thus conceivable that PACAP will be an important target for antidepressant treatment.	[358]
Crocin-I	crocin-I (20 and 40 mg/kg for 2 weeks, 4 weeks) administered orally after induction of depression with 20 mg/kg corticosterone by subcutaneous injection in mice	8 week-old male C57BL/6J mice	2 weeks	crocin-I exerted severe antidepressant effects in a model of chronic corticosterone (CORT)-induced depression, as evidenced by the attenuation of depression-like behaviours in the OFT, FST, and TST which was due to the suppression of neuroinflammation (IL-1β) and oxidative stress in the hippocampus. The oral administration of crocin-I (40 mg/kg) decreased the CORT-induced nicotinamide accumulation in the liver to improve the synthesis of NAD+, thereby stimulating the activity of SIRT3 deacetylase to elevate the activity of antioxidants such as superoxide dismutase 2 and glutathione reductase. Crocin-I reduced the levels of oxidative damage markers (ROS and MDA) to rescue impaired mitochondrial function caused by CORT treatment, which was represented by the electron transport chain and oxidative phosphorylation normality, and thus rescued ATP production to the level of that in wild-type mice. This results provide new information on the mechanism of action of crocin-I on depression-like behaviour and oxidative stress in perceived conditions-stressed individuals.	[359]
Crocin	Crocin (50 mg/kg; group 1), anti-inflammatory medicine Dexamethasone (Dex; 2 mg/kg; group 2—positive control), or the activator of Phosphatidylinositol 3-kinase (PI3K) Insulin-like growth factor 1 (IGF-1; 2 mg/kg; group 3) administered to mices randomly exposed to cigarette smoke for 7 weeks to induce chronic obstructive pulmonary disease (COPD) depression model; cigarette smoke-exposed group No. 4; fresh air-exposed control group No. 5. Each of five experimental group consisted of 8 individuals (*n* = 8). To establish of cigarette smoke COPD model mice were exposed to cigarette smoke of 5 3R4F Kentucky reference cigarettes (without filter, University of Kentucky, Lexington, KY, USA), one after another, four times a day (total of 20 cigarettes per day). Animals were alternately exposed to the smoke for 30 min with a smoke-free interval for 30 min. The procedure lasted for 7 consecutive weeks Route of administration: vehicle, Dex (2 mg/kg) or IGF-1 (2 mg/kg) via a single i.p. injection or 50 mg/kg of crocin orally 1 h before exposure to cigarette smoke once a day	7–8 week male C57BL/6 mice	Behavioural test were determined at 24 h after the last cigarette smoke exposure. The total duration of the OFT, FST, and TST was 6, 5, and 4 min, respectively. SPT—1% sucrose solution was given for 1 h after 12 h period with no water and food	Crocin alone or with concomitant administration of Dex or IGF-1 improved cigarette smoke-induced depression-related behaviours. This bioactive compound of saffron markedly reveresd body weight loss, sucrose preference, and elevation of immobile time in TST and FST as well as improved exploratory behaviour and general activity in OFT. Crocin markedly inhibited the number of inflammatory cells (macrophages, neutrophils, and lymphocytes), suppressed the infiltration of peribronchial inflammatory cells, and strongly reduced the concentration of proinflammatory cytokines in hippocampus in bronchoalveolar lavage (BAL) fluid and lung tissue. Crocin blunted cigarette smoke-induced IκB phosphorylation and degradation, and NF-κBp65 nuclear translocation. IGF-1, an activator of PI3K, abrogated the effect of crocin against cigarette smoke-induced activation of the NF-κB pathway. Crocin protected against cigarette smoke-induced COPD with comorbid depression via the inhibition of the inflammatory response via PI3K/Akt-mediated Nuclear factor-κB (NF-κB) signalling. Crocin exhibits therapeutic potential in inflammatory lung disease with comorbid depression.	[360]
Kaempferol	Treatment and stress procedure: After 1 week of adaptation, all C57 mice were divided randomly into five groups (*n* = 10/group): control (non-stressed), chronic social defeat stress CSDS, CSDS + 10 mg/kg kaempferol, CSDS + 20 mg/kg kaempferol, CSDS + 10 mg/kg fluoxetine. Chronic social defeat stress procedure: adult C57 mice were functioned as intruders and the aggressive CD1 were retired breeders. During the 10 days the C57 mice were exposed to attack othe aggressive CD1 mice for up to 10 min each/day. The stressed C57 mices displayed submissive behaviours including fleeing, trembling, immobility, crouching and upright posture (8–10 min). On the day 11, SPT, social interaction test and TST were used to screen the successful models, which were delivered by drugs. Selected stressed mice were divided into various groups receiving fluoxetine, a morpholine-containing LY294002 that is a strong phosphoinositide 3-kinases (PI3Ks) inhibitor used to investigate the role of AKT/β-catenin signaling in the antidepressant effects of kaempferol, and vehicle for 28 days. The behavioural tests were preformed from day 29 to day 34 and at the day 35 the mices were sacrificed. All drug were administered via direct intraperitoneal injection (i.p.)	inbred C57BL/6J (C57) 8 week-old mice and 8 week-old CD-1 outbred mouse line derived from the original colony of Swiss mice	Duration of the experiment: 35 days. The SPT lasting for 4 days was carried out. The social interaction test consisted of “target absent” and “target present” trial and the each trial lasted for 5 min. In the “target absent” trial, each mouse explored in an open-field apparatus with fixed plastic enclosure freely and the interaction zone was predefined. In the “target present” trial, each mouse was returned to the same open-field apparatus with an unfamiliar CD1 mouse. TST—recorded for 5 min and reflected the depressive state	Kaempferol and fluoxetine therapy conspicuously ameliorated behavioural dysfunction in depression as well as attenuated the malonaldehyde (MDA) and protein carbonylation contents, increased oxidative stress markers (superoxide dismutase SOD, glutathione peroxidase GPx, catalase CAT, glutathione s-transferase GST), reduced the concentrations of proinflammatory markers (IL-1β and TNF-α), inhibited CD-11b mRNA levels in the prefrontal cortex of CSDS mice, as well as enhanced AKT/β-catenin pathway. LY294002 appeared to partly inhibit kaempferol-mediated protective effects in the CSDS mice. Antidepressive effects of kaempferol are mediated by reduction of oxidative stress, proinflammatory cytokines and up-regulation of AKT/β-catenin cascade activity in the prefrontal cortex of CSDS mice. Kaempferol might be a promising, effective, and safe food-medicine agent for depression treatment.	[361]
Crocin-I	crocin-I (40 mg/kg) administered orally for six weeks to mices exposed to 4 week chronic restraint stress CRS).	8 weeks old male C57BL/6J mice	6 week	Crocin-I alleviated CRS-induced depression in mice. This effect was accompanied by reduction of lipopolysaccharide (LPS), Interleukin-6 (IL-6) and tumor necrosis factor-α (TNF-α) levels in serum and TNF-α expression in the hippocampus, and the increase in the hippocampal brain-derived neurotrophic factor (BDNF). As revealed 16 s rRNA sequencing, crocin-I mitigated the gut microbiota dysbiosis in depressed mice as represented by the decreased abundance of *Proteobacteria* and *Bacteroidetes*, *Sutterella* spp. and *Ruminococcus* spp. and increased abundances of *Firmicutes* and *Lactobacillus* spp. Gas chromatography-mass spectrometry revealed that crocin-I reversed the decreased levels of short-chain fatty acids (SCFAs) in faeces of depressed mice as well as improved the impaired intestinal barrier by increasing expression of Occludin and Claudin-1, which contributed to the decreased LPS leakage. Crocin-I effectively alleviated depression-like behaviour, likely dependent on the gut microbiota and its modulation of intestinal barriers and SCFAs.	[362]
Crocin	Crocin (15, 30, 40 or 50 mg/kg/day) administered by the intragastric route for 10 days after 1-methyl-4-phenyl-1,2,3,6-tetrahydropyridine (MPTP)-induced (30 mg/kg daily for 7 days) subacute Parkinson’s disease depression.	18–24 g, 8–10 weeks old male Balb/cJ albino mice	The behavioural tests were carried out on the 18th day and biochemical assays on the 20 day	Crocin treatment alleviated the MPTP-induced depressive-like behaviour assessed in FST in via protection of the dopaminergic (DA) projection neurons in the ventral tegmental area (VTA) through activating mammalian target of rapamycin (mTOR), and improving the neural synaptic plasticity of medial prefrontal cortex (mPFC).	[363]
affron^®^, a patented, obtained at industrial scale, standardised commercial saffron (stigma) extract containing ≥3.5% of total bioactive compounds safranal and crocin isomers	Animals were randomly assigned to one of the three of the experimental groups: oral affron^®^, intraperiotoneally administered affron^®^ (ip) and vehicle control—normal saline i.p. (10 rats per group). In the oral group, a single dose of affron^®^ (200 mg/kg in a volume of 2 mL/kg) was administered via intragastric route at the beginning of the experiment, and then for the next 20 days, standardised stigma extract was dissolved in in drink tap water. In the intraperitoneal group rats received a daily dose of affron^®^ (50 mg/kg). Behavioural tests were performed on the first day of the experiment in order to assess the acute effects of the treatment, and also after the chronic treatment.	300–350 g. adult male Wistar rats	Duration of the experiment: 3 weeks. Acute condition—rats were tested a first time in the EPS and FST 30 and 60 min., respectively after the first administration of affron^®^. The animals were assayed in SPT test, used as complementary test for anhedonia, on the 17th day of the experiment 30 min after affron^®^ administration. On the 21st day of the experiment, the animals were assessed for the second time at the EPM and 30 min later at FST—chronic treatment.	Oral affron^®^ improved anxious/depressive state of rats—enhanced consumption of a sweet solution, as well as increased certain escape responses FST, but was equally as ineffective either orally administered or by the intraperitoneal anxiety-related behaviour using elevated plus-maze test (EPM). The evidence of the antianhedonic, and mild antidepressant actions of a 50 mg/kg acute i.p. dose and a 200 mg/kg oral dose of a standardised saffron extract of affron^®^, when administered acutely or repeatedly, orally, has been provided. These results open new fields for the possible application of affron^®^ to prevent negative emotional states or as a co-adjuvant therapy in the treatment of depression.	[364]
Crocin	Treatment and stress procedure: female mice were randomly assigned into stressed and nonstressed groups; the stressed mice received a chronic mild stress procedure, i.e., 6 h restraint stress in a 50 mL centrifuge tube daily, combined with overnight illumination twice a week for three weeks. Females were mated with naïve males. Stressed females developed depression-like behaviour postpartum. The offspring of prepregnancy stressed nonstressed (naïve) females were defined as prenatal stress mice (PNS) and control group. The two groups of offspring were housed with dams until three weeks postnatally. PNS and control group were tested for depression-like behaviours at postnatal day (PND) 28 (juvenile) and 60 (adulthood). Drug administration: Ketamine (30 mg/kg, i.p.) or crocin (10, 20, 40 mg/kg, intragastrically) or saline control was administered i.p. 24 h before the behaviour tests. In the time-course test, crocin (40 mg/kg) was administered 0.5, 2, 24, and 72 h prior to the behavioural tests, respectively. JMV2959 (12 mg/kg, i.p.)—an antagonist of growth hormone secretagogue receptor type 1a (GHS-R1a) and LY294002 (50 mg/kg, i.p.)—a highly selective inhibitor of phosphatidylinositol 3 (PI3) kinase (PI3k) were administered 30 min before crocin (40 mg/kg). Growth hormone secretagogue receptor (GHSR) and phosphoinositide 3-kinase (PI3K) inhibitors were used to test their effects in antidepressant-like effect of crocin.	18−24 g., six to eight weeks old, female and male Balb/cJ mice	Duration of the experiment: 3 weeks. OFT was used to assess the locomotor as well as the exploratory behaviour in an open area and the locomor activity was recorded for five minutes FST immobility time was measured for 4 min. SPT 2% sucrose solution were given for 1 h after 18 h period of water and food deprivation; novelty suppressed feeding (NSF) test latency to feeding was measured for 5 min	Rapid and prolonged antidepressant-like effect of crocin associated with GHSR-mediated hippocampal plasticity-related proteins in prenatal stress exposed mice was demonstrated. Crocin activated the hippocampal GHSR–PI3K signalling and induced a rapid and enduring antidepressant effect, which is similar to the effect of ghrelin—a 28-amino-acid peptide feeding peptide recognised as an endogenous ligand for the growth hormone secretagogue receptor 1a (GHSR-1a) and identified as an important mediator in the pathology of mood disorders. The study demonstrated the adverse effect of prenatal stress as evidenced by significant depressive-like behaviours in mice. Crocin produced a fast and long-lasting antidepressant-like effect in PNS mice and restored the impaired the expression of hippocampal synaptic plasticity-associated proteins via modulation of GHSR–PI3K signaling. This signalling pathway contributes to the antidepressant properties of crocin, as the inhibition of both GHSR and PI3K abolished its unique effect. The GHSR inhibitor JMV2959 was blocked by crocin. These findings illuminated the promising antidepressant-effect of crocin as a novel antidepressant agent.	[365]
Crocetin	Oral daily administration of crocetin (20, 40, 80 mg/kg), fluoxetine (20 mg/kg) or distilled water	Two week-old Institute of Cancer Research (ICR) mice stressed by immobilising them for 6 h per day for 28 days	TST and OFT test were performed 60 min after the treatments and afterwards mitogen-activated protein kinase kinase/extracellurar signal-regulated kinase (MEK/ERK) pathways markesrs and gut microbiota were assayed.	Crocetin ameliorated chronic restraint stress-induced depressive-like behaviours in ICR mice (TST and OFT). This bioactive compound of saffron stigma markedly attenuated the elevated levels of the expression of mitogen-activated protein kinase phosphatase-1 (MKP-1), the precursor of brain-derived neurotrophic factor (proBDNF), alanine and aspartate transaminase as well as increased the serum level of dopamine and phosphorylated cAMP response element-binding (CREB) (enzyme-linked immunosorbent assay ELISA kits; Immunoenzymatic test ELISA). Histopathological analysis showed that crocetin suppressed hippocampus injury in restraint stress mice by protecting neuronal cells. Immunofluorescent and Western-bolt assays revealed elevated expression levels of ERK1/2, CREB and inhibited expression levels of MKP-1, proBDNF in the hippocampus. High-throughput sequencing showed that the composition of intestinal microbiota of the crocetin group partially recovered and was quite similar to the control group. Therefore crocetin showed neuroprotective properties and reduced the effects of chronic stress-associated barin damage by regulating the MKP-1- ERK1/2-CREB signalling and intestinal ecosystem.	[366]
Saffron (stigma) extract	Rats were treated with morphine (10 mg/kg, scubcutaneous injection twice daily) for 10 days. Animals received saffron extracts (60 mg/kg, i.p.) daily, during the induction of morphine dependence and/or withdrawal. Rats were tested for spontaneous withdrawal signs, anxiety using the EPM test, depression using SPT test, and voluntary morphine consumption using a two-bottle choice paradigm, and then challenged with morphine (1 mg/kg, ip) to evaluate locomotor sensitisation and cerebrospinal fluid serotonin levels.	300–350 g. adult male Wistar rats	10 days	Saffron extract during induction of morphine dependence did not affect anxiety and depression-like behaviours, but markedly decreased the severity of withdrawal signs. Saffron extract during morphine withdrawal resulted in the percentage increase (or ratio) of open/total arm entries, higher levels of sucrose preference, a lower morphine preference ratio as well as decrease in locomotor activity and an increase in the cerebrospinal fluid serotonin levels in rats challenged to morphine. Saffron extract may exert a protective effect against morphine-induced behavioural sensitisation in rats, probably through increasing serotonin levels.	[367]
Safromotivines^TM^ standardised saffron extract (Saf’ ‘Inside^TM^) containing more than 25 active compounds, including safranal (>0.2% according to U-HPLC method)	saffron extract Safr’Inside™ acute (*n* = 10) and chronic (*n* = 10) treatment (6.25 mg/kg per os) or its vehicle (water; *n* = 10) orally administered in the morning. The dose of saffron extract was calculated based on the ratio given by the United States Food and Drug Administration to reflect, for a mouse, the equivalent of the effective dose classically administrated to humans, namely 30 mg/day. For acute experiments, the solutions were administered 30 min before the behavioural assessment. For the chronic experiment, mice received one gavage per day for 4 weeks, the last being done 3 h before the behavioural test. When completing chronic treatment after behaviourral tests, mice were sacrificed and plasmatic corticosterone levels (enzyme immunoassay) as well as brain monoamines system measurements (HPLC; RT-qPCR) were performed including the levels of dopamine (DA), 3,4-dihydroxyphenylacetic acid (DOPAC), homovanillic acid (HVA), serotonin (5-HT) and 5- hydroxyindolacetic acid (5-HIAA) in the frontal cortex, striatum, and hippocampus together with gene expresion markers of serotonin and dopamine system.	8 weeks old male C57BL/6J mice	Acute condition—mice were tested in the 6 min FST a first time 30 min after the first administration of saffron extract or water. Duration of swimming, climbing, and immobility was determined during the last 4 min of the test. Then, after 3 weeks of daily treatment, rodents were tested in a light–dark test (LDT) and again in the FST 1 week later—chronic conditions.	Saffron extract mitigated depressive-like behaviour in the FST through neurobiological modifications, particularly through an increase in serotonergic and dopaminergic neurotransmission, suggesting that Safr’Inside™ may share common targets with conventional pharmacological antidepressants. Further studies are needed to deeply understand how Safr’Inside can modulate the activity of these systems, as well as their causal role in the observed effects, and to test the contribution of other known pathophysiological bases of mood disorders. Safr’Inside administration does not reduce anxiety-like behaviour, as assessed in chronically treated mice exposed to a classical and pharmacologically validated rodent test of anxiety, the LDT.	[368]

Explanations: ASC—apoptosis-associated speck-like protein containing a caspase recruitment domain; BALB/c mice—albino, laboratory bred strain of the house mouse; BDNF—brain-derived neurotrophic factor; CREB—response element-binding protein; DA—dopamine; DMSO—dimethyl sulfoxide (vehicle); EPS—Elevated Plus Maze Test; ERK—extracellular regulated protein kinases; FST—Forced Swimming Test; GSH—glutathione; IL-1β—Interleukin-1β; iNOS—Inducible nitric oxide synthase; MDA—malondialdehyde (MDA); NF-κB (nuclear factor kappa-light-chain-enhancer of activated B cells); NF-κBp65—NF-κB nuclear transcription factor subunit p65; NLRP3—The Nod-Like Receptor (NLR) family pyrin domain-containing protein 3; NO—nitric oxide; OFT—Open-Field Test; PC12—a cell line derived from a transplantable rat pheochromocytoma which catecholamine type cells synthesise, store and release norepinephrine and dopamine; TST—Tail Suspension Test; TNF-α—tumor necrosis factor-α; SPT—sucrose preference test; NSF—novelty-supressed feeding test; BDNF—brain-derived neurotrophic factor; VGF—non-acronymic neuropeptide.

**Table 8:** Results of the basic (preclinical) studies of the antidepressive effects of *Crocus sativus* L. of corms and petals, apart from stigma extracts or kaempferol using animal model of depression.

Extract/Biologically Active Chemical Compounds	Treatment Groups (g.)	Biological Object	Duration of Study	Main Results	Reference
Petals					
Kaempferol	Fluoxetine (20 mg/kg) used to treat depression or obsessive-compulsive disorder as a positive control and kaempferol (100; 200 mg/kg in mice and 50 mg/kg in rats). Route of adminstration: intraperitoneal injection (i.p.)	25–30 g male albino mice, 200–220 g Wistar rats	After two minutes swimming, behavioural activities were evaluated during four min.	Significant reduction of immobility behaviours in the rodents used for evaluation of the antidepressant activity of kaempferol, as in the case of fluoxetine	[321]
Aqueous (AE), ethanolic (EE)	Treatment groups (9 g/kg): AE of stigma: 0.8; 0.32; 0.56; 0.64; AE of petal: 1.4; 2.5; 3.6; EE of stigma: 0.2; 0.8; 1.4; 1.6, EE of petal: 0.8; 1.4; 3.6. Route of adminstration: intraperitoneal injection (i.p.)	25–30 g male albino mice	After fifteen minutes of the pretest session 24 h later the mice were exposed to the experimental condition for six min.	Stigma and petals aqueous extract showed antidepressant effects as effective as an imipramine dose of 15 mg/kg. in reduce of the immobility time in FST	[175]]
Corms and stigmas					
aqueous ethanol extract of corms aqueous extract of stigma	Crude aqueous ethanol extract of corms fractionated on the basis of polarity at the doses of the dried extract: 150; 300; 600 mg/kg of body weight for fractions, compared to the positive drug control fluoxetine (100 mg/kg) (g. 1); Aqueous and ethanolic stigma extract of *C. sativus* compared to the positive drug control fluoxetine (100 mg/kg) (g. 2). Route of adminstration: intraperitoneal injection (i.p.)	18–22 g ICR male albino mice, aged 6–11 weeks	The animals were trained 24 h before the test (15 min) and the test was performed for 5 min.	The petroleum ether fraction and dichloromethane fraction of corms at doses of 150, 300, and 600 mg/kg as well as the aqueous stigmas extract exerted antidepressive effects in the behavioural models (FST, TST, OFT). Antidepressant-like properties of aqueous stigma extracts are due to crocin 1, and by means of a gas chromatography–mass spectrometry technique, twelve compounds of the petroleum ether fraction were identified. Therefore, the low polarity parts of *C. sativus* corms should be considered as a new plant material for curing depression, and further studies regarding antidepressive-like activities of chemical compounds isolated from the two fractions and mechanism of action are highly recommended.	[226].
Corms, stigmas and petals					
Corm (CEE), leaf (LEE), petal (PEE, and stigma (SEE) ethanolic extracts of saffron	6 group of mices (*n* = 6 per group) received: 1 mL/kg of 0.9% saline (g.1.—negative control); a dose of 10 mg/kg standard drugs (aspirin for analgaesic and anticoagulant, diclofenac potassium for anti-inflammatory, and fluoxetine HCl for antidepressant assay (g.2—positive control); dose of 800 mg/kg CEE, LEE, SEE, and PEE, respectively (g.3, g.4, g.5). Route of adminstration: oral gavage	either sex, adult, 30–40 g, albino mice (Swiss strain)	saline and fluoxetine HCl, along with saffron extracts were administered 30 min before the test. The forced swimming test (FST) test lasted 6 min. 24 h before FST the 15 min habituation session was conducted with each mouse	petal and stigma extracts showed antidepressant effects by reducing immobility, while corm and leaf extract indicated moderate to mild antidepressant efficacy. Apart from this antidepressant action, petal and stigma ethanolic extracts were evidenced as a safe natural remedy to threat pain (Hot Plate Analgesic Test), inflammation (Carrageenan-induced Hind Paw Edema Test) and the coagulation system. These pro-health effects are related to intrinsic active compounds, mainly carotenoids and flavonoids, found in the highest amounts in stigma and petals, respectively. Further epidemiological investigations, laboratory research, and clinical trials are needed to isolate the pharmacologically active molecules that contribute to the therapeutic effects and to explicate the possible mechanism of action and effect of the plant on various critical illnesses and medicinal formulations.	[205]

Explanations: g.—group; FST—Forced Swimming Test; OFT—Open-Field Test; TST—Tail Suspension Test.

**Table 9:** Results of clinical studies on the antidepressant effect of bioactive chemical compounds contained in *C. sativus* L. stigmas administered as capsules, tablets, or extracts.

Extract/Biologically Active Chemical Compounds	Treatment Group (g.)	Participants	Exclusion Criteria	Design and Duration of Study	Main Results	Limitations of Studies and Recommendations for Future Investigations	Reference
Saffron capsule	saffron capsule 30 mg/day (g. 1) or imipramine 100 mg/day (g. 2)	30 adult, 18–55 age, outpatient clinic of Roozbeh Psychiatric Hospital who met the Diagnostic and Statistical Manual of Mental Disorders, 4th edition (DSM IV) for major depression based on the structured clinical interview for DSM IV with a baseline Hamilton Rating Scale for Depression (HAM-D 17-item) score of ≤18.	current cognitive disorder in the last year; current or past history of bipolar disorder, schizophrenia and schizotypal personality disorder; treatment with any psychotropic medications for at least 4 weeks before study entry; significant risk of suicide; pregnant women or women not using medically accepted means of birth control.	6 week double-blind randomised single center trial	saffron was as effective as imipramine in the treatment of mild to moderate depression. In the imipramine group anticholinergic effects such as dry mouth and also sedation were observed much more often than in saffron- group	lack of a placebo group; using only a fixed dose of saffron; the small number of participants and short period of follow up	[177]
Saffron capsule	Saffron capsule 30 mg[sol]day (g.1; *n* = 20; 11 male and 9 female) or placebo (BD) (g.2; *n* = 20; 11 male and 9 female)	40 adults, 18–55 age, outpatient clinic of Roozbeh Psychiatric Hospital who met the Diagnostic and Statistical Manual of Mental Disorders, 4th edition (DSM IV) for major depression based on the structured clinical interview for DSM IV with a baseline Hamilton Rating Scale for Depression (HAM-D 17-item) score of ≤18.	current cognitive disorder in the last year; current or past history of bipolar disorder, schizophrenia and schizotypal personality disorder; treatment with any psychotropic medications for at least 4 weeks before study entry; significant risk of suicide; pregnant women or women not using medically accepted means of birth control.	6 week double-blind, randomised placebo-controlled trial	significantly better outcome of saffron on the Hamilton depression rating scale than the placebo with no significant differences in the two groups in terms of the observed side effects. Therefore saffron may be efficient in the treatment of mild-to-moderate depression.	Too small for a large-scale trial and short period of follow up	[178]
Saffron capsule	A saffron capsule 30 mg/day (g. 1; *n* = 20; Male/Female 11/9) or a capsule of fluoxetine 20 mg/day (g. 2; *n* = 20 Male/Female 9/11).	40 adults, 18–55 age, outpatients who met criteria for MDD based on a structured clinical interview for Diagnostic and Statistical Manual of Mental Disorders, fourth edition, baseline HDRS score of ≤18 and with mild-to-moderate depression.	cognitive disorder in the past year, current/past history of bipolar disease, schizophrenia, borderline personality disorder, suicide risk, pregnancy	6 week randomised double-blind single-centre trial	comparable efficacy of saffron (stigma) and fluoxetine in treatment of mild-to-moderate depression without significant differences observed in the side effects	lack of a placebo group; using only a fixed dose of saffron; the small number of participants and short period of follow up	[338].
Saffron capsule	capsule of *C. sativus* 30 mg/day (15 mg twice a day, group 1; *n* = 25) or capsule placebo (twice a day; group 2; *n* = 25) for two menstrual cycles (cycles 3 and 4). 24 subject assigned to saffron group and 23 subject within placebo group completed the trial.	Fifty women aged 20–45 years with regular menstrual cycles and experience of premenstrual syndrome symptoms for at least 6 months. Each capsule had dried extract of petal of *C. sativus* (15 mg), lactose (filler), magnesium stearate (lubricant) and sodium starch glycolate (disintegrant).	major physical or psychiatric disorder or substance abuse in the previous 6 months	Double-blind, randomised and placebo-controlled trial; duration of the trial—menstrual cycles 1–4 by women	saffron was effective in relieving symptoms of premenstrual syndrome symptoms. A significant difference was observed in efficacy of saffron in cycles 3 and 4 in the Total Premenstrual Daily Symptoms and Hamilton Depression Rating Scale. Saffron is highly efficient in the treatment of premenstrual syndrome symptoms. A tolerable adverse effects profile of saffron may well confirm the application of saffron as an alternative treatment for premenstrual syndrome symptoms.	using only a fixed dose of saffron; the small number of participants and short period of follow up should be considered, and further research in this area in particular comparison with an active agent such as fluoxetine is needed.	[369]
Saffron capsule evaluated by crocin value. Each capsule contained 1.65–1.75 mg crocin.	A saffron capsule contained 15 mg of *Crocus sativus* (stigma) dried extract twice daily (30 mg each day; group 1) or starch placebo capsule (group 2) for 4 weeks. Male to female ratio in saffron and placebo group was 7:3 and 5:5, respectively.	20 adult, 18 to 55 year-old, outpatients with the diagnosis of MDD according to the Diagnostic and Statistical Manual of Mental Disorders: 4th Edition (DSM-IV-TR) receiving an selective serotonin reuptake inhibitor (SSRI) for at least 1 month prior to the the study and using at least one medically accepted mean of birth control during the study.	pregnancy or lactation, history of allergy to saffron or multi drug reaction, any history of blood disorders (anaemia, haemophilia), or other severe medical conditions (cardiovascular, renal, hepatic, pulmonary, metabolic or endocrine diseases), history of seizure, substance abuse in the previous 6 months, active peptic ulcer, and taking any medication during the study (except for alprazolam up to 0.5 mg per day).	6 week, paralell, double-blind, randomised placebo-controlled trial	Saffron as an add-on medication to SSRIs treatment in MDD for 4 weeks did not cause any adverse effect in laboratory parameters including blood cells and coagulation factors, fasting blood sugar, lipid profile markers (triglyceride, total cholesterol) as well as liver and renal functions. It provides evidences of safety concurrent intake of saffron and SSRIs.	small sample size and short period of follow up. The more frequent blood testing is suggested.	[370]
Hydro-alcoholic extract of *C. sativus* stigma	fluoxetine 30 mg/day (20 mg morning, 10 mg noon) and capsules of saffron 40 mg/day (b.i.d) (g. 1) or fluoxetine 30 mg/day and Saffron 8o mg/day (g. 2)	60 adult patients who met the diagnostic and statistical criteria for mental disorders based on the Hamilton Depression Rating Scale (HDRS), Diagnostic and Statistical Manual of Mental Disorders, Fourth Edition (DSM-IV), and structured clinical interviews	Diabetes, drug abuse, hypertension, cardiovascular and autoimmune diseases; astma, any infection or illness over the past month; treatment with antiplatelet or anticoagulant medications	6 week double-blind randomised trial	efficacy and safety of saffron in treatment of mild-to-moderate depression, especially in the group receiving 80 mg saffron capsules	lack of a placebo group; too small of a scale	[371]
Saffron capsule	capsule of dried saffron extract 30 mg/day (g. 1; *n* = 20 Male/Female 9/11) or fluoexetine 40 mg/day (g. 2; *n* = 20, Male/Female 6/14)	40 male and female patients aged 20 to 65 years with diagnosis of mild-to-moderate depression (HDRS score of 14–22) who had undergone percutaneous coronary intervention in the last six months. Patients with severe depression were included only if they were suffering from significant depressive symptoms forcing them to seek treatment	any other psychiatric disorder on the DSM-IV axis I or II based on structured diagnostic interview; treatment with psychotropic medications; a high risk of suicide (score ≥2 on the suicide item of HDRS); psychotherapy within 4 weeks or electroconvulsive therapy within 8 weeks prior to entry; substance abuse or dependence (other than nicotine) within 3 months, serious or life-threatening illness, thyroid disease, hepatic or renal dysfunction, hypersensitivity to fluoxetine or herbal compounds, pregnancy, lactation, and oral contraception use.	6 week randomised double-blind placebo-controlled trial	comparable antidepressant efficacy of short-term therapy with saffron capsules in and fluoxetine treatment in post percutaneous coronary intervention patients based on HDRS and evaluation of adverse events.	a relatively small sample size and a short observational period; lack of a particular probe to evaluate and compare the impact of saffron and fluoxetine on cardiovascular parameters; patients suffering from depression due to cardiovascular diseases other than coronary artery disease (CAD) were not included in the study	[372]
Crocin tablets	selective serotonin reuptake inhibitor, (fluoxetine 20 mg/day or sertraline 50 mg/day or citalopram 20 mg/day) together with a placebo, b.i.d. (g. 1) or the same treatment as in g. 1 with crocin tablets (30 mg/day; 15 mg b.i.d instead of placebo (g. 2)	40 patients, 24–50 in age, with MDD examined according to the Structured Clinical Interview of the fourth revision of the Diagnostic and Statistical Manual of Mental Disorders (DSM-IV)	depression with psychotic features; psychotropic medication treatment in recent months; substance dependence; suicide risk, disorders: organic, neurological, personal, cognitive, psychotic,	4 week randomised double-blind placebo-controlled pilot trial	crocin is a particularly effective therapeutic adjuvant in treatment of MDD patients. Crocin improved scores of psychiatric tests including beck depression inventory (BDI), beck anxiety inventory (BAI), and general health questionnaire (GHQ). Crocin amplified the effects of selective serotonin reuptake inhibitors (SSRIs) in the treatment of patients with mild-to-moderate depression without substantial side effects. The antidepressant effects of saffron extract is probably attributed to crocin as the main antioxidant constituent in saffron stigmas.	poor patient compliance with medications and short trial period; small sample size, and self-reported assessments	[216]
Saffron capsule	25 mg of dried saffron extract (g. 1; *n* = 50) or 5 mg of diazepam as a control (g. 2, *n =* 52).	102 male, 18–50 years old patients, candidates for herniorrhaphy operation being in I and II classes of the The American Society of Anesthesiologists (ASA) physical status	allergy to saffron, its products, and tranquilisers	8 month double-blind controlled randomised trial	greater effectiveness of dried saffron extract on soothing anxiety than diazepam (based on Speillberger State-Trait Anxiety Inventory (STAI) before intervention and 3 h after administration, immediately after entering the surgery room).	financial limitations for preparation of expensive saffron capsules; various elements of saffron need to be studied through clinical assessments	[345]
Saffron capsule	50 mg saffron capsule (g.1; *n* = 20; Male/Female 8/22) or a placebo (g.2; *n* = 20 Male/Female 10/20) twice daily for 12 weeks. 30 subject from control and 24 form the treatment group completed the trial.	60 adult, 18–70 year old patients with mild-to-moderate mixed anxiety and depression diagnosed on the basis of the Diagnostic and Statistical Manual of Mental Disorders, 4th edition (DSM IV), a baseline score for depression of 10–30 and anxiety 8–26. 54 subjects completed the trial.	using any medications for at least one-month prior to starting the study; subjects with family or relationship problems as well as with significant deterioration in their general condition from baseline; signs of any other psychiatric disorder; unnormal studies for organic disease, including thyroid function test and complete blood count testing; any signs of substance misuse disorder, mental retardation, suicidal thoughts or attempt; pregnancy (confirmed by urine βhCG human chorionic gonadotrophin testing) and any grief reaction in the past 6 months.	12 week double-blind, placebo-controlled radnomised trial	saffron supplements significantly reduced of depression and anxiety scores (Beck Depression Inventory (BDI) and Beck Anxiety Inventory (BAI). *Crocus sativus* L. stigma appears to be an efficient agent in the treatment of mild-to-moderate anxiety and depression disorder with rare side effects shown.	too-small scale of the trial and single dose of saffron. The minor sample size and the temporary follow up is suggested in further survey.	[232]
Saffron capsule containing 30 mg of powder	25 mg of a dried saffron stigma extract (g. 1; *n* = 50) or 5 mg oral diazepam as a control (g. 2; *n* = 52). 11 subjects from control and 19 subjects from intervention group completed the trial.	40 patients suffering from MDD according to DSM-IV.	suicide risk, any medical or psychiatry disease; use of antidepressant or lipid-lowering drug in the past six months, pregnancy	4 week randomised double blind placebo controlled trial	Both group significantly improved the depression severity (Beck depression scale) without significant differences. No change in the lipid profile in both groups.	short duration of the study, a small population size due to the abandonment of study by some participants.	[346]
Saffron capsule (SaffroMood^®^) containing 15 mg of stigma extract and 1.65–1.75 mg crocin	a saffron capsule bis in day morning and evening (b.i.d.) (g. 1 or fluvoxamine capsule 100 mg/day (g. 2)	50 males and females, 18–60 age, with diagnosis of mild-to-moderate obsessive-compulsive disorder (OCD) according to DSM-V-TR and Yale Brown Obsession Compulsion Scale (Y-BOCS) scores from 12 to 21, the participants did not receive any psychiatric medications 6 weeks prior to the study	other mental disorder reported on in the DSM-IV axis I, alcohol or substance (other than nicotine and caffeine) dependence; medical illness including cardiac, hepatic, renal, and neurologic diseases; pregnancy, and breast-feeding	10 week randomised double-blind parallel group trial	similar effectiveness of stigma as the selective serotonin reuptake inhibitor (SSRI) fluvoxamine and well tolerance in treatment of mild-to-moderate OCD, patients assessed at baseline and at the 2nd, 4th, 6th, 8th, 10th week based on Yale-Brown Obsession Compulsion Scale and Adverse Event Checklist. In contrast to fluvoxamine, the patients tolerated the stigma very well and there were no side effects.	lack of a placebo group., a small sample size, and a short follow-up period	[373]
Saffron capsule containing 15 mg of dried stigma extract and 1.65–1.75 mg crocin	saffron capsule b.i.d. (g. 1) or citalopram 40 mg/day (g. 2); 60 participant completed the study: saffron group *n* = 30 Male/Female 11/19; citalopram group *n* = 30 Male/Female 15/15	66 patients with major depressive disorder (MDD) accompanied by anxious distress according with a score < 19 in the 17-item Hamilton Depression Rating Scale (HDRS) for mild-to-moderate depression and a score < 24 in the 14-item Hamilton Anxiety Rating Scale, with mild-to-moderate severity.	antidepressant medication during the previous month; electroconvulsive therapy during the last 2 months; other mental disorders from DSM-IV Axis I; alcohol dependence or substance dependence (except for nicotine); severe depression, suicidal ideation, administration of aspirin, anticoagulants or nonsteroidal anti-inflammatory drugs; pregnant or breastfeeding women, hypertension, hypothyroidism, renal failure	6 week multicenter double-blind controlled randomised trial with rigorous adjustment for baseline clinical variables	safe and satisfactory use of stigma, comparable to citalopram, in treatment of mild to moderate MDD with anxious distress.	lack of a placebo control trial arm; use of only a fixed dose in the stigma therapy; a small size of the studied population and a short follow-up period.	[337]
Saffron capsule	500 mg capsules containing 450 mg of saffron on a daily basis for 6 weeks in addition to sertraline (50 mg) (g. 1) or 500-mg capsule of starch with the same protocol that used for group 1 (g. 2—placebo).	40 patients, 18–55 years, with mild-to-moderate generalised anxiety disorder (GAD), diagnosed according to the DSM-V, based on the HAM-A scores of 18–24 (mild-to-moderate anxiety), who received sertraline	pregnancy and lactation; antipsychotic medications over a month prior to recruitment; drug abuse; other psychiatric diagnosis (bipolar disorder, schizophrenia, mood disorders).	6 week randomised double-blind placebo-controlled trial	beneficial effects of saffron as an add-on therapy to sertraline for GAD patients (based on total HAM-A score).	too small sample size and a slightly short duration in addition to ethical constraints impeding the assessment of the effects of saffron alone—without sertraline or any other prescribed medication on GAD.	[217]
Saffron capsule containing 15 mg of dried stigma extract and 1.65–1.75 mg crocin	saffron capsule (15 mg of dried extract b.i.d.) (g. 1) or placebo (g. 2) for 4 weeks	36 married male patients,18–45 years, with fluoxetine-stabilised MDD symptoms (40 mg/day for 6 weeks) who had subjective complaints of sexual impairment.	other psychiatric disorders; taking other psychotropic medications within 4 weeks of screening visit; substance abuse within 6 months of recruitment; other serious or life-limiting conditions	4 week randomised double-blind placebo-controlled trial.	Based on International Index of Erectile Function scale and HDRS saffron is a tolerable and efficacious treatment for fluoxetine-related erectile dysfunction	inability to detect significant difference in some domains (e.g., orgasmic function) which showed near-significant results due to relatively small sample size. Too short duration of the study made it impossible to generalise the findings to long-term outcomes.	[374]
Saffron capsule containing 15 mg of dried stigma extract and 1.65–1.75 mg crocin	saffron capsule (15 mg of dried extract b.i.d.) (g. 1) or placebo (g. 2) for 4 weeks	34 married women 18–45 years, with fluoexetine-stabilised MDD symptoms (40 mg/day for 6 weeks) who experienced subjective feelings of sexual dysfunction.	other DSM axis disorders; medical comorbidities that could underlie sexual symptoms; using otherpsychotropic agents within 1 month of recruitment; substance abuse within 6 months of recruitment; other serious or life-limiting disease; pregnancy and lactation	4 week randomised double-blind placebo-controlled trial.	based on the Female Sexual Function Index (FSFI) and HDRS saffron is recognised to be safe and effective agent in alleviation of some of fluoxetine-induced sexual problems including arousal, lubrication, and pain in women.	Short study duration limited the interpretation of the present study regarding long-term effects of saffron on sexual dysfunction. Different dosages of saffron should be investigated in future	[375]
Crocin tablets	crocin tablets (30 mg/day; 15 mg b.i.d) or equivalent dose of placebo contained starch and food coloring (g. 2). 33 participants comleted the study: crocin group *n* = 16; Male/Female 4/12); placebo group *n* = 17; Male/Female 7/10).	34 participants—volunteers with metabolic syndrome (MetS) according to the International Diabetes Federation (IDF) criteria	pregnancy, lactation, age under 18 or over 70 years, use of antidepressant drugs, under 10 points on the Beck Depression Inventory (BDI)- questionnaire, grief or unpleasant event during the previous 6 months, and a lack of compliancy in taking the pills regularly	8 week randomised double-blind controlled clinical trial	crocin at a dose of 30 mg per day for 8 weeks reduced the symptoms of depression in subjects with MetS compared to the control group, and this effect was independent of its effect on the serum serum pro-oxidant/anti-oxidant balance (PAB). Depressive symptoms were assessed using the BDI.	The participants of the study did not have had a clinical diagnosis of depression. The small sample size, the evaluation of the effects of only 1 dosage an the relatively short duration of the treatment and follow-up.	[296]
Curcumin in capsules contains curcuminoids 88%, volatile oils 7% from *Curcuma longa rhizomes*, saffran were standardised to contain > 3.5% of lepticrosalides including safranal and crocin	placebo-cellulose (g. 1), curcumin extract 250 mg b.i.d. (g. 2), curcumin extract 500 mg b.i.d. (g. 3), combined low-dose curcumin extract plus saffron stigma 15 mg b.i.d. (g. 4)	123 patients, 18–65 age, with MDD who met DSM-IV criteria, and had IDS-SR30 score ≥ 18	diabetes; suicide risk; chronic fatigue syndrome, fibromyalgia or asthma; hypertension; cardiovascular and autoimmune diseases; any infection or illness over the past month; psychotic, bipolar, comorbid obsessive-compulsive; posttraumatic stress, neurodegradative disorders; any substance abuse or dependence; treatment with antiplatelet or anticoagulant medications; pregnant or breastfeeding women	12 week randomised double-blind placebo controlled trial, with a 1 week placebo run-in phase	Different doses of curcumin and combined curcumin/saffron (stigma) treatments effectively reduced major depressive and anxiolytic symptoms based on IDS-SR30 and STAI, enhanced potency of curcumin in atypical depression compared to other depressed counterparts	investigations with larger sample sizes are required to examine the efficacy of the differing doses of curcumin and stigma/curcumin combination and to assess effects in atypical depression	[376]
saffron (stigma) aqueous extract; crocin	capsules of saffron aqueous extract (30 mg;) (group 1 *n* = 20; Male/Female 11/9), crocin (30 mg) (group 2; *n* = 19; Male/Female 9/10), or placebo (cornstarch-vehicle) (group 3; *n* = 19; Male/Female 8/11) for 8 weeks. One participant of group 2 and one of group 3 did not complete the study. Participants completed the demographic questionnaire, Beck depression inventory-II (BDI-II), Hurlbert index of sexual desire (HISD), and MacNew health-related quality of life questionnaire.	Fifty-eight, 40–65 year-old, Coronary Arthrety Disease (CAD) males and females patients. The subjects did not receive any psychotherapeutic or psychotropic drugs and only used common drugs for their cardiometabolic disorders. Hypertension was defined as systolic and/or diastolic blood pressure ≥ 150/90 mmHg or receiving antihypertensive medications.	autoimmune diseases, malignancies; insulin therapy; nursing profession, pregnancy; hypersensitivity to saffron; patients with heart attacks; antidepressants treatment;	8 week randomized double-blind, placebo controlled, clinical trial	saffron (stigma) aqueous extract and crocin to the similar extent severely decreased the BDI-II score but not markedly affected HISD scores. However, they could significantly improve the total quality of life and its subscales. After adjustments for age, sex, and diagnosis time, similar results were obtained. Therefore, saffron and its active constituent, crocin, could improve depression and health-related quality of life in patients with CAD, whereas they had no significant effects on sexual desire. Although these agents can be used as suitable adjunct agents in CAD patients, large-scale trials are justified.	Too small scale of trial. Considering the limited sample size, subgroup analysis based on sex was impossible. To obtain comprehensive results, it is recommended to conduct further research on both sexes using a large sample size. Moreover, to completely assess sexual life quality in patients, it is suggested to concurrently use several valid and reliable instruments.	[377]
Saffron capsule	saffron capsule SaffroMood^®^ (15 mg b.i.d.) (g. 1; *n* = 32) or fluoxetine capsule (20 mg b.i.d.)	64 women between 18–45 years of age, with mild to moderate postpartum depression based on the Diagnostic and Statistical Manual of Mental Disorders, Fourth Edition, Text Revision (DSM-IV-TR), who had Hamilton Depression Rating Scale (HDRS 17-item) score ≤ 18	psychotic depression, history of suicidal or infanticidal thoughts, a history of bipolar disorder, substance or alcohol dependence (except of nicotine), lactation, hypothyroidism and acute medical illness. Patients with any diagnosis other than postpartum depression on the DSM-IV-TR axis I	6 week randomised double-blind, clinical trial	No significant effect of the time×treatment interaction on the HDRS score between saffron and fluoxetine group. Therefore saffron is a safe alternative medication for improving depressive symptoms of postpartum depression	lack of a placebo group, a small number of participants and short period of follow-up	[341]
Saffron tablet containing 15 mg of stigma and 235 mg of lactose, magnesium stearate, and sodium starch glycolate	saffron tablet 15 mg b.i.d. (30 mg/day (g. 1) or equivalent dose of placebo (g. 2).	60 new breastfeeding mothers suffering from mild-to-moderate postpartum depression, over 18 age with BDI-II score of lower than 30; live-born infant delivery over the preceding nine months	significant medical illness (gestational diabetes, pre-eclampsia); pregnance; present or past history of drug or alcohol abuse, smoking, alcohol, or illicit drug use during pregnancy; treatment with any medications affecting mood; anticoagulants therapy; current psychotropic medication; ongoing need for medications known to cause depression or psychosis; kidney failure; a BDI-II score of ≥30; unstable medical condition that might interfere with completing the trial; any allergies to stigma in the mother or the infant; any significant deterioration in symptoms	8 week randomised double-blind placebo-controlled trial conducted in three healthcare centres	saffron stigma showed a more significant impact on the BDI-II than the placebo when administered to treat minor postpartum depression in breastfeeding mothers.	study accessible only to women that attended healthcare centre but not for all breastfeeding mothers; the questionnaire recorded the demographic information from mothers concerned about their infant’s vaccination	[70]
A combination of *C*, *sativus* stigma and *Rhodiola rosea* roots extracts	supplementation with one tablet contained 154 mg of *Rhodiola* roots and 15 mg of saffron exstracts, b.i.d.	45 adults (18–85 age) suffering from mild-to-moderate depression according to ICD10 and reaching a score of 8–18 on the HRSD	treatment with antidepressants, severe MDD defined by the ICD10 or HRSD score >18; suicidal attempt, treatment with piperine or St John’s Wort medications; chronic ilness: arterial hypertension, cardiac or renal insufficiency; psychiatric disorders: schizophrenia, bipolarity; addiction; pragnant and lactating women	6 week observational study conducted with general practitioners	rapid improvement of both depressive and anxiety symptoms assessed with HRSD, Hospital Anxiety and Depression Scale (HADS), Clinical Global Impresiion (CGI) scale, and Patient Global Impression of Change (PGIC) scale.	absence of a control groups, a double-blind placebo-controlled study is needed to confirm these results	[378]
Saffron capsule	capsule of saffron (30 mg/day) and fluoxetine (20 mg/day) (treatment group g. 1) or capsule of placebo and fluoxetine (20 mg/day) on a daily basis for 4 weeks (control group g. 2)., Fasting blood samples were collected before treatment and at the end of the study. For females, blood samples were collected on the third day of their menstrual cycle.	40 adult male and females, 18–55 years old, outpatients diagnosed with severe depression. Participants met the diagnostic and Statistical Manual of Mental Disorders, fourth edition (DSM-IV) for major depression based on the structured clinical interview for DSM-IV.	any antidepressant treatment during the last 6 months; a history or current idea of suicide; chronic diseases such as metabolic disease and cancer	4 week randomised double-blind parallel-group clinical trial	*C. sativus* and flouxetine co-treatment for 4 weeks, significantly improved mood in patients with severe depression. These clinical findings were accompanied by the improvements in the Beck Depression Rating Scale results, in the both groups without marked differences in terms of side effects. This study is the first clinical trial that showed both antidepressant effects and serum homocysteine decreasing activity of saffron. It may suggest the safe application of *C. sativus* as a complementary treatment for depression.	Authors do not provide limitations of the studies nor a recommendation for the future investigation.	[379]
Saffron capsule	saffron 15 mg (g.1) or duloxetine 30 mg (g. 2) starting with 1 capsule per day in the first week followed by 2 capsules per day from week 2 until the end of week 8.	54 participants, both sexes, aged 18–60 years diagnosed with fibromyalgia based on the American College of Rheumatology 2010 criteria with a pain score ≥ 40 based on visual analogue scale; 46 subjects completed trial.	rheumatologic diseases excluding fibromyalgia; inflammatory/infectious/autoimmune arthritis; comorbid neuropsychiatric disorders except depressive disorders based on the Diagnostic and Statistical Manual of Mental Disorders-IV-Text Revision (DSM-IV-TR); suicidal ideation; multiple sclerosis; pain due to traumatic injuries; drug history of duloxetine or saffron use; current use of psychoactive medications specially serotonergic compounds or monoamine oxidase inhibitors (MAO-Is); recent use of muscle relaxants, steroids, opioid analgesics, benzodiazepines, anti-epileptics, or injective analgesics; substance use disorder during the 2 years prior to the study; history of using thioridazine, acetylcholinesterase inhibitors, warfarin, or medications affecting the P450 CYP4A3 enzyme in the 2 weeks before the study; pregnancy, breast feeding, or women with no contraception history	8 week randomised double-blind parallel-group trial	comparable efficacy of saffron and duloxetine in the treatment of fibromyalgia symptoms based of the Hamilton Rating Scale for Depression, Hospital Anxiety and Depression Scale, Global Fatigue Index, Fibromyalgia Impact Questionnaire, and Brief Pain Inventory Saffron and duloxetine	lack of placebo group; small sample size of the study; using a low fixed dose of saffron for a short period of time (i.e., 6–8 weeks) provided insufficient information for long-term adverse effects of saffron compared to antidepressants due to budgetary and executive limitations; the various subgroups typology of fibromyalgia should be taken into account; aside from paraclinical evaluations that concerned safety issues, the biological markers of fibromyalgia that were affected by saffron’s mechanisms of action have not been assessed.	[380]
Saffron capsule of stigma extract containing 1.65–1.75 mg crocin	Saffron capsule containing stigma extract (30 mg/day, 15 mg twice daily) (g. 1.) or placebo (g. 2) for 6 weeks.	fifty-six post-menopausal women, over 40 years of age with no menstrual period in the last 12 months with a clinical diagnosis of hot fashes having a score ≥ 40 in Hot Flash-Related Daily Interference Scale (HFRDIS) with MDD based on the Diagnostic and Statistical Manual of Mental Disorders, Fourth Edition, Text Revision (DSM-IV-TR) criteria and mild-to-moderate depression based on a score of ≤22 in the 17-item HDRS. Patients with hot flash attacks ≥14 times per week for at least 2 months was included into studies. In case of positive history for oophorectomy, more than 6 weeks should have elapsed from surgery and the level of serum FSH should be equal or more than 40 U per mL.	diagnoses other than depression on the DSM-IV-TR axis I; ingestion of any psychotropic and antidepressant medications, any Selective Estrogen Receptor Modulator medications (e.g., tamoxifen and raloxifen), anyaAromatase inhibitor medications (e.g., anastrozole, letrozole, and exemestane), leuprolide acetate, clonidine, gabapentin, pregabalin, amino acid supplements, over the counter (OTC) medications that reduced hot fashes during the last 4 weeks, ingestion of estrogen and progesterone-based medications, history of suicidal thoughts, substance or alcohol dependence except of nicotine during the last 3 months and electroconvulsive therapy during the last 2 months.	6 week, multicenter, randomised, double-blind, parallel group clinical trial	safron is a safe and efective treatment in improving hot flashes and depressive symptoms in post-menopausal healthy women. Safron, with fewer side efects, may provide a non-hormonal and alternative herbal medicine option in treatment of women with hot flashes.	the small number of participants and the short period of follow-up. Further research with a longer study period, an active agent such as venlafaxine and a higher sample size to consider patients with different biological and racial backgrounds is needed.	[381]
affron^®^, a patented, obtained at industrial scale, standardized commercial saffron (stigma) extract containing ≥3.5% of total bioactive compounds safranal and crocin isomers	saffron extract (affron^®^, 22 or 28 mg/day) (g.1), or placebo (g.2). The active treatment was a TGA-listed coated tablet containing either 11 mg or 14 mg of standardised saffron extract (affron^®^), derived from the stigmas of *Crocus sativus* L. and standardised to contain >3.5% Lepticrosalides^®^ a measure of bioactive compounds present in saffron, including safranal and crocin. The placebo tablet contained the same excipients as the active one (microcrystalline cellulose and calcium hydrogen phosphate).	128 (75 male and 45 female), 18–77 year-old participants self-reporting low-mood not diagnosed with depression or another mood disorder recruited from the CRO’s subject database and the public media	patients with a mood disorder or tested positive for depression on the Beck Depression Inventory (BDI > 20); anticoagulation therapy; hypertension and antihypertensive medications treatment, severe renal and/or hepatic insufficiency; history of alcohol and/or drug abuse; current participation or participation in any other clinical trial during last 30 days; diagnosed mood disorder (MDD, bipolar or substance-induced disorder); positive test for moderate to severe depression on the BDI; insomnia or night-shift employment and other reasons that resulted in an inability to have a normal night’s sleep; severe pre-menstrual syndrome with mood or pain that would change during the study period; any neurological disorder e.g., multiple sclerosis; using supplements (nutrients, herbs) that would impact mood (St John’s Wort, Tryptophan, SAM-E, 5-hydroyxtryptophan, Melatonin, Gamma aminobutyric acid GABA); using saffron supplement	4 week randomised, double-blind, parallel, placebo controlled trial	significant decrease in negative mood and symptoms related to stress and anxiety at a 28 mg/day dose (with a significant difference between 28 mg/day and placebo on the primary outcome measure Total Mood Disturbance scale including POMS Tension, Depression, and Confusion subscales), but no treatment effect at the 22 mg/day dose. Therefore, the results demonstrated the effectiveness of affron^®^, on improving low mood, stress management and anxiety reduction without side effects in otherwise healthy participants, offering a natural alternative to standard treatments in long-term and prophylactic management, where appropriate, of low mood states. affron^®^ reduces risk of progressing to more severe and eventually clinical manifestations.	the self-reporting nature of both the screening and the testing, and the possibility of confounding variables. Limited generalisability of the study due to the testing of the healthly population and excluding the participants with a high BMI, severe PMS, insomnia, and those with a history of drug and alcohol abuse, i.e., the conditions very often associated with low mood.	[382]
affron^®^, a patented, obtained at industrial scale, standardised commercial saffron (stigma) extract containing ≥3.5% of total bioactive compounds safranal and crocin isomers	tablets containing placebo or stigma extract (14 mg b.i.d) standardised to contain >3.5% lepticrosalides^®^ as measure of bioactive compounds (safranal, crocin). Affron^®^ samples were obtained from Pharmactive Biotech Products SL. The placebo tablet contained the same excipients as the active one (microcrystalline cellulose and calcium hydrogen phosphate).	80 youths, 12–16 age, with mild-to-moderate anxiety or depressive symptoms	psychiatric disorders other than mild or moderate depression, anxiety disorder, suicidal thoughts, chronic diseases: cardiovascular disease, brain disorders, seizures, diabetes, learning disabilities, addiction	8 week randomised double-blind placebo-controlled trial	improvement of anxiety and depressive symptoms in teenagers with mild-to-moderate symptoms after stigma administration from the perspective of the adolescents. Youth in self-reports declared greater improvements in overall internalising, separation anxiety, social phobia and depression, however, these beneficial effects were not corroborated by their parents.	self-reporting nature of both the screening and testing, limited duration of the study, single treatment dose	[234]
affron^®^, a patented, obtained at industrial scale, standardised commercial saffron (stigma) extract containing ≥3.5% of total bioactive compounds safranal and crocin isomers	tablets containing placebo or stigma extract (14 mg b.i.d) standardised to contain >3.5% lepticrosalides^®^ as measure of bioactive compounds (safranal, crocin). Affron^®^ samples were obtained from Pharmactive Biotech Products SL. The placebo tablet contained the same excipients as the active (microcrystalline cellulose and calcium hydrogen phosphate).	physically healthy volunteers both of sex aged 18–65 years, with persistent depression, currently (at least eight weeks) treated with a stable dose of single pharmaceutical antidepressant who continued to suffer from mild-to-moderate depressive symptoms as assessed by a score greater than six on the Montgomery–Åsberg Depression Rating Scale (MADRS) (nine-items). Of the 160 participants enrolled, 139 provided usable data.	current or 12 month history of any psychiatric disorder other than mild-to-moderate depression or anxiety; in self-harm behaviours and/or serious suicidal ideation treatment with any pharmaceutical medication, apart from a single pharmaceutical antidepressant, oral contraceptives and the occasional use (no more than fortnightly) of analgesics (e.g., ibuprofen, paracetamol); currently taking saffron or other herbal supplements; a current or history of a clinically significant, chronic medical condition including cardiovascular disease, organic brain disorder, seizure, diabetes, severe obesity, or use of illicit drugs; pregnant or breastfeeding women, women intending to fall pregnant; subjects reporting a greater than ten-year continuous use of antidepressant medication with no remission in depressive symptoms greater than six months over this period. Eligibility was initially assessed via the completion of a questionnaire that screened for current medication use, suicidal ideation, self-harm behaviours, history of medical/psychiatric disorders, alcohol, nicotine and other drug use, supplement and vitamin intake, and pregnancy/breastfeeding status.	8 week randomised double-blind placebo-controlled trial	Based on the clinician-rated Montgomery–Åsberg Depression Rating Scale (MADRS) affron^®^ markedly reduced depressive symtoms compared to placebo with decreases of 41 and 21%, respectively, but the decrease in the scores of self-rated MADRS was comparable in both of experimental groups. As it was assessed with Antidepressant Side-Effect Checklist (ASEC) and Short Form-36 Health Survey (SF-36), saffron was associated with a greater reduction in adverse effects of antidepressants, although this was non-significant after covarying for baseline values. Quality of life improved in both groups with no significant between-group differences. Due to the conflicting results, further research is needed to clarify the clinical benefits of saffron as an adjunctive treatment for adults with persistent depressive symptoms despite antidepressant drug treatment. The efficacy of adjunctive saffron use on specific antidepressant types and classes should be investigated.	The longer duration period of the trial and dose-escalation studies for treatment non-responders will be useful to examine the efficacy and safety of higher than studied affron^®^ dose. Support for the saffron antidepressant efficacy should be demonstrated via clinician- rather than self-administered assessment. In order to clarify the saffron antidepressant mechanisms the objective measures of change including changes in cortisol, neurotrophins and inflammatory and oxidative stress markers or changes in neurological activity through the measurement of EEG activity and cognitive testing may be helpful. Recruitment of the participants solely via social media promotion might have skewed the examined population. Multi-measure approaches i.e., diaries, questionnaires and pharmacokinetic measurements, not only participant self-reporting of remaining tablet numbers, should be used for adherence to pill intake.	[383]
Crocin	15 mg/day of crocin (group 1; *n* = 26) or placebo (gropup 2; *n* = 27) twice a day for 8 weeks	53 patients, aged 18–60 years, currently undergoing a methadone maintenance treatment (MMT) and opioid dependence in the past year, evaluated by the drug abuse section of the Structured Clinical Interview for DSM-IV, Beck Depression Scores > 20 and Beck Anxiety > 15, seeking for treatment.	taking crocin and anti-inflammatory and antioxidant supplements during the last 3 months before the intervention and history of metabolic diseases including diabetes, hypertension, thyroid, and cardiovascular disease.	8 week randomised double-blind, parallel, placebo-controlled trial	Crocin severely decreased Beck Depression Inventory score and Beck Anxiety Inventory score. It also reduced fasting glucose, insulin levels and resistance, triglycerides, very low-density lipoprotein as well as total cholesterol levels, but markedly increased insulin sensitivity. Crocin intake was associated with a reduction in high-sensitivity C-reactive protein and MDA, as well as a rise in total antioxidant capacity levels. Administration of crocin supplements to patients undergoing an MMT program had ameliorating effects on mental health scales, and improved their metabolic, and genetic parameters.	Too short-term of an intervention; further studies focused on the cognitive functions, craving, and withdrawal syndrome in subjects under a methadone maintenance treatment (MMT) program are necessary; the effects of crocin administration on urinary or/and serum crocin should be evaluated.	[384]
affron^®^, a patented, obtained at industrial scale, standardised commercial saffron (stigma) extract containing ≥3.5% of total bioactive compounds safranal and crocin isomers	tablets containing placebo or stigma extract (14 mg b.i.d) standardised to contain >3.5% lepticrosalides^®^ as measure of bioactive compounds (safranal, crocin). Saffron samples were obtained from Pharmactive Biotech Products SL. The placebo tablet contained the same excipients as the active tablet (microcrystalline cellulose and calcium hydrogen phosphate).	Eighty-six, 40–60 year-old, perimenopausal women experiencing menopausal complaints with a total score of greater than 16 on the Greene Climacteric Scale (GCS), an intact uterus and ovaries and a body mass index (BMI) between 18 and 35 kg/m^2^; patients were medication-free for at least 3 months (apart from the contraceptive pill and/or once weekly use of analgesics) and had no plan to commence new treatments over the study period. A total of 39 subjects from saffron group and 37 subjects from placebo group completed the trial.	smokers; consumption more than 14 standard drinks of alcohol per week; current or illicit drug abuse within the last 12 months; suffering from medical conditions including but not limited to: diabetes, hyper/hypotension, cardiovascular disease, a gastrointestinal disease requiring regular use of medications, gallbladder disease/gallstones/biliary disease, endocrine disease, psychiatric disorder (excluding mild-to-moderate anxiety), or neurological disease (Parkinson’s or Alzheimer’s disease, intracranial haemorrhage, head or brain injury); women who had any significant surgeries over the last year, or women taking saffron or other supplements that may affect menopausal symptoms.	two-arm, parallel-group, 12 week, randomised, double-blind, placebo-controlled trial	Affron^®^ markedly improved psychological symptoms in perimenopausal women reducing depressive and anxiety sydromes. Data from the Greene Climacteric Scale (GCS) revealed a significantly greater reduction in the GCS psychological score, characterised by a 33% reduction in anxiety and a 32% reduction in depression scores. Saffron to a greater extent than placebo reduced the PANAS negative affect score (Positive and Negative Affect Schedule). However, vasomotor or other somatic symptoms within intervention and control groups were not markedly different. Given the positive, mood-enhancing findings, further investigations into the benefits of various saffron doses in more clearly-defined populations, presenting with different specific menopausal complaints and using validated self-reported, clinician-administered anxiety and depression, and biological outcome measures, will be important. Changes in sex hormones, and other pertinent markers associated with inflammation, oxidative stress, HPA-axis activity, and neurotrophic activity should be assessed.	As no formal medical assessment comprising an evaluation of hormone concentrations and a comprehensive examination of confounding medical, lifestyle, and dietary factors was undertaken some women in other reproductive stages might have been recruited in this study. The effects of saffron in women with a formally diagnosed depression or anxiety-related disorder, and with varying levels of severity should be studied. The co-administration of saffron with pharmacological antidepressants in perimenopausal women with more severe specific climacteric symptoms currently taking antidepressants and/or on hormone replacement therapy should be evaluated. The efficacy and safety of different saffron extracts at various doses and treatment durations should be examined.	[385].
Saffron capsule	two 15 g saffron (group 1) or placebo (group 2—control) capsules daily for 8 weeks. A total of 29 subjects in the saffron and 28 in the placebo group completed the trial.	Sixty-two methamphetamine abusers (mean age 33 years) with Human immunodeficiency virus infection and acquired immunodeficiency syndrome (HIV/AIDS)	Psychosis; mania and hypomania period; diseases related to depression such as diabetes, multiple sclerosis, etc; simultaneous psychotherapy or pharmacotherapy; drug and alcohol abuse	8 week randomised double-blind placebo-controlled trial	Saffron and its ingredients had been effective in reducing depression assessed with using self-report Beck depression inventory (BDI-II). Saffron with its active ingredients (crocin and safranal) by serotonin and dopamine secretion in the brain help in reducing depression among recovered consumers of methamphetamine living with HIV/AIDS.	the duration of intervention, sample size and a tested saffron extract dose was similar to other studies that might aim to find a dose–response effect. Further studies with different doses of saffron extract, different durations of intervention and larger sample sizes are required.	[386]
Saffron capsule	saffron capsule contained 15 mg stigma hydroalcohilic extract b.i.d. (g.1) or placebo b.i.d. (g. 2).	54 adults, 40–65 age, type 2 diabetic outpatients (fasting plasma glucose levels of ≥126 mg/dL) suffering from mild to moderate comorbid depression–anxiety (CDA) diagnosed by using the Diagnostic and Statistical Manual of Mental Disorders-IV (DSM-IV).	treatment with anti-depressant and anti-anxiety medications or insulin; severe depression and anxiety as well as recent severe stress or emotional defeat; addicted smokers; any diseases other than depression and anxiety or mental disorders in the first degree relatives; patients with uncontrolled bood glucose (fasting blood sugar test FBS > 170 mg/dL); high physical activity; recent hospitalisation; pregnant or lactacting women and those who had planned for pregnancy.	8 week randomised double-blind placebo-controlled trial	saffron relieved symptoms of mild-to-moderate CDA in in type 2 diabetic patients without side effects. Based on Hamilton Depression and anxiety measurements (Beck Depression Inventory II (BDI-II, 21-item) and Beck Anxiety Inventory (BAI, 21-item)), the Pittsburgh Sleep Quality Index (PSQI), and the Satisfaction with Life Scale (SWLS) assessment, after the intervention, mild-to-moderate CDA, anxiety and sleep disturbance, but not depression alone, were relieved markedly in the saffron group, while, the changes were not significant in the placebo group. Dietary intake, physical activity, life satisfaction parameters, anthropometric measures and blood pressure parameters of the patients within each of both treatment groups did not change markedly during the intervention. Saffron may be suggested as an alternative treatment for CDA in diabetic patients.	the duration of intervention, sample size as well as a tested saffron extract dose was similar to other studies that might confine finding a dose–response effect.	[387]
Saffron capsule	saffron capsule contained 15 mg stigma hydroalcohilic extract b.i.d. (g. 1) or placebo b.i.d. (g. 2)	40 men and women, over 70 years of age, with on-pump coronary artery bypass grafting (CABG), who had a Wechsler Memory Scale score > 70	previous treatment with saffron stigma or acetylcholinesterase inhibitors, hypersensitivity to herbal compounds, comorbid neuropsychiatric disorders, and serious medical conditions other than cardiovascular diseases.	12 week randomised double-blind placebo-controlled trial	there were no significant difference between two groups and time × treatment interaction effect based on Wechsler Memory Scale as well as Mini Mental Status Examination and subscales of Hospital Anxiety and Depression Scale. Therefore, there were no benefits of saffron in treatment of CABG-related neuropsychiatric conditions.	too small sample size and too short duration of intervention	[388]
Saffron capsule	Capsule of saffron (60 mg/day) (g. 1.) or sertraline (100 mg/day) (g. 2).	50 out-patients aged older than 60 years (mean age = 65 years; 70% males) with diagnosed MDD based on the DSM 5 criteria; HDRS seven or higher	Acute suicidality; other serious psychiatric disorders i.e., bipolar disorders, substance use disorder, anxiety disorders, veterans with posttraumatic stress disorder (PTSD); Intake of antidepressants during the last 4 weeks; intake of aspirin, anti-coagulant drugs or non-steroidal anti-inflammatory drugs (NSAID); undergoing other treatments for MDD such as psychotherapy, neuromodulation, regular, supervised physical activity trainings, or specific nutritional regimen; other somatic complaints such as diabetes, known and severe sleep issues such as obstructive sleep apnea, restless legs syndrome or insomnia, as referred from the patient and from medical records; possible adverse effect of the study.	6 week double-blind, randomised, sertraline controlled intervention study	Symptoms of depression (HDRS assessment) decreased over time (Timepoints: baseline, week 2, 4, and 6), with no advantages or disadvantages for the saffron or sertraline condition. Saffron appears to be a natural powerful antidepressant for older people, who might be more reluctant to the use of synthetic medications.	medication adherence was not systematically assessed. Latent and unassessed dimensions, i.e., sleep quality, quality of social support, along with nutritional factors such as the intake of omega-3-polyunsaturated fatty acids, might have biased two or more dimensions in the opposite directions. The quality of the data does not explain why saffron had a favourable effect on symptoms of depression. Longer-term effect of saffron, sertraline, or both on depression should be investigated.	[343]
Crocin	30 mg/day crocin (2 plus crocin tablet, 15 mg BID) (*n* = 25) (group 1) or placebo (2 tablets per day, 15 mg BID) (*n* = 25) (group 2.), one hour after taking food, for 8 weeks.	50 patients–volunteers, aged 18–60 years under methadone maintenance treatment; participants had confirmed diagnosis of substance dependency based on DSM-IV.	taking crocin, multivitamin–mineral and antioxidant supplements during the last 3 months before the intervention initiation; history of metabolic diseases including diabetes, hypertension, thyroid and cardiovascular disease	8 week randomised, double-blinded, placebo-controlled trial	Crocin administration to the patients during methadone maintenance treatment severely reduced depression and anxiety symtoms (BDI, BAI) as well as improved general health questionnaire scores, sleep quality (standardised sleep questionnaire Pittsburgh Sleep Quality Index) and sexual functions (International Index of Erectile Functions). Crocin can be recommended as an effective adjunct to methadone in opioid withdrawal protocols because of the ability to improve the quality of life and diminish opioid side effects in patients during methadone maintenance treatment.	too short duration of intervention; the pain in methadone-treated patients as well as the effects of crocin on biomarkers of inflammation, oxidative stress, and its related gene expression should be evaluated.	[389]
Saffron capsule	saffron capsule contained 15 mg stigma hydroalcohilic extract b.i.d. (g. 1) or placebo b.i.d. (g. 2). The demographic and clinical variables at baseline were the same in the two groups.	73 adult overweight women with BMI ≥ 25 comorbid with mild-to-moderate depression. Depression was diagnosed with a semi-structured clinical interview based on SADS (Schedule for Affective Disorders and Schizophrenia) and the Beck Depression Inventory-II (BDI-II) designed to measure severity of depression consistent with symptoms of depression as presented in the DSM-V. The case score of BDI-II 14–28 was included into the studies. A total of 52 patients finished the study.	severe depression—identified by a score of 29 or higher in BDI-II; other severe psychiatric disorders (bipolar mood disorder and schizophrenia, and those who had suicidal thoughts); taking antidepressants and other medications affecting the appetite, and bodyweight; pregnant, lactating and postmenopausal women; subjects with hypothyroidism and athletes.	12 week randomised double-blind placebo-controlled trial	saffron capsules were not effective in reducing food cravings or bodyweight, but as a safe over-the-counter supplement, it may help reduce the symptoms of depression in overweight patients who experience mild or moderate depression. Mean depression scores in the saffron group significantly decreased compared to placebo.	the study was performed in a weight reduction clinic, so the cases may have had minor comorbidities, such as some types of eating disorders, but there were no cases of bulimia/purging behaviour. It was impossible to exclude all comorbidities but such covariates have been controlled with repeated-measures ANOVA. Chronic nature of depression and obesity imposes the need for long-term studies before firm conclusions can be made regarding saffron’s efficacy and safety.	[390]
Saffron capsule	capsule of saffron 30 mg/day (15 mg twice a day; morning and evening; group 1; *n* = 82) or capsule fluoxetine (20 mg twice a day; group 2; *n* = 82) for two menstrual cycles (cycles 3 and 4). The data were collected in two stages through a self-designed questionnaire (on day 5 of menstrual cycle) and validated questionnaires of Prospective Record of the Impact and Severity of Menstruation and Hamilton Depression Rating Scale at the end of the period.	164, 20–45 years working women with the premenstrual syndrome	major physical or psychiatric disorder or substance abuse in the previous 6 months	2 month double-blind, randomised Clinical Trial Study	Saffron to a similar extent as fluoxetine reduced the premenstrual syndrome symptoms such as abdominal bloating, depression, and mood swing, and could even better relieve the breast and abdominal pain than fluoxetine. Therefore saffron could be effective in reducing the symptoms and cause fewer side effects than chemical drugs.	using only a fixed dose of saffron and short period of follow-up.	[391]
Saffron extract	30 mg saffron extract (Safr’InsideTM) standardised in SafromotivinesTM (a blend of more than 25 active compounds, including safranal >0.2% (HPLC)) (group 1) or a placebo group (control) for 8 weeks. Saffron capsule contained 15 mg saffron extract plus 345 mg maltodextrin. Placebo capsule contained 350 mg maltrodextrin. 26 subjects in placebo and 30 in saffron group completed the trial. Acute and chronic effects of saffron extract and placebo were assessed before, during, and after a laboratory stressor, the Observed Multitasking Stressor (OMS) on days 1, 14, 28, and 56 after treatment consumption.	Seventy-three male and female subjects aged 18–60 years who self-reported feelings of anxiety and/or stress and low mood in their daily lives; subjects had total score ≥ 40 on the Profile of Mood States 2 (POMS), <16 on the GAD-7 questionnaire, and ≤10 on the PHQ-9 questionnaire.	any psychological pathology (e.g., depression, generalised anxiety disorder) within the previous 3 years; body mass index (BMI) ≤ 18.5 or ≥30 kg/m^2^; uncorrected visual impairment; food allergies/insensitivities; an hormonal status likely to induce an unstable/fluctuating emotional state (e.g., menopausal transition); presence of life event likely to induce unstable/fluctuating emotional state (e.g., change of professional function/situation, death of a family member, divorce, surgery); high blood pressure (systolic over 159 mm Hg or diastolic over 99 mm Hg); pregnant women, seeking to become pregnant, or lactating; worked night shifts; high levels of physical activity (to avoid bias in the salivary cortisol measures); consumed >500 mg caffeine per day; current smokers; dietary supplement use 2 weeks before enrolment.	8 week randomised, placebo-controlled, double-blind, parallel groups	saffron extract reduced depressive mood in healthy individuals experiencing subclinical mood disturbance. The beneficial effect of saffron on heart rate variability in response to a psychosocial stressor suggests that this natural extract may be particularly relevant for increasing resilience against the development of stress-related psychiatric disorders. Further studies are required to identify the exact mechanisms underpinning these effects in humans. Chronic effects of saffron on subjective anxiety, stress, and depressive feelings were assessed using a questionnaire battery (including Profile of Mood State-2, (POMS)) and acute effects in response to a lab-based psychosocial stressor were measured through psychological and physiological parameters.	the OMS, evidenced by the pattern of response in the cortisol measure, masked an effect of treatment on anxiety measures or indeed any of the other subjective measures; laboratory-based stressors have inherent limitations in eliciting robust psychobiological stress responses that mimic those experienced in real-world situations- especially with repeated measurements, which have been discussed elsewhere; identifying potential participants with subclinical mood disturbance resulted in a comparatively small number of individuals with a very specific response pattern to the screening questionnaires being enrolled to the trial.	[392]
Saffron tablets (pure head powder of natural saffron compressed and without any additives)	resistance training (RT)+ saffron supplementation (150 mg pill of pure saffron; *n* = 15) (g. 1) or a RT+ placebo (dextrose pill; *n* = 15) (g. 2). pure saffron or placebo was administered immediately after each RT session and at the same time on non-training days for 6 weeks.	Untrained young healthy males (age 24 years; stature 176.4 cm) who do not regularly exercise (<hour/week) and had no prior resistance training (RT) protocol experience. The RT program consisted of 24 RT sessions (6 weeks, 4 x/week). The exercises included leg presses, leg curls, bench presses, lat pulldowns, bicep curls, and triceps pushdowns	allergy/sensitivity to saffron; any medical issues such as diseases, diabetes, sleep disorders, or other risk factors; taking dietary supplements, medications, consuming alcohol, or smoking during the year prior to enrollment in the study; subjects unwilling to undertake the nutritional or RT protocol; participatedin exercise other than the prescribed RT program during the investigation; participants consumed any dietary supplements; missed more than one RT session or post-RT saffron supplementation during the study period. Above-mentioned criteria were evaluated using the Physical Activity Readiness-Questionnaire (PAR-Q) and the medical health/history questionnaire.	6 week randomised, double-blind placebo-controlled parallel trial	saffron combined with RT improved the concentration of blood markers implicated in depression (Anandamide, 2-Arachidonoylglycerol, dopamine, β-endorphin, and serotonin). Noth groups significantly increased muscular endurance with greater changes in the saffron-supplemented group. The addition of saffron supplement to chronic RT results in greater increases in levels of happiness (assessed via questionnaire “In general, do you feel happy?”) than RT alone. Furhter studies should evaluate the effects of different dosages of saffron supplementation in combination with RT in other populations, especially those with depression and low levels of happiness.	Body composition was evaluated via bioelectrical impedance, which is not as accurate as dual-energy X-ray absorptiometry (the gold standard technique for body composition measurements); lack of the measurements of the gene expression of the studied markers; as participants were previously untrained young males the generalisation of our findings to other cohorts should be avoided; since the RT program consisted of both upper and lower body exercises, the benefit of the RT intervention on muscular endurance might not be fully reflected by the the push-up specific test (an upper-body activity). Assessing lower body muscular endurance is required.	[393]
Crocin extracted and crystallised from saffron stogmas	30 mg/day of crocin tablets or placebo during chemotherapy	Seventy-two newly diagnosed women with non-metastatic Her2/neu-positive or triple-negative invasive breast cancer.	Pregnancy or breastfeeding; treatment with antidepressants or anti-anxiety drugs (during study or within past 6 months); taking hormone replacement treatment, sleeping pills or beta-blockers, and warfarin, luminal A or B, other pathologies rather than carcinoma of breast; non-invasive breast carcinoma in the absence of invasive components; history of hypersensitivity reactions to saffron	patients received crocin for 4 months during 4–6 months course of chemotherapy; depression and anxiety were assessed at baseline and the end of the trial (2 weeks after the last course of chemotherapy); patients were followed up for one year to assess the survival; block randomised, double-blind, parallel-group placebo controlled trial	Crocin administration during chemotherapy of breast cancer significantly ameliorated anxiety and depression (improved Beck’s Depression and Anxiety Inventories). Crocin coadministration affected the chemotherapy side effects assessed with Eastern Cooperative Oncology Group Common Toxicity Criteria leading to significant increase of leukopenia as well as decrease of hypersensitivity reaction and neurological motor dysfunction, but the frequencies of most side effects were equal in both groups. In addition, a trend toward survival improvement was observed, which is going to be investigated on longer follow up.	relatively short follow-up limited the extrapolation regarding the long-term effects of crocin on survival, anxiety and depression. Crocin was only administered during chemotherapy, and the effects of long-term use after chemotherapy are unclear. Further similar extrapolations enrolling patients with various sub-types of breast cancer (luminal cancer) and other malignancies are necessary. Poor compliance of some patients, small sample size, self-reported assessments of side effects, and also some confounding factors such as ethnicity or genetic diversity that their effects cannot be ruled out. Did not assess some confounding factors i.e., the level of cognitive function, pain recognition, and patients’ diets.	[394]
Saffron capsule	capsule (at 2:00 pm) before a meal containing 100 mg saffron powder (group 1.) or placebo (starch) (group 2.) for 8 weeks. A total of 30 subjects of each group completed the trial.	Seventy, 30–60 year-old overweight/obese patients with body mass index (BMI) between 25–35 kg/m^2^ type 2 diabetes (T2D) for at least 6 months diagnosed with fasting plasma glucose test FPG ≥126 mg/dL	insulin treatment, hormone replacement therapy and consuming dietary or antioxidant supplements in the previous 2 months; history of surgery or serious illness; pregnancy or lactation; smoking or alcohol intake. Anti-hyperlipidaemic and hypoglycaemic medications such as metformin were permitted over the study period.	8 week double-blind, randomised, placebo-controlled clinical trial	saffron notably reduced hyperglycaemia and hyperlipidaemia as well as improved liver function in T2D patients. Saffron also significantly improved depression (Beck depression inventory-II BDI-II), sleep quality and overall quality of life in diabetic patients. However, further long-term studies with each component of saffron are required to investigate the underlying mechanisms and to suggest saffron as an effective complementary and alternative therapy in type 2 diabetes	relatively short duration and the fixed-dose design of the study that did not allow to investigate dose-dependent effects. Some dietary factors (vitamin D, selenium, magnesium and chromium), which might act as confounders, were not evaluated.	[395]
Krocina™—a herbal medicine made of crocin	Krocina™ (15 mg) or placebo pill twice a day for 6 weeks	seventy-two, 18–64 years old opioid-dependent male patients passing the detoxification course and negative urinary test, who were referred to the Center for Substance Abuse Treatment and Addictive Behaviours (Soroush).	mood disorder; acute psychiatric disorders; the incidence of psychosis symptoms	6 week double-blind randomised parallel clinical trial	Krocina™ pills were ineffective in the decreasing substance users’ withdrawal syndrome, craving, depression, anxiety and stress in the detoxification period and abstinence phase. Similar findings were confirmed in patients with the negative urinary test only. Some psychological treatment protocols such as motivational interviewing sessions that routinely provided for subjects in the detoxification period are needed.	Low sample size and short duration of therapy	[396]

Explanations: b.i.d.—bis in day morning and evening; BDI—Beck depression inventory; BMI—body mass index; CDA—comorbid depression-anxiety; g.—group; DSM—The Diagnostic and Statistical Manual of Mental Disorders; HRSD or HDRS—Hamilton Rating Scale for Depression or Hamilton Depression; ICD10—International Statistical Classification of Diseases and Related Health Problems 10th Revision definition; IDS-SR30 score—Inventory of Depressive Symptomatology self-rated version; MDD—major depressive disorder; OCD—obsessive-compulsive disorder; STAI—Spielberger State Trait Anxiety Inventory.

**Table 10:** Results of clinical studies on the antidepressant effect of bioactive chemical compounds contained in *C. sativus* L. tepals and stigmas administered to patients as capsules and as a combination with saffron capsules.

Extract/Biologically Active Chemical Compounds	Treatment Groups (g.)	Participants	Exclusion Criteria	Duration of Study	Main Results	Limitations of Studies	Reference
Petals							
Capsule containing dried ethanolic extract of petals (15 mg).	a capsule of petals ethanolic extract 30 mg/day (g. 1; *n* = 20; Male/Female 11/9) or a placebo (g. 2; Male/Female 12/8). A total of 17 subjects form the control and 19 from the treatment group completed the trial.	40 adults (18–55 age) with major depression, and had a baseline Hamilton Depression Rating Scale (HRSD) score of at least 18; patients with mild-to-moderate depression	current cognitive disorder over the past year, disease bipolar and schizophrenia, treatment with allpsychotropic medications for at least 4 weeks before the study entry, risk of suicide, pregnant women or women not using methods of birth control	6 week double-blind randomised placebo-controlled trial	efficacy of petals in treatment of mild-to-moderate depression together with the profile of tolerable side-effects	follow up should be considered so further research in this area is needed	[311]
Each capsule had dried etanolic extract of petal (15 mg), and 0.30–0.35 mg safranal	a capsule of petal extract 15 mg b.i.d. (g. 1; *n* = 20; Male/Female 10/10) or fluoxetine 10 mg b.i.d. (g. 2; *n* = 20; Male/Female 9/11).	40 adults, 18–55 in age, who met the DSM-IV criteria for major depression based on a structured clinical interview for DSM and had a baseline HRSD score of at least 18 and ≤25	any clinically significant deterioration in the condition of the subject from baseline; suicide risk, pregnant women or women not using medically accepted means of birth control	a pilot 8 week double-blind randomised trial	Similar efficacy and safety of petal extract and fluoxetine in treatment of mild-to-moderate depression with a comparable remission rate of 25%	lack of a placebo group, using only a fixed dose, the small number of participants, short period	[339]
petals and stigmas							
Capsule of petal or stigma extract, each capsule had the dried ethanolic extract of petals or stigmas (15 mg), and contained 0.3–0.35 mg of safranal.	a capsule of petals (g. 1; *n* = 20) or stigmas (g. 2) 15 mg b.i.d.	40 adults, 18–55 in age, outpatients who met the DSM criteria for major depression based on a structured clinical interview for DSM and had a baseline HRSD score of at least 18 and ≤25.	current cognitive disorder over the last year, bipolar, schizophrenia, and border line personality disorder, treatment with any psychotropic medications for at least 4 weeks before study entry, risk of suicide	6 week double-blind randomised trial	efficacy and safety of petal and stigma to a similar extent in treatment of mild-to-moderate depression with a similar reemission rate of 18%.	too small-scale of a trial.	[397]

Explanations: b.i.d.—bis in day morning and evening; DSM—Diagnostic and Statistical Manual of Mental Disorders, HRSD or HDRS—Hamilton Rating Scale for Depression or Hamilton Depression Rating Scale; g.—group.

## Data Availability

Not applicable.

## References

[1] Fried E.I., Nesse R.M. (2015). Depression is not a consistent syndrome: An investigation of unique symptom patterns in the STAR* D study. J. Affect. Disord..

[2] Fried E.I., Nesse R.M. (2015). Depression sum-scores don’t add up: Why analyzing specific depression symptoms is essential. BMC Med..

[3] Sadock B.J., Sadock V.A., Ruiz P., Sadock B.J. (2015). Mood disorders. Kaplan and Sadock’s Synopsis of Psychiatry. Behavioral Sciences/Clinical Psychiatry.

[4] Otte C., Gold S.M., Penninx B.W., Pariante C.M., Etkin A., Fava M., Mohr D.C., Schatzberg A.F. (2016). Major depressive disorder. Nat. Rev. Dis. Primers.

[5] Thomas E., Seedat S. (2018). The diagnosis and management of depression in the era of the DSM-5. S. Afr. Fam. Pract..

[6] Clack S., Ward T. (2019). The classification and explanation of depression. Behav. Chang..

[7] Giannelli F.R. (2020). Major depressive disorder. JAAPA.

[8] Nussbaum A.M. (2020). Questionable agreement: The experience of depression and DSM-5 major depressive disorder criteria. J. Med. Philos..

[9] Lindhardt A., Hippe E. (2007). Psykiske sydgomme. Almen Praksis.

[10] Huarcaya-Victoria J., Bojórquez-De la Torre J., De la Cruz-Oré J. (2020). Factor structure of cotard’s syndrome: Systematic review of case reports. Rev. Colomb. Psiquiatr..

[11] Makara-Studzińska M., Rolla-Szczepańska R., Urbańska A., Nowakowska-Domagała K., Stecz P. (2017). Anxiety and depression in patients infected with *Borrelia burgdorferi*. Eur. J. Psychiatry.

[12] Tran B.X., Ho R., Ho C.S., Latkin C.A., Phan H.T., Ha G.H., Vu G.T., Ying J., Zhang M.W. (2019). Depression among patients with HIV/AIDS: Research development and effective interventions (GAPRESEARCH). Int. J. Environ. Res. Public Health..

[13] Zhu Q.Y., Huang D.S., Lv J.D., Guan P., Bai X.H. (2019). Prevalence of perinatal depression among HIV-positive women: A systematic review and meta-analysis. BMC Psychiatry.

[14] Banasiewicz J., Zaręba K., Bińkowska M., Rozenek H., Wójtowicz S., Jakiel G. (2020). Perinatal predictors of postpartum depression: Results of a retrospective comparative study. J. Clin. Med..

[15] Fiske A., Wetherell J.L., Gatz M. (2009). Depression in older adults. Annu. Rev. Clin. Psychol..

[16] Huang R., Yang D., Lei B., Yan C., Tian Y., Huang X., Lei J. (2020). The short-and long-term effectiveness of mother–infant psychotherapy on postpartum depression: A systematic review and meta-analysis. J. Affect. Disord..

[17] Kong X., Zheng K., Tang M., Kong F., Zhou J., Diao L., Wu S., Jiao P., Su T., Dong Y. (2020). Prevalence and factors associated with depression and anxiety of hospitalized patients with COVID-19. MedRxiv.

[18] Rao W.W., Zhu X.M., Zong Q.Q., Zhang Q., Hall B.J., Ungvari G.S., Xiang Y.T. (2020). Prevalence of prenatal and postpartum depression in fathers: A comprehensive meta-analysis of observational surveys. J. Affect. Disord..

[19] Serfaty M., King M., Nazareth I., Moorey S., Aspden T., Mannix K., Jones L. (2020). Effectiveness of cognitive–behavioural therapy for depression in advanced cancer: CanTalk randomised controlled trial. Br. J. Psychiatry.

[20] Zaręba K., Banasiewicz J., Rozenek H., Wójtowicz S., Jakiel G. (2020). Peripartum predictors of the risk of postpartum depressive disorder: Results of a case-control study. Int. J. Environ. Res. Public Health.

[21] Cooley C., Park Y., Ajilore O., Leow A., Nyenhuis S.M. (2022). Impact of interventions targeting anxiety and depression in adults with asthma. J. Asthma.

[22] Santomauro D.F., Mantilla Herrera A.M., Shadid J., Zheng P., Ashbaugh C., Pigott D.M., Abbafati C., Adolph C., Amlag J.O., Aravkin A.Y. (2021). Global prevalence and burden of depressive and anxiety disorders in 204 countries and territories in 2020 due to the COVID-19 pandemic. Lancet.

[23] Oriolo G., Grande I., Martin-Santos R., Vieta E., Carvalho A.F., Baune B.T. (2018). Pathways driving neuroprogression in depression: The role of immune activation. Inflammation and Immunity in Depression: Basic Science and Clinical Applications.

[24] Ruiz N.A.L., Del Ángel D.S., Olguín H.J., Silva M.L. (2018). Neuroprogression: The hidden mechanism of depression. Neuropsychiatr. Dis. Treat..

[25] Hegeman J.M., De Waal M.W.M., Comijs H.C., Kok R.M., Van Der Mast R.C. (2015). Depression in later life: A more somatic presentation?. J. Affect. Disord..

[26] Hovatta I. (2015). Genetics: Dynamic cellular aging markers associated with major depression. Curr. Biol..

[27] Verhoeven J., Révész D., Van Oppen P., Epel E., Wolkowitz O., Penninx B. (2015). Anxiety disorders and accelerated cellular ageing. Br. J. Psychiatr..

[28] Yu R., Woo J.W. (2015). Exploring the link between depression and accelerated cellular aging: Telomeres hold the key. Res. Rep. Biochem..

[29] Agnafors S., Kjellström A.N., Torgerson J., Rusner M. (2019). Somatic comorbidity in children and adolescents with psychiatric disorders. Eur. Child Adolesc. Psychiatry.

[30] González A.C.T., Ignácio Z.M., Jornada L.K., Réus G.Z., Abelaira H.M., Santos M., Ceretta L.B., Quevedo J.L.D. (2016). Depressive disorders and comorbidities among the elderly: A population-based study. Rev. Bras. Geriatr. Gerontol..

[31] Schaakxs R., Comijs H.C., Lamers F., Beekman A.T.F., Penninx B. (2017). Age-related variability in the presentation of symptoms of major depressive disorder. Psychol. Med..

[32] Steffen A., Nübel J., Jacobi F., Bätzing J., Holstiege J. (2020). Mental and somatic comorbidity of depression: A comprehensive cross-sectional analysis of 202 diagnosis groups using German nationwide ambulatory claims data. BMC Psychiatry.

[33] Gassen N.C., Rein T. (2019). Is there a role of autophagy in depression and antidepressant action?. Front. Psychiatry.

[34] Solek P., Koszla O., Mytych J., Badura J., Chelminiak Z., Cuprys M., Fraczek J., Tabecka-Lonczynska A., Koziorowski M. (2019). Neuronal life or death linked to depression treatment: The interplay between drugs and their stress-related outcomes relate to single or combined drug therapies. Apoptosis.

[35] Collo G., Merlo Pich E. (2020). A human translational model based on neuroplasticity for pharmacological agents potentially effective in treatment-resistant depression: Focus on dopaminergic system. Neural Regen Res..

[36] Endres D., Rauer S., Venhoff N., Süß P., Dersch R., Runge K., Fiebich B.L., Nickel K., Matysik M., Maier S. (2020). Probable autoimmune depression in a patient with multiple sclerosis and antineuronal antibodies. Front. Psychiatry.

[37] Hidayat R., Saleh M.I., Parisa N. (2020). Neuronal cell death induces depressive disorder in rats depression-like behaviors caused by chronic stress. Sci. Psychiatr..

[38] Li Y., Jia Y., Wang D., Zhuang X., Li Y., Guo C., Chu H., Zhu F., Wang J., Wang X. (2021). Programmed cell death 4 as an endogenous suppressor of BDNF translation is involved in stress-induced depression. Mol. Psychiatry.

[39] Price R.B., Duman R. (2020). Neuroplasticity in cognitive and psychological mechanisms of depression: An integrative model. Mol. Psych..

[40] Kostrubiak D.E., Vacchi-Suzzi C., Smith D.M., Meliker J.R. (2017). Blood cadmium and depressive symptoms: Confounded by cigarette smoking. Psychiatry Res..

[41] Baranyi A., Meinitzer A., Rothenhäusler H.B., Amouzadeh-Ghadikolai O., Lewinski D.V., Breitenecker R.J., Herrmann M. (2018). Metabolomics approach in the investigation of depression biomarkers in pharmacologically induced immune-related depression. PLoS ONE.

[42] Clarke T.K., Zeng Y., Navrady L., Xia C., Haley C., Campbell A., Navarro P., Amador C., Adams M.J., Howard D.M. (2018). Genetic and environmental determinants of stressful life events and their overlap with depression and neuroticism. Wellcome Open Res..

[43] Gładka A., Rymaszewska J., Zatoński T. (2018). Impact of air pollution on depression and suicide. Int. J. Occup. Med. Environ. Health..

[44] Jurczak A., Brodowska A., Szkup M., Prokopowicz A., Karakiewicz B., Łój B., Kotwas A., Brodowska A., Grochans E. (2018). Influence of Pb and Cd levels in whole blood of postmenopausal women on the incidence of anxiety and depressive symptoms. Ann. Agric. Environ. Med..

[45] Losiak W., Blaut A., Kłosowska J., Losiak-Pilch J. (2019). Stressful life events, cognitive biases, and symptoms of depression in young adults. Front. Psychol..

[46] Warne N., Collishaw S., Rice F. (2019). 2019. Examining the relationship between stressful life events and overgeneral autobiographical memory in adolescents at high familial risk of depression. Memory.

[47] Zhang K., Wang X., Tu J., Rong H., Werz O., Xinchun Chen X. (2019). The interplay between depression and tuberculosis. J. Leukoc. Biol..

[48] Brietzke E., Magee T., Freire R.C.R., Gomes F.A., Milev R. (2020). Three insights on psychoneuroimmunology of mood disorders to be taken from the COVID-19 pandemic. Brain Behav. Immun. Health..

[49] Chibowska K., Chlubek D., Baranowska-Bosiacka I. (2020). Exposure to lead in the pre- and neonatal periods may result in brain inflammation. Pomeranian J. Life Sci..

[50] Yang L., Zhao Y., Wang Y., Liu L., Zhang X., Li B., Cui R. (2015). The Effects of Psychological Stress on Depression. Curr. Neuropharmacol..

[51] Dunlop B.W., Nemeroff C.B. (2007). The role of dopamine in the pathophysiology of depression. Arch. Gen. Psychiat..

[52] Belzung C., Willner P., Philippot P. (2015). Depression: From psychopathology to pathophysiology. Curr. Opin. Neurobiol..

[53] Verduijn J., Milaneschi Y., Schoevers R.A., van Hemert A.V., Beekman A.T.F., Penninx B. (2015). Pathophysiology of major depressive disorder: Mechanisms involved in etiology are not associated with clinical progression. Transl. Psychiatry.

[54] Ménard C., Hodes G.E., Russo S.J. (2016). Pathogenesis of depression: Insights from human and rodent studies. Neuroscience.

[55] Dmitrzak-Weglarz M., Reszka E. (2017). Pathophysiology of depression: Molecular regulation of melatonin homeostasis–current status. Neuropsychobiology.

[56] Jesulola E., Micalos P., Baguley I.J. (2018). Understanding the pathophysiology of depression: From monoamines to the neurogenesis hypothesis model-are we there yet?. Behav. Brain Res..

[57] Bakalov D., Hadjiolova R., Pechlivanova D. (2020). Pathophysiology of depression and novel sources of phytochemicals for its treatment–a systematic review. Acta Med. Bulg..

[58] Spellman T., Liston C. (2020). Toward circuit mechanisms of pathophysiology in depression. Am. J. Psychiatry.

[59] Chiriţă A.L., Gheorman V., Bondari D., Rogoveanu I. (2015). Current understanding of the neurobiology of major depressive disorder. Rom. J. Morphol. Embryol..

[60] Qi S., Yang X., Zhao L., Calhoun V.D., Perrone-Bizzozero N., Liu S., Jiang R., Jiang T., Sui J., Ma X. (2018). MicroRNA132 associated multimodal neuroimaging patterns in unmedicated major depressive disorder. Brain.

[61] Zhang H.F., Mellor D., Peng D.H. (2018). Neuroimaging genomic studies in major depressive disorder: A systematic review. CNS Neurosci. Ther..

[62] Howard D.M., Adams M.J., Clarke T.K., Hafferty J.D., Gibson J., Shirali M., Coleman J.R., Hagenaars S.P., Ward J., Wigmore E.M. (2019). Genome-wide meta-analysis of depression identifies 102 independent variants and highlights the importance of the prefrontal brain regions. Nat. Neurosci..

[63] Barbu M.C., Shen X., Walker R.M., Howard D.M., Evans K.L., Whalley H.C., Porteous D.J., Morris S.W., Deary I.J., Zeng Y. (2021). Epigenetic prediction of major depressive disorder. Mol. Psychiatry.

[64] Wray N.R., Ripke S., Mattheisen M., Trzaskowski M., Byrne E.M., Abdellaoui A., Bacanu S.A. (2018). Genome-wide association analyses identify 44 risk variants and refine the genetic architecture of major depression. Nat. Genet..

[65] McKeever A., Agius M., Mohr P. (2017). A review of the epidemiology of major depressive disorder and of its consequences for society and the individual. Psychiatr. Danub..

[66] Coretti S., Rumi F., Cicchetti A. (2019). The social cost of major depression: A systematic review. Rev. Eur. Stud..

[67] König H., König H., Konnopka A. (2020). The excess costs of depression: A systematic review and meta-analysis. Epidem. Psychiat. Sci..

[68] Taciak P.P., Lysenko N., Mazurek A.P. (2018). Drugs which influence serotonin transporter and serotonergic receptors: Pharmacological and clinical properties in the treatment of depression. Pharmacol. Rep..

[69] Rong H., Xu S.X., Zeng J., Yang Y.J., Zhao J., Lai W.T., Chen L.C., Deng W.F., Zhang X., Zhang Y.L. (2019). Study protocol for a parallel-group, double-blinded, randomized, controlled, noninferiority trial: The effect and safety of hybrid electroconvulsive therapy (Hybrid-ECT) compared with routine electroconvulsive therapy in patients with depression. BMC Psychiatry.

[70] Tabeshpour J., Sobhani F., Sadjadi S.A., Hosseinzadeh H., Mohajeri S.A., Rajabi O., Taherzadeh Z., Eslami S. (2017). A double-blind, randomized, placebo-controlled trial of saffron stigma (*Crocus sativus* L.) in mothers suffering from mild-to-moderate postpartum depression. Phytomedicine.

[71] Shafiee M., Arekhi S., Omranzadeh A., Sahebkar A., Shafiee M., Arekhi S., Omranzadeh A., Sahebkar A. (2018). Saffron in the treatment of depression, anxiety and other mental disorders: Current evidence and potential mechanisms of action. J. Affect. Disord..

[72] Ha A., Mehdi S., Kl K. (2019). Medicinal herbs and phytochemicals used in the treatment of depression: A review. Asian J. Pharm. Clin. Res..

[73] Choi J.E., Park D.M., Chun E., Choi J.J., Seo J.H., Kim S., Park Y.C., Jung I.C. (2017). Control of stress-induced depressive disorders by So-ochim-tang-gamibang, a Korean herbal medicine. J. Ethnopharmacol..

[74] Matraszek-Gawron R., Chwil M., Terlecka P., Skoczylas M.M. (2019). Recent studies on anti-depressant bioactive substances in selected species from the genera *Hemerocallis* and *Gladiolus*: A systematic review. Pharmaceuticals.

[75] Tóth B., Hegyi P., Lantos T., Szakacs Z., Keremi B., Varga G., Tenk J., Petervari E., Balasko M., Rumbus Z. (2019). The efficacy of saffron in the treatment of mild to moderate depression: A meta-analysis. Planta Med..

[76] Lin H.Y., Tsai J.C., Wu L.Y., Peng W.H. (2020). Reveals of new candidate active components in *Hemerocallis radix* and its anti-depression action of mechanism based on network pharmacology approach. Int. J. Mol. Sci..

[77] Lee W.H., Kim D.H., Lee T.H. (2015). Effect of mixture extracted from *Bupleuri Radix* and *Physalidis Herba* on the LPS-induced depression in rats. Korea J. Herbol..

[78] Lim D.W., Kim J.G., Han T., Jung S.K., Lim E.Y., Han D., Kim Y.T. (2015). Analgesic effect *of Ilex paraguariensis* extract on postoperative and neuropathic pain in rats. Biol. Pharm. Bull..

[79] Lutomski P., Goździewska M., Florek-Łuszczki M. (2020). Health properties of yerba mate. Ann. Agric. Environ. Med..

[80] Keefe J.R., Mao J.J., Soeller I., Li Q.S., Amsterdam J.D. (2016). Short-term open-label chamomile (*Matricaria chamomilla* L.) therapy of moderate to severe generalized anxiety disorder. Phytomedicine.

[81] Benneh C.K., Biney R.P., Adongo D.W., Mante P.K., Ampadu F.A., Tandoh A., Jato J., Woode E. (2018). Anxiolytic and antidepressant effects of *Maerua angolensis* DC. Stem bark extract in mice. Depress. Res. Treat..

[82] Shehu A., Anyip B., Magaji M.G. (2020). Antidepressant effect of methanol root bark extract of *Acacia seyal* Del. (Fabaceae): Possible involvement of the inflammatory pathway. Trop. J. Pharm. Res..

[83] Choi J.H., Lee M.J., Chang Y., Lee S., Kim H.J., Lee S.W., Kim Y.O., Cho I.H. (2020). *Valeriana fauriei* exerts antidepressant-like effects through anti-inflammatory and antioxidant activities by inhibiting brain-derived neurotrophic factor associated with chronic restraint stress. Rejuvenat. Res..

[84] Wang L., Sun Y., Zhao T., Li Y., Zhao X., Zhang L., Wu L., Zhang L., Zhang T., Wei G. (2020). Antidepressant effects and mechanisms of the total iridoids of *Valeriana jatamansi* on the brain-gut Axis. Planta Med..

[85] Chwil M., Matraszek-Gawron R., Terlecka P., Kostryco M. (2017). Plant antidepressants in selected species from the family Fabaceae—A review. Ann. Hortic..

[86] Wurglics M., Schubert-Zsilavecz M. (2006). *Hypericum perforatum*: A ‘modern’ herbal antidepressant. Clin. Pharmacokinet..

[87] Lazzara S., Napoli E., Carrubba A. (2015). *Hypericum* spp.: A resource from wild Mediterranean flora for the treatment of mild depression. Bioact. Phytochem. Perspect. Biol. Med..

[88] Zhai X.J., Chen F., Chen C., Zhu C.R., Lu Y.N. (2015). LC–MS/MS based studies on the anti-depressant effect of hypericin in the chronic unpredictable mild stress rat model. J. Ethnopharmacol..

[89] Maher A.R., Hempel S., Apaydin E., Shanman R.M., Booth M., Miles J.N., Sorbero M.E. (2016). St. John’s Wort for major depressive disorder: A systematic review. Rand. Health Q..

[90] Ng Q.X., Venkatanarayanan N., Ho C.Y. (2017). Clinical use of *Hypericum perforatum* (St. John’s wort) in depression: A meta-analysis. J. Affect. Disord..

[91] Zirak N., Shafiee M., Soltani G., Mirzaei M., Sahebkar A. (2019). *Hypericum perforatum* in the treatment of psychiatric and neurodegenerative disorders: Current evidence and potential mechanisms of action. J. Cell. Physiol..

[92] Abdelhalim A., Karim N., Chebib M., Aburjai T., Khan I., Johnston G.A., Hanrahan J. (2015). Antidepressant, anxiolytic and antinociceptive activities of constituents from *Rosmarinus officinalis*. J. Pharm. Pharm. Sci..

[93] Guo Y., Xie J., Li X., Yuan Y., Zhang L., Hu W., Luo H., Yu H., Zhang R. (2018). Antidepressant effects of rosemary extracts associate with anti-inflammatory effect and rebalance of gut microbiota. Front. Pharmacol..

[94] Ali S.S., Abd El Wahab M.G., Ayuob N.N., Suliaman M. (2017). The antidepressant-like effect of *Ocimum basilicum* in an animal model of depression. Biotech. Histochem..

[95] Guzmán-Gutiérrez S.L., Gómez-Cansino R., García-Zebadúa J.C., Jiménez-Pérez N.C., Reyes-Chilpa R. (2012). Antidepressant activity of *Litsea glaucescens* essential oil: Identification of β-pinene and linalool as active principles. J. Ethnopharmacol..

[96] El Hamdaoui Y., Zheng F., Fritz N., Ye L., Tran M.A., Schwickert K., Schirmeister T., Braeuning A., Lichtenstein D., Hellmich U.A. (2022). Analysis of hyperforin (St. John’s wort) action at TRPC6 channel leads to the development of a new class of antidepressant drugs. Mol. Psychiatry.

[97] Ayres A.S., Santos W.B., Junqueira-Ayres D.D., Costa G.M., Ramos F.A., Castellanos L., Alves J.S., Asth L., de Medeiros I.U., Zucolotto S.M. (2017). Monoaminergic neurotransmission is mediating the antidepressant-like effects of *Passiflora edulis* Sims fo. edulis. Neurosci. Lett..

[98] da Silva J.A., da Costa M., da Alves M., da Braga J., da Lima C., da Pordeus L. (2017). Effects of the single supplementation and multiple doses of *Passiflora incarnata* L. on human anxiety: A clinical trial, double-blind, placebo-controlled, randomized. Int. Arch. Med..

[99] Shahmohammadi A., Ramezanpour N., Mahdavi Siuki M., Dizavandi F., Ghazanfarpour M., Rahmani Y., Tahajjodi R., Babakhanian M. (2019). The efficacy of herbal medicines on anxiety and depression in peri- and postmenopausal women: A systematic review and meta-analysis. Post Reprod Health..

[100] Pereira M., Pereira J.A. (2015). On the effect of aromatherapy with *Citrus* fragrance in the therapy of major depressive disorder. J. Psych. Psychother..

[101] Sawamoto A., Okuyama S., Yamamoto K., Amakura Y., Yoshimura M., Nakajima M., Furukawa Y. (2016). 3, 5, 6, 7, 8, 3′, 4′-Heptamethoxyflavone, a citrus flavonoid, ameliorates corticosterone-induced depression-like behavior and restores brain-derived neurotrophic factor expression, neurogenesis, and neuroplasticity in the hippocampus. Molecules.

[102] Petreanu M., Maia P., da Rocha Pittarello J.L., Loch L.C., Monache F.D., Perez A.L., Solano-Arias G., Filho V.C., de Souza M.M., Niero R. (2019). Antidepressant-like effect and toxicological parameters of extract and withanolides isolated from aerial parts of *Solanum capsicoides* All. (Solanaceae). Naunyn-Schmiedeb. Arch. Pharmacol..

[103] Dhingra D., Valecha R. (2007). Evaluation of antidepressant-like activity of aqueous and ethanolic extracts of *Terminalia bellirica* Roxb. fruits in mice. Indian J. Exp. Biol..

[104] Mathew S.J., Shah A., Lapidus K., Clark C., Jarun N., Ostermeyer B., Murrough J.W. (2012). Ketamine for treatment-resistant unipolar depression. CNS Drugs.

[105] Simplice H.F., Emery D., Hervé N.A. (2014). Enhancing spatial memory: Anxiolytic and antidepressant effects of *Tapinanthus dodoneifolius* (DC) Danser in mice. Neurol. Res. Int..

[106] Shahamat Z., Abbasi-Maleki S., Mohammadi M.S. (2016). Evaluation of antidepressant-like effects of aqueous and ethanolic extracts of *Pimpinella anisum* fruit in mice. Avicenna J. Phytomed..

[107] Ngoupaye G.T., Bum E.N., Daniels W.M. (2013). Antidepressant-like effects of the aqueous macerate of the bulb of *Gladiolus dalenii* Van Geel (Iridaceae) in a rat model of epilepsy-associated depression. BMC Complement. Altern. Med..

[108] Ngoupaye G.T., Bum E.N., Ngah E., Talla E., Taiwe G.S., Rakotonirina A., Rakotonirina S.V. (2013). The anticonvulsant and sedative effects of *Gladiolus dalenii* extracts in mice. Epilepsy Behav..

[109] Xu P., Wang K.Z., Lu C., Dong L.M., Le Z.J., Liao Y.H., Liu X.M. (2016). Antidepressant-like effects and cognitive enhancement of the total phenols extract of *Hemerocallis citrina* Baroni in chronic unpredictable mild stress rats and its related mechanism. J. Ethnopharmacol..

[110] Marchyshyn S.M., Zarichanska O.V., Cholach S.Y. (2016). Investigation of acute toxicity and neurotropic properties of the flowers’dense extracts of tawny daylily (*Hemerocallis fulva* L.) and hybrid daylily (*Hemerocallis hybrida* var. Stella De Oro). Farm. čas..

[111] Yang L., Shergis J.L., Di Y.M., Zhang A.L., Lu C., Guo X., Fang Z., Xue C.C., Li Y. (2020). Managing depression with *Bupleurum chinense* herbal formula: A systematic review and meta-analysis of randomized controlled trials. J. Alter. Compl. Med..

[112] Bagci E., Aydin E., Mihasan M., Maniu C., Hritcu L. (2016). Anxiolytic and antidepressant-like effects of *Ferulago angulata* essential oil in the scopolamine rat model of Alzheimer’s disease. Flavour Fragr. J..

[113] Dereli F.T.G., Ilhan M., Akkol E.K. (2020). Identification of the main active antidepressant constituents in a traditional Turkish medicinal plant, *Centaurea kurdica* Reichardt. J. Ethnopharmacol..

[114] Ioniță R., Postu P.A., Cioancă O., Mircea C., Hăncianu M., Hrițcu L., Mircea C. (2019). Anxiolytic and antidepressant effects of *Matricaria chamomilla* hydroalcoholic extract in a rat model of scopolamine. Farmacia.

[115] Cárdenas J., Reyes-Pérez V., Hernández-Navarro M.D., Dorantes-Barrón A.M., Almazán S., Estrada-Reyes R. (2017). Anxiolytic- and antidepressant-like effects of an aqueous extract of *Tanacetum parthenium* L. Schultz-Bip (Asteraceae) in mice. J. Ethnopharmacol..

[116] Cornara L., Ambu G., Trombetta D., Denaro M., Alloisio S., Frigerio J., Labra M., Ghimire G., Valussi M., Smeriglio A. (2020). Comparative and functional screening of three species traditionally used as antidepressants: *Valeriana officinalis* L., *Valeriana jatamansi* Jones ex Roxb. and *Nardostachys jatamansi* (D. Don) DC. Plants.

[117] Gill M., Kinra M., Rai A., Chamallamudi M.R., Kumar N. (2018). Evaluation of antidepressant activity of methanolic extract of *Saraca asoca* bark in a chronic unpredictable mild stress model. Neuroreport.

[118] Sanchez-Mateo C.C., Prado B., Rabanal R.M. (2002). Antidepressant effects of the methanol extract of several *Hypericum* species from the Canary Islands. J. Ethnopharmacol..

[119] Rahmati B., Kiasalari Z., Roghani M., Khalili M., Ansari F. (2017). Antidepressant and anxiolytic activity of *Lavandula officinalis* aerial parts hydroalcoholic extract in scopolamine-treated rats. Pharm. Biol..

[120] Nematolahi P., Mehrabani M., Karami-Mohajeri S., Dabaghzadeh F. (2018). Effects of *Rosmarinus officinalis* L. on memory performance, anxiety, depression, and sleep quality in university students: A randomized clinical trial. Complem. Ther. Clin. Pract..

[121] Choukairi Z., Hazzaz T., Lkhider M., Ferrandez J.M., Fechtali T. (2019). Effect of *Salvia officinalis* L. and *Rosmarinus officinalis* L. leaves extracts on anxiety and neural activity. Bioinformation.

[122] Adel A., Ikram R., Wasi N. (2019). *Salvia hispanica* (White chia): A new window for its antidepressant and memory boosting activity. Pak. J. Pharm. Sci..

[123] Sarkoohi P., Fathalipour M., Ghasemi F., Javidnia K., Emamghoreishi M. (2020). Antidepressant effects of the aqueous and hydroalcoholic extracts of *Salvia mirzayanii* and *Salvia macrosiphon* in male mice. Shiraz. E-Med. J..

[124] El Gabbas Z., Bezza K., Laadraoui J., Makbal R., Aboufatima R., Chait A. (2018). *Salvia officinalis* induces antidepressant-like effect, anxiolytic activity and learning improvement in hippocampal lesioned and intact adult rats. Bangladesh J. Pharmacol..

[125] Hamann F.R., Zago A.M., Rossato M.F., Beck V.R., Mello C.F., de Brum T.F., de Carvalho L.M., Faccin H., Oliveira S.M., Rubin M.A. (2016). Antinociceptive and antidepressant-like effects of the crude extract of *Vitex megapotamica* in rats. J. Ethnopharmacol..

[126] Devika M., Nalini M.S. (2018). Evaluation of antidepressant activity of *Litsea floribunda* (Bl.) Gamble-Lauraceae using animal models. Int. J. Pharm. Sci. Res..

[127] Fiebich B.L., Knörle R., Appel K., Kammler T., Weiss G. (2011). Pharmacological studies in an herbal drug combination of St. John’s Wort (*Hypericum perforatum*) and passion flower (*Passiflora incarnata*): In *vitro* and *in vivo* evidence of synergy between *Hypericum* and *Passiflora* in antidepressant pharmacological models. Fitoterapia.

[128] Alves J., Silva A., da Silva R.M., Tiago P., de Carvalho T.G., de Araújo R.F., de Azevedo E.P., Lopes N.P., Ferreira L.S., Gavioli E.C. (2020). *In Vivo* antidepressant effect of *Passiflora edulis* f. *flavicarpa* into cationic nanoparticles: Improving bioactivity and safety. Pharmaceutics.

[129] Jafarpoor N., Abbasi-Maleki S., Asadi-Samani M., Khayatnouri M.H. (2014). Evaluation of antidepressant-like effect of hydroalcoholic extract of *Passiflora incarnata* in animal models of depression in male mice. J. Herb.Med. Pharmacol..

[130] Bettio L.E., Machado D.G., Cunha M.P., Capra J.C., Missau F.C., Santos A.R., Pizzolatti M.G., Rodrigues A.L. (2011). Antidepressant-like effect of extract from *Polygala paniculata*: Involvement of the monoaminergic systems. Pharm. Biol..

[131] Ibrahim M., Amin M.N., Millat M.S., Raju J.A., Hussain M.S., Sultana F., Islam M.M., Hasan M.M. (2018). Methanolic extract of peel of *Citrus maxima* fruits exhibit analgesic, CNS depressant and anti-inflammatory activities in swiss albino mice. BEMS Rep..

[132] Barua C.C., Haloi P., Saikia B., Sulakhiya K., Pathak D.C., Tamuli S., Rizavi H., Ren X. (2018). *Zanthoxylum alatum* abrogates lipopolysaccharide-induced depression-like behaviours in mice by modulating neuroinflammation and monoamine neurotransmitters in the hippocampus. Pharm. Biol..

[133] Kumar R., Singh V., Devi K., Sharma M., Singh M.K., Ahuja P.S. (2008). State of art of saffron (*Crocus sativus* L.) agronomy: A comprehensive review. Food Rev. Int..

[134] Hosseinzadeh H., Nassiri-Asl M. (2013). Avicenna’s (Ibn Sina) the canon of medicine and saffron (*Crocus sativus*): A review. Phytother. Res..

[135] Javadi B., Sahebkar A., Emami S.A. (2013). A survey on saffron in major islamic traditional medicine books. Iran. J. Basic. Med. Sci..

[136] Jan S., Wani A.A., Kamili A.N., Kashtwari M. (2014). Distribution, chemical composition and medicinal importance of saffron (*Crocus sativus* L.). Afr. J. Plant..

[137] Ngoupaye G.T., Bum E.N., Taiwe G.S., Moto F.C., Talla E. (2013). Antidepressant properties of aqueous macerate from *Gladiolus dalenii* corms. Afr. J. Tradit. Complement. Altern. Med..

[138] Nemati Z., Harpke D., Gemicioglu A., Kerndorff H., Blattner F.R. (2019). Saffron (*Crocus sativus*) is an autotriploid that evolved in Attica (Greece) from wild *Crocus cartwrightianus*. Mol. Phylogenet. Evol..

[139] Shokrpour M., Al-Khayri J.M., Jain S.M., Johnson D.V. (2019). Saffron (*Crocus sativus* L.) breeding: Opportunities and challenges. Advances in Plant Breeding Strategies: Industrial and Food Crops.

[140] El Midaoui A., Ghzaiel I., Vervandier-Fasseur D., Ksila M., Zarrouk A., Nury T., Khallouki F., El Hessni A., Ibrahimi S.O., Latruffe N. (2022). Saffron (*Crocus sativus* L.): A source of nutrients for health and for the treatment of neuropsychiatric and age-related diseases. Nutrients.

[141] Kumar A., Devi M., Kumar R., Kumar S. (2022). Introduction of high-value *Crocus sativus* (saffron) cultivation in non-traditional regions of India through ecological modelling. Sci. Rep..

[142] Fujii S., Morita Y., Ohta T., Uto T., Shoyama Y. (2022). Saffron (*Crocus sativus* L.) as a valuable spice and food product: A narrative review. Longhua Chin. Med..

[143] Gresta F., Lombardo G.M., Siracusa L., Ruberto G. (2008). Saffron, an alternative crop for sustainable agricultural systems. a review. agronomy for sustainable development. Agron. Sustain. Dev..

[144] Grilli Caiola M., Canini A. (2010). Looking for saffron’s (*Crocus sativus* L.) parents. Funct. Plant Sci. Biotechnol..

[145] Ahmad M., Zaffar G., Habib M., Arshid A., Dar N.A., Dar Z.A. (2014). Saffron (*Crocus sativus* L.) in the light of biotechnological approaches: A review. Sci. Res. Essays.

[146] Dar R.A., Shahnawaz M., Malik S.B., Sangale M.K., Ade A.B., Qazi P.H. (2017). Cultivation, distribution, taxonomy, chemical composition and medical importance of *Crocus sativus*. J. Phytopharmacol..

[147] Torricelli R., Yousefi J., Albertini E., Venanzoni R., Hosseinzadeh Y. (2019). Morphological and molecular characterization of Italian, Iranian and Spanish saffron (*Crocus sativus* L.) accessions. Appl. Ecol. Environ. Res..

[148] Ulbricht C., Conquer J., Costa D., Hollands W., Iannuzzi C., Isaac R., Jordan J.K., Ledesma N., Ostroff C., Serrano J.M. (2011). An evidence-based systematic review of saffron (*Crocus sativus*) by the Natural Standard Research Collaboration. J. Diet. Suppl..

[149] Qian X., Sun Y., Zhou G., Yuan Y., Li J., Huang H., Xu L., Li L. (2019). Single-molecule real-time transcript sequencing identified flowering regulatory genes in *Crocus sativus*. BMC Genom..

[150] Pandita D., Aftab T., Hakeem K.R. (2021). Saffron (*Crocus sativus* L.): Phytochemistry, therapeutic significance and omics-based biology. Medicinal and Aromatic Plants.

[151] He F., Dai Z., He Y., Zhang Y.M., Lu J. (2015). Study on crocins reference extract and application on assay of Croci Stigma. Chin. J. Chin. Mat. Med..

[152] Al-Snafi A.E. (2016). The pharmacology of *Crocus sativus*-a review. IOSR J. Pharm..

[153] Zhou G.F., Liu Y.Y., Qian X.D., Yao C., Chen S.H. (2016). Improvement of quality evaluation of Croci Stigma in the process of producing and distributing based on Chinese pharmacopoeia and ISO*. Chin. J. Pharm. Anal..

[154] Melnyk J.P., Wang S., Marcone M.F. (2010). Chemical and biological properties of the world’s most expensive spice: Saffron. Food. Res. Int..

[155] Kyriakoudi A., Ordoudi A., Roldán-Medina M., Tsimidou Z. (2015). Saffron, a functional spice. Austin J. Nutri. Food Sci..

[156] Kafi M., Kamili A.N., Husaini A.M., Ozturk M., Altay V., Ozturk M., Hakeem K.R., Ashraf M., Ahmad M.S.A. (2018). An expensive spice saffron (*Crocus sativus* L.): A case study from Kashmir, Iran, and Turkey. Global Perspectives on Underutilized Crops.

[157] Hosseini A., Razavi B.M., Hosseinzadeh H. (2018). Saffron (*Crocus sativus*) petal as a new pharmacological target: A review. Iran. J. Basic Med. Sci..

[158] Cardone L., Castronuovo D., Perniola M., Cicco N., Candido V. (2020). Saffron (*Crocus sativus* L.) the king of spices: An overview. Sci. Hort..

[159] Kothari D., Thakur R., Kumar R. (2021). Saffron (*Crocus sativus* L.): Gold of the spices—A comprehensive review. Hortic. Environ. Biotechnol..

[160] Xing B., Li S., Yang J., Lin D., Feng Y., Lu J., Shao Q. (2021). Phytochemistry, pharmacology, and potential clinical applications of saffron: A review. J. Ethnopharmacol..

[161] Chen J., Zhou G., Dong Y., Qian X., Li J., Xu X., Huang H., Xu L., Li L. (2021). Screening of key proteins affecting floral initiation of saffron under cold stress using iTRAQ-based proteomics. Front. Plant Sci..

[162] Gohari A.R., Saeidnia S., Mahmoodabadi M.K. (2013). An overview on saffron, phytochemicals, and medicinal properties. Pharmacog. Rev..

[163] Malathi M., Devi D.R., Hari B.V. (2014). *Crocus sativus* Linn–a potential source for diverse therapeutic applications. Int. J. Pharm. Sci. Rev. Res..

[164] Rahimi M. (2015). Chemical and medicinal properties of saffron. Bull. Environ. Pharmacol. Life Sci..

[165] Kosar M., Demirci B., Goger F., Kara I., Baser K.H.C. (2017). Volatile composition, antioxidant activity, and antioxidant components in saffron cultivated in Turkey. Int. J. Food Prop..

[166] Moratalla-López N., Bagur M.J., Lorenzo C., Martínez-Navarro M.E., Salinas M.R., Alonso G.L. (2019). Bioactivity and bioavailability of the major metabolites of *Crocus sativus* L. flower. Molecules.

[167] Mykhailenko O., Kovalyov V., Goryacha O., Ivanauskas L., Georgiyants V. (2019). Biologically active compounds and pharmacological activities of species of the genus *Crocus*: A review. Phytochemistry.

[168] Jafari S.M., Mahdavee Khazaei K., Assadpour E. (2019). Production of a natural color through microwave-assisted extraction of saffron tepal’s anthocyanins. Food Sci. Nutr..

[169] Goupy P., Vian M.A., Chemat F., Caris-Veyrat C. (2013). Identification and quantification of flavonols, anthocyanins and lutein diesters in tepals of *Crocus sativus* by ultra performance liquid chromatography coupled to diode array and ion trap mass spectrometry detections. Ind. Crop. Prod..

[170] Baba S.A., Malik A.H., Wani Z.A., Mohiuddin T., Shah Z., Abbas N., Ashraf N. (2015). Phytochemical analysis and antioxidant activity of different tissue types of *Crocus sativus* and oxidative stress alleviating potential of saffron extract in plants, bacteria, and yeast. S. Afr. J. Bot..

[171] Karimi E., Oskoueian E., Hendra R., Jaafar H.Z. (2010). Evaluation of *Crocus sativus* L. stigma phenolic and flavonoid compounds and its antioxidant activity. Molecules.

[172] Mir M.A., Parihar K., Tabasum U., Kumari E. (2016). Estimation of alkaloid, saponin and flavonoid, content in various extracts of *Crocus sativa*. J. Med. Plants Stud..

[173] Goli S.A.H., Mokhtari F., Rahimmalek M. (2012). Phenolic compounds and antioxidant activity from saffron (*Crocus sativus* L) petal. J. Agric. Sci..

[174] Masuda A., Mori K., Oda Y., Miyazawa M. (2010). Volatile oil compounds from corms and flowers of *Crocus vernus* L. Hill and corms of *C. sativus* L.. Libyan Agric. Res. Cen. J. Int..

[175] Karimi G.R., Hosseinzadeh H., Khaleghpanah P. (2001). Study of antidepressant effect of aqueous and ethanolic extract of *Crocus sativus* in mice. Iran J. Basic Med. Sci..

[176] Hosseinzadeh H., Younesi H.M. (2002). Antinociceptive and anti-inflammatory effects of *Crocus sativus* L. stigma and petal extracts in mice. BMC Pharmacol..

[177] Akhondzadeh S., Fallah-Pour H., Afkham K., Jamshidi A.H., Khalighi-Cigaroudi F. (2004). Comparison of *Crocus sativus* L. and imipramine in the treatment of mild to moderate depression: A pilot double-blind randomized trial [ISRCTN45683816]. BMC Complement. Altern. Med..

[178] Akhondzadeh S., Tahmacebi-Pour N., Noorbala A.A., Amini H., Fallah Pour H., Jamshidi A.H., Khani M. (2005). *Crocus sativus* L. in the treatment of mild to moderate depression: A double-blind, randomized and placebo controlled trial. Phytother. Res..

[179] Kamalipour M., Jamshidi A.H., Akhondzadeh S. (2010). Antidepressant effect of *Crocus sativus*: An evidence based review. J. Med. Plants..

[180] Mousavi S.Z., Bathaie S.Z. (2011). Historical uses of saffron: Identifying potential new avenues for modern research. Avicenna J. Phytomed..

[181] Moshiri M., Vahabzadeh M., Hosseinzadeh H. (2015). Clinical applications of saffron (*Crocus sativus*) and its constituents: A review. Drug Res. Stuttg..

[182] Selamoglu Z., Ozgen S. (2016). Therapeutic potential of saffron crocus (*Crocus sativus* L.). Turkish JAF Sci. Tech..

[183] Feldman E. (2017). Saffron and depression: What do we know and where do we go?. Integr. Med. Alert..

[184] Loizzo M.R., Marrelli M., Pugliese A., Conforti F., Nadjafi F., Menichini F., Tundis R. (2016). *Crocus cancellatus* subsp. *Damascenus* stigmas: Chemical profile, and inhibition of α-amylase, α-glucosidase and lipase, key enzymes related to type 2 diabetes and obesity. J. Enzyme Inhib. Med. Chem..

[185] Ashktorab H., Soleimani A., Singh G., Amin A., Tabtabaei S., Latella G., Stein U., Akhondzadeh S., Solanki N., Gondré-Lewis M.C. (2019). Saffron: The golden spice with therapeutic properties on digestive diseases. Nutrients.

[186] Ghaderi A., Asbaghi O., Reiner Ž., Kolahdooz F., Amirani E., Mirzaei H., Banafshe H.R., Dana P.M., Asemi Z. (2020). The effects of saffron (*Crocus sativus* L.) on mental health parameters and C-reactive protein: A meta-analysis of randomized clinical trials. Complement. Ther. Med..

[187] Abdullaev F. (2007). Biological properties and medicinal use of saffron (*Crocus sativus* L.). Acta Hortic..

[188] Bhargava B.K. (2011). Medicinal uses and pharmacological properties of *Crocus sativus* Linn. (saffron). Int. J. Pharm. Pharm. Sci..

[189] Poma A., Fontecchio G., Carlucci G., Chichiricco G. (2012). Anti-inflammatory properties of drugs from saffron crocus. Antiinflamm. Antiallergy Agents Med. Chem..

[190] Bahramsoltani R., Farzaei M.H., Rahimi R. (2014). Medicinal plants and their natural components as future drugs for the treatment of burn wounds: An integrative review. Arch. Dermatol. Res..

[191] Christodoulou E., Kadoglou N.P., Kostomitsopoulos N., Valsami G. (2015). Saffron: A natural product with potential pharmaceutical applications. J. Pharm. Pharmacol..

[192] Khorasany A.R., Hosseinzadeh H. (2016). Therapeutic effects of saffron (*Crocus sativus* L.) in digestive disorders: A review. Iran J. Basic. Med. Sci..

[193] Vyas L.K., Tapar K.K., Nema R.K. (2010). Study of *Crocus sativus* as complexion promoter in skin care. Int. J. Pharm. Clin. Res..

[194] Bathaie S.Z., Farajzade A., Hoshyar R. (2014). A review of the chemistry and uses of crocins and crocetin, the carotenoid natural dyes in saffron, with particular emphasis on applications as colorants including their use as biological stains. Biotech. Histochem..

[195] Mzabri I., Addi M., Berrichi A. (2019). Traditional and modern uses of saffron (*Crocus sativus*). Cosmetics.

[196] Licón C.C., Carmona M., Rubio R., Molina A., Berruga M.I. (2012). Preliminary study of saffron (*Crocus sativus* L. stigmas) color extraction in a dairy matrix. Dyes Pigm..

[197] Çelik S.Y. (2018). Purification of protease enzyme from çiğdem (*Crocus biflorus*) tubers and investigation of usability of the purified protease enzyme in coagulation of casein. GIDA.

[198] Shariati-Moghaddam A. (2004). New saffron products and food technology. Acta Hortic..

[199] Serrano-Díaz J., Sánchez A.M., Martínez-Tomé M., Winterhalter P., Alonso G.L. (2013). A contribution to nutritional studies on *Crocus sativus* flowers and their value as food. J. Food Compos. Anal..

[200] Almodóvar P., Prodanov M., Arruñada O., Inarejos-García A.M., Almodóvar P., Prodanov M., Arruñada O., Inarejos-García A.M. (2018). Saffron eye, a natural extract of saffron (*Crocus sativus* L.) with colorant properties as novel replacer of saffron stigmas in culinary and food applications. Int. J. Gastron. Food Sci..

[201] Bagur M., Alonso Salinas G.L., Jiménez-Monreal A.M., Chaouqi S., Llorens S., Martínez-Tomé M., Alonso G.L. (2018). Saffron: An old medicinal plant and a potential novel functional food. Molecules.

[202] Takur M., Sharma N. (2014). Saffron: A golden condiment and a respository of nutraceutical potential. Food Sci..

[203] Sarfarazi M., Jafari S.M., Rajabzadeh G. (2015). Extraction optimization of saffron nutraceuticals through response surface methodology. Food Anal. Methods.

[204] Bathaie S.Z. (2018). Saffron as a functional food and a nutraceutical using saffron and its constituents as the nutraceutics to protect against chronic diseases. Acta Hortic..

[205] Khan A., Muhamad N.A., Ismail H., Nasir A., Khalil A.A.K., Anwar Y., Khan Z., Ali A., Taha R.M., Al-Shara B. (2020). Potential nutraceutical benefits of *in vivo* grown saffron (*Crocus sativus* L.) as analgesic, anti-inflammatory, anticoagulant, and antidepressant in mice. Plants.

[206] Merrell J.G., McLaughlin S.W., Tie L., Laurencin C.T., Chen A.F., Nair L.S. (2009). Curcumin-loaded poly (ε-caprolactone) nanofibres: Diabetic wound dressing with anti-oxidant and anti-inflammatory properties. Clin. Exp. Pharmacol. Physiol..

[207] Thamer N.A., AL-Mashhady L.A. (2016). Acute toxicity of green synthesis of silver nanoparticles using *Crocus sativus* L. on white albino rats. Int. J. Phytopharm..

[208] Yildiztekin M., Nadeem S., Yildiztekin F., Varol O., Ozler M.A., Tuna A.L. (2017). Green synthesis and characterization of silver nanoparticles from *Crocus mathewii*; a disremembered Turkish flowering plant. Indian J. Pharm. Sci..

[209] Bagherzade G., Tavakoli M.M., Namaei M.H. (2017). Green synthesis of silver nanoparticles using aqueous extract of saffron (*Crocus sativus* L.) wastages and its antibacterial activity against six bacteria. Asian Pac. J. Trop. Biomed..

[210] Mirhadi E., Nassirli H., Malaekeh-Nikouei B. (2020). An updated review on therapeutic effects of nanoparticle-based formulations of saffron components (safranal, crocin, and crocetin). J. Pharm. Investig..

[211] Hafezi Ghahestani Z., Alebooye Langroodi F., Mokhtarzadeh A., Ramezani M., Hashemi M. (2017). Evaluation of anti-cancer activity of PLGA nanoparticles containing crocetin. Artif. Cells Nanomed. Biotechnol..

[212] Azizian-Shermeh O., Valizadeh M., Taherizadeh M., Beigomi M. (2020). Phytochemical investigation and phytosynthesis of eco-friendly stable bioactive gold and silver nanoparticles using petal extract of saffron (*Crocus sativus* L.) and study of their antimicrobial activities. Appl. Nanosci..

[213] Zeka K., Ruparelia K.C., Sansone C., Macchiarelli G., Continenza M.A., Arroo R.R. (2018). New hydrogels enriched with antioxidants from saffron crocus can find applications in wound treatment and/or beautification. Skin Pharmacol. Physiol..

[214] Schmidt M., Betti G., Hensel A. (2007). Saffron in phytotherapy: Pharmacology and clinical uses. Wien Med. Wochenschr..

[215] Gutheil G.W., Reed G., Ray A., Anant S., Dhar A. (2012). Crocetin: An agent derived from saffron for prevention and therapy for cancer derived from saffron for prevention and therapy for cancer. Curr. Pharm. Biotechnol..

[216] Talaei A., Hassanpour Moghadam M., Sajadi Tabassi S.A., Mohajeri S.A. (2015). Crocin, the main active saffron constituent, as an adjunctive treatment in major depressive disorder: A randomized, double-blind, placebo-controlled, pilot clinical trial. J. Affect. Disord..

[217] Jafarnia N., Ghorbani Z., Nokhostin M., Manayi A., Nourimajd S., Jahromi S.R. (2017). Effect of saffron (*Crocus sativus* L.) as an add-on therapy to sertraline in mild to moderate generalized anxiety disorder: A double blind randomized controlled trial. Arch. Neurosci..

[218] Zhang Z., Wang C.Z., Wen X.D., Shoyama Y., Yuan C.S. (2013). Role of saffron and its constituents on cancer chemoprevention. Pharm. Biol..

[219] Bhandari P.R. (2015). *Crocus sativus* L. (saffron) for cancer chemoprevention: A mini review. J. Tradit. Complement. Med..

[220] Naeimi M., Shafiee M., Kermanshahi F., Khorasanchi Z., Khazaei M., Ryzhikov M., Avan A., Gorji N., Hassanian S.M. (2019). Saffron (*Crocus sativus*) in the treatment of gastrointestinal cancers: Current findings and potential mechanisms of action. J. Cell. Biochem..

[221] Dawalbhakta M., Telang M. (2017). Patents on therapeutic and cosmetic applications of bioactives of *Crocus sativus* L. and their production through synthetic biology methods: A review. Ther. Pat. Eotpeg..

[222] Alsayied N.F., Fernández J.A., Schwarzacher T., Heslop-Harrison J.S. (2015). Diversity and relationships of *Crocus sativus* and its relatives analysed by inter-retroelement amplified polymorphism (IRAP). Ann. Bot..

[223] Gedik A., Duygu A., Erdogmus S., Comertpay G., Tanyolac M.B., Ozkan H. (2017). Genetic diversity of *Crocus sativus* and its close relative species analyzed by iPBS-retrotransposons. Turk. J. Field Crop..

[224] Bayat M., Amirnia R., Özkan H., Gedik A., Ates D., Tanyulac B., Rahimi M. (2018). Diversity and phylogeny of saffron (*Crocus sativus* L.) accessions based on IPBS markers. Genetica.

[225] Yousefi Javan I., Gharari F. (2018). Genetic diversity in saffron (*Crocus sativus* L.) cultivars grown in Iran using SSR and SNP markers. J. Agric. Sci. Technol..

[226] Wang Y., Han T., Zhu Y., Zheng C.J., Ming Q.L., Rahman K., Qin L.P. (2010). Antidepressant properties of bioactive fractions from the extract of *Crocus sativus* L.. J. Nat. Med..

[227] Lopresti A.L., Maker G.L., Hood S.D., Drummond P.D. (2014). A review of peripheral biomarkers in major depression: The potential of inflammatory and oxidative stress biomarkers. Prog. Neuro-Psychopharmacol. Biol. Psychiatry.

[228] Lopresti A.L., Drummond P.D. (2014). Saffron (*Crocus sativus*) for depression: A systematic review of clinical studies and examination of underlying antidepressant mechanisms of action. Hum. Psychopharmacol. Clin. Exp..

[229] Ghasemi T., Abnous K., Vahdati F., Mehri S., Razavi B., Hosseinzadeh H. (2015). Antidepressant effect of *Crocus sativus* aqueous extract and its effect on CREB, BDNF, and VGF transcript and protein levels in rat hippocampus. Drug Res..

[230] Pitsikas N., Boultadakis A., Georgiadou G., Tarantilis P.A., Sakellaridis N. (2008). Effects of the active constituents of *Crocus sativus* L., crocins, in an animal model of anxiety. Phytomedicine.

[231] Hosseinzadeh H., Noraei N.B. (2009). Anxiolytic and hypnotic effect of *Crocus sativus* aqueous extract and its constituents, crocin and safranal, in mice. Phytother Res..

[232] Mazidi M., Shemshian M., Mousavi S.H., Norouzy A., Kermani T., Moghiman T., Sadeghi A., Mokhber N., Ghayour-Mobarhan M., Ferns G.A. (2016). A double-blind, randomized and placebo-controlled trial of saffron (*Crocus sativus* L.) in the treatment of anxiety and depression. J. Complement. Integr. Med..

[233] Pitsikas N. (2016). Constituents of saffron (*Crocus sativus* L.) as potential candidates for the treatment of anxiety disorders and schizophrenia. Molecules.

[234] Lopresti A.L., Drummond P.D., Inarejos-García A.M., Prodanov M. (2018). Affron, a standardised extract from saffron (*Crocus sativus* L.) for the treatment of youth anxiety and depressive symptoms: A randomised, double-blind, placebo-controlled study. J. Affect. Disord..

[235] Nam K.N., Park Y.M., Jung H.J., Lee J.Y., Min B.D., Park S.U., Jung W.S., Cho K.H., Park J.H., Kang I. (2010). Anti-inflammatory effects of crocin and crocetin in rat brain microglial cells. Eur. J. Pharmacol..

[236] Kumar V., Bhat Z.A., Kumar D., Khan N.A., Chashoo I.A., Shah M.Y. (2012). Evaluation of anti-inflammatory potential of petal extracts of *Crocus sativus* ‘Cashmerianus’. Intl. J. Pjournal. Phytopharmacol..

[237] Rathore B., Jaggi K., Thakur S.K., Mathur A., Mahdi F. (2015). Anti-inflammatory activity of *Crocus sativus* extract in experimental arthritis. Int. J. Pharm. Sci. Res..

[238] Zhang C., Ma J., Fan L., Zou Y., Dang X., Wang K., Song J. (2015). Neuroprotective effects of safranal in a rat model of traumatic injury to the spinal cord by anti-apoptotic, anti-inflammatory and edema-attenuating. Tissue Cell..

[239] Boskabady M.H., Farkhondeh T. (2016). Anti-inflammatory, antioxidant, and immunomodulatory effects of *Crocus sativus* L. and its main constituents. Phytother. Res..

[240] Kermani T., Zebarjadi M., Mehrad-Majd H., Mirhafez S.R., Shemshian M., Ghasemi F., Mohammadzadeh E., Mousavi S.H., Norouzy A., Moghiman T. (2017). Anti-inflammatory effect of *Crocus sativus* on serum cytokine levels in subjects with metabolic syndrome: A randomized, double-blind, placebo-controlled trial. Curr. Clin. Pharmacol..

[241] Yarijani Z.M., Pourmotabbed A., Pourmotabbed T., Najafi H. (2017). Crocin has anti-inflammatory and protective effects in ischemia-reperfusion induced renal injuries. Iran J. Basic Med. Sci..

[242] Zeinali M., Zirak M.R., Rezaee S.A., Karimi G., Hosseinzadeh H. (2019). Immunoregulatory and anti-inflammatory properties of *Crocus sativus* (Saffron) and its main active constituents: A review. Iran J. Basic Med. Sci..

[243] Liu M., Amini A., Ahmad Z. (2017). Safranal and its analogs inhibit *Escherichia coli* ATP synthase and cell growth. Int. J. Biol. Macromol..

[244] Abduallev F.I. (2002). Cancer chemopreventive and tumoricidal properties of saffron (*Crocus sativus* L.). Exp. Biol. Med..

[245] Abdullaev F.I., Riveron-Negrete L., Caballero-Ortega H., Hernández J.M., Perez-Lopez I., Pereda-Miranda R., Espinosa-Aguirre J.J. (2003). Use of *in vitro* assays to assess the potential antigenotoxic and cytotoxic effects of saffron (*Crocus sativus* L.). Toxicol. In Vitr..

[246] Abdullaev F.I. (2004). Antitumor effect of saffron (*Crocus sativus* L.): Overview and perspectives. Acta Hortic..

[247] Fernández J.A. (2006). Anticancer properties of saffron, *Crocus sativus* Linn. Adv. Phytomed..

[248] Premkumar K., Thirunavukkarasu C., Abraham S.K., Santhiya S.T., Ramesh A. (2006). Protective effect of saffron (*Crocus sativus* L.) aqueous extract against genetic damage induced by anti-tumor agents in mice. Hum. Exp. Toxicol..

[249] Chryssanthi D.G., Lamari F.N., Iatrou G., Pylara A., Karamanos N.K., Cordopatis P. (2007). Inhibition of breast cancer cell proliferation by style constituents of different *Crocus* species. Anticancer Res..

[250] Abdullaev F.I., Riveron-Negrete L., Caballero-Ortega H., Hernández J.M., Perez-Lopez I., Pereda-Miranda R., Espinosa-Aguirre J.J. (2010). Use of *in vitro* assays to assess the potential antiproliferative and cytotoxic effects of saffron (*Crocus sativus* L.) in human lung cancer cell line. Pharmacogn. Mag..

[251] Samarghandian S., Borji A. (2014). Anticarcinogenic effect of saffron (*Crocus sativus* L.) and its ingredients. Pharmacogn. Res..

[252] Gezici S. (2019). Comparative anticancer activity analysis of saffron extracts and a principle component, crocetin for prevention and treatment of human malignancies. J. Food Sci. Technol..

[253] Hosseinzadeh H., Shariaty V.M. (2007). Anti-nociceptive effect of safranal, a constituent of *Crocus sativus* (saffron), in mice. Pharmacologyonline.

[254] Amin B., Hosseini S., Hosseinzadeh H. (2017). Enhancement of antinociceptive effect by co-administration of amitriptyline and *Crocus sativus* in a rat model of neuropathic pain. Iran. J. Pharm. Res..

[255] Li Puma S., Landini L., Macedo S.J., Seravalli V., Marone I.M., Coppi E., Patacchini R., Geppetti P., Materazzi S., Nassini R. (2019). TRPA 1 mediates the antinociceptive properties of the constituent of *Crocus sativus* L., safranal. J. Cell Mol. Med..

[256] Hosseinzadeh H., Khosravan V. (2002). Anticonvulsant effect of *Crocus sativus* L. stigmas aqueous and ethanolic extracts in mice. Arch. Iran Med..

[257] Hosseinzadeh H., Talebzadeh F. (2005). Anticonvulsant evaluation of safranal and crocin from *Crocus sativus* in mice. Fitoterapia.

[258] Hosseinzadeh H., Ghenaati J. (2006). Evaluation of the antitussive effect of stigma and petals of saffron (*Crocus sativus*) and its components, safranal and crocin in guinea pigs. Fitoterapia.

[259] Tavakkol-Afshari J., Brook A., Mousavi S.H. (2008). Study of cytotoxic and apoptogenic properties of saffron extract in human cancer cell lines. Food Chem. Toxicol..

[260] Vazifedan V., Mousavi S.H., Sargolzaei J., Soleymanifard S., Pakdel A.F. (2017). Study of crocin radiotherapy-induced cytotoxicity and apoptosis in the head and neck cancer (HN-5) cell line. Iran J. Pharm. Res..

[261] Koul A., Abraham S.K. (2018). Efficacy of crocin and safranal as protective agents against genotoxic stress induced by gamma radiation, urethane and procarbazine in mice. Hum. Exp. Toxicol..

[262] Boskabady M.H., Aslani M.R. (2006). Relaxant effect of *Crocus sativus* (saffron) on guinea-pig tracheal chains and its possible mechanisms. J. Pharm. Pharmacol..

[263] Mokhtari-Zaer A., Khazdair M.R., Boskabady M.H. (2015). Smooth muscle relaxant activity of *Crocus sativus* (saffron) and its constituents: Possible mechanisms. Avicenna J. Phytomed..

[264] Fatehi M., Rashidabady T., Fatehi-Hassanabad Z. (2003). Effects of *Crocus sativus* petals’ extract on rat blood pressure and on response induced by electrical field stimulation in the rat isolated vas deferens and guinea-pig ileum. J. Ethnopharmacol..

[265] Imenshahidi M., Razavi B.M., Faal A., Gholampoor A., Mehran S. (2013). The effect of chronic administration of saffron (*Crocus sativus*) stigma aqueous extract on systolic blood pressure in rats. Jundishapur J. Nat. Pharm. Prod..

[266] Papandreou M.A., Kanakis C.D., Polissiou M.G., Efthimiopoulos S., Cordopatis P., Margarity M., Lamari F.N. (2006). Inhibitory activity on amyloid-β aggregation and antioxidant properties of *Crocus sativus* stigmas extract and its crocin constituents. J. Agric. Food Chem..

[267] Termentzi A., Kokkalou E. (2008). LC-DAD-MS (ESI+) analysis and antioxidant capacity of *Crocus sativus* petal extracts. Planta Med..

[268] Hosseinzadeh H., Shamsaie F., Mehri S. (2009). Antioxidant activity of aqueous and ethanolic extracts of *Crocus sativus* L. stigma and its bioactive constituents, crocin and safranal. Pharmacogn. Mag..

[269] Acar G., Dogan N.M., Duru M.E., Kıvrak I. (2010). Phenolic profiles, antimicrobial and antioxidant activity of the various extracts of *Crocus* species in Anatolia. Afr. J. Microbiol. Res..

[270] Vatankhah E., Niknam V., Ebrahimzadeh H. (2010). Activity of antioxidant enzyme during *in vitro* organogenesis in *Crocus sativus*. Biol. Plant..

[271] Gismondi A., Serio M., Canuti L., Canini A. (2012). Biochemical, antioxidant and antineoplastic properties of Italian saffron (*Crocus sativus* L.). Am. J. Plant Sci..

[272] Sachdeva J., Tanwar V., Golechha M., Siddiqui K.M., Nag T.C., Ray R., Kumari S., Arya D.S. (2012). *Crocus sativus* L. (saffron) attenuates isoproterenol-induced myocardial injury via preserving cardiac functions and strengthening antioxidant defense system. Exp. Toxicol. Pathol..

[273] Rahaiee S., Moini S., Hashemi M., Shojaosadati S.A. (2015). Evaluation of antioxidant activities of bioactive compounds and various extracts obtained from saffron (*Crocus sativus* L.): A review. J. Food Sci. Tech..

[274] Wang C., Cai X., Hu W., Li Z., Kong F., Chen X., Wang D. (2019). Investigation of the neuroprotective effects of crocin via antioxidant activities in HT22 cells and in mice with Alzheimer’s disease. Internat. J. Mol. Med..

[275] Kim Y.K., Na K.S., Shin K.H., Jung H.Y., Choi S.H., Kim J.B. (2007). Cytokine imbalance in the pathophysiology of major depressive disorder. Prog. Neuro-Psychopharmacol. Biol. Psychiatry.

[276] Kim Y.K., Na K.S., Myint A.M., Leonard B.E. (2016). The role of pro-inflammatory cytokines in neuroinflammation, neurogenesis and the neuroendocrine system in major depression. Prog. Neuro-Psychopharmacol. Biol. Psychiatry.

[277] Liu T., Zhang L., Joo D., Sun S.C. (2017). NF-κB signaling in inflammation. Signal Transduct. Target Ther..

[278] Petralia M.C., Mazzon E., Fagone P., Basile M.S., Lenzo V., Quattropani M.C., di Nuovo S., Bendtzen K., Nicoletti F. (2020). The cytokine network in the pathogenesis of major depressive disorder. Close to translation?. Autoimmun. Rev..

[279] Nørbæk R., Kondo T. (1998). Anthocyanins from flowers of *Crocus* (Iridaceae). Phytochemistry.

[280] Nørbæk R., Kondo T. (1999). Further anthocyanins from flowers of *Crocus antalyensis* (Iridaceae). Phytochemistry.

[281] Castro-Díaz N., Salaun B., Perret R., Sierro S., Romero J.F., Fernández J.A., Rubio-Moraga A., Romero P. (2012). Saponins from the Spanish saffron *Crocus sativus* are efficient adjuvants for protein-based vaccines. Vaccine.

[282] Rubio-Moraga Á., Gerwig G.J., Castro-Díaz N., Jimeno M.L., Escribano J., Fernández J.A., Kamerling J.P. (2011). Triterpenoid saponins from corms of *Crocus sativus*: Localization, extraction and characterization. Ind. Crop. Prod..

[283] Esmaeili N., Ebrahimzadeh H., Abdi K., Safarian S. (2011). Determination of some phenolic compounds in *Crocus sativus* L. corms and its antioxidant activities study. Pharmacogn. Mag..

[284] Zhu Y., Han T., Hou T.T., Hu Y., Zhang Q.Y., Rahman K., Qin L.P. (2008). Comparative study of composition of essential oil from stigmas and of extract from corms of *Crocus sativus*. Chem. Nat. Compd..

[285] Ma S., Zhou S., Bing S. (1998). Pharmacological studies *Crocus* glycosides I. Effects on antiinflammatory and immune function. Chin. Tradit. Herbal Drugs.

[286] García-Olmo D.C., Riese H.H., Escribano J., Ontañón J., Fernandez J.A., Atiénzar M., García-Olmo D. (1999). Effects of long-term treatment of colon adenocarcinoma with crocin, a carotenoid from saffron (*Crocus sativus* L.): An experimental study in the rat. Nutr. Cancer..

[287] Arjmand M.H., Hashemzehi M., Soleimani A., Asgharzadeh F., Avan A., Mehraban S., Fakhraei M., Ferns G.A., Ryzhikov M., Gharib M. (2021). Therapeutic potential of active components of saffron in post-surgical adhesion band formation. J. Tradit. Complement. Med..

[288] Ma S., Liu B., Zhou S., Xu X., Yang Q., Zhou J. (1999). Pharmacological studies of glycosides of saffron crocus (*Crocus sativus*). II. Effects on blood coagulation, platelet aggregation and thromobosis. Chin. Tradit. Herbal Drugs.

[289] Liu T., Chu X., Wang H., Zhang X., Zhang Y., Guo H., Liu Z., Dong Y., Liu H., Liu Y. (2016). Crocin, a carotenoid component of *Crocus sativus*, exerts inhibitory effects on L-type Ca^2+^ current, Ca^2+^ transient, and contractility in rat ventricular myocytes. Can. J. Physiol. Pharmacol..

[290] Jaliani H.Z., Riazi G.H., Ghaffari S.M., Karima O., Rahmani A. (2013). The effect of the *Crocus sativus* L. carotenoid, crocin, on the polymerization of microtubules, in vitro. Iran. J. Basic. Med. Sci..

[291] Mancini A., Serrano-Díaz J., Nava E., D’alessandro A.M., Alonso G.L., Carmona M., Llorens S. (2014). Crocetin, a carotenoid derived from saffron (*Crocus sativus* L.), improves acetylcholine-induced vascular relaxation in hypertension. J. Vasc. Res..

[292] Siracusa L., Gresta F., Ruberto G., Yamaguchi M. (2011). Saffron (*Crocus sativus* L.) apocarotenoids: A review of their biomolecular features and biological activity perspectives. Carotenoids: Properties, Effects and Diseases.

[293] Hosseinzadeh H., Karimi G., Niapoor M. (2004). Antidepressant effect of *Crocus sativus* L. stigma extracts and their constituents, crocin and safranal, in mice. Acta Hort..

[294] Vahdati Hassani F., Naseri V., Razavi B.M., Mehri S., Abnous K., Hosseinzadeh H. (2014). Antidepressant effects of crocin and its effects on transcript and protein levels of CREB, BDNF, and VGF in rat hippocampus. DARU J. Pharm. Sci..

[295] Amin B., Nakhsaz A., Hosseinzadeh H. (2015). Evaluation of the antidepressant-like effects of acute and sub-acute administration of crocin and crocetin in mice. Avicenna J. Phytomed..

[296] Jam I.N., Sahebkar A.H., Eslami S., Mokhber N., Nosrati M., Khademi M., Foroutan-Tanha M., Ghayour-Mobarhan M., Hadizadeh F., Ferns G. (2017). The effects of crocin on the symptoms of depression in subjects with metabolic syndrome. Adv. Clin. Exp. Med..

[297] Sugiura M., Shoyama Y., Saito H., Nishiyama N. (1995). Crocin improves the ethanol-induced impairment of learning behaviors of mice in passive avoidance tasks. Proc. Jpn. Acad. B Phys. Biol. Sci..

[298] Hoshyar R., Bathaie S.Z., Ashrafi M. (2008). Interaction of safranal and picrocrocin with ctDNA and their preferential mechanisms of binding to GC- and AT-rich oligonucleotides. DNA Cell Biol..

[299] Song L., Kang C., Sun Y., Huang W., Liu W., Qian Z. (2016). Crocetin inhibits lipopolysaccharide-induced inflammatory response in human umbilical vein endothelial cells. Cell. Physiol. Biochem..

[300] Rameshrad M., Razavi B.M., Hosseinzadeh H. (2018). Saffron and its derivatives, crocin, crocetin and safranal: A patent review. Expert Opin. Ther. Pat..

[301] Wang W., He P., Zhao D., Ye L., Dai L., Zhang X., Sun Y., Zheng J., Bi C. (2019). Construction of *Escherichia coli* cell factories for crocin biosynthesis. Microb. Cell Factories.

[302] Acar B., Sadikoglu H., Ozkaymak M. (2011). Freeze drying of saffron (*Crocus sativus* L.). Dry Technol..

[303] Zarinkamar F., Tajik S., Soleimanpour S. (2011). Effects of altitude on anatomy and concentration of crocin, picrocrocin and safranal in *Crocus sativus* L.. Aust. J. Crop Sci..

[304] García-Rodríguez M.V., López-Córcoles H., Alonso G.L., Pappas C.S., Polissiou M.G., Tarantilis P.A. (2017). Comparative evaluation of an ISO 3632 method and an HPLC-DAD method for safranal quantity determination in saffron. Food Chem..

[305] Lage M., Cantrell C.L. (2009). Quantification of saffron (*Crocus sativus* L.) metabolites crocins, picrocrocin and safranal for quality determination of the spice grown under different environmental Moroccan conditions. Sci. Hortic..

[306] Azarabadi N., Özdemir F. (2018). Determination of crocin content and volatile components in different qualities of Iranian saffron. Gıda.

[307] Kyriakoudi A., Tsimidou M.Z., O’Callaghan Y.C., Galvin K., O’Brien N.M. (2013). Changes in total and individual crocetin esters upon *in vitro* gastrointestinal digestion of saffron aqueous extracts. J. Agric. Food Chem..

[308] Zhang A., Shen Y., Cen M., Hong X., Shao Q., Chen Y., Zheng B. (2019). Polysaccharide and crocin contents, and antioxidant activity of saffron from different origins. Ind. Crop. Prod..

[309] Zeka K., Ruparelia K.C., Continenza M.A., Stagos D., Vegliò F., Arroo R.R.J. (2015). Petals of *Crocus sativus* L. as a potential source of the antioxidants crocin and kaempferol. Fitoterapia.

[310] Moratalla-López N., Zalacain A., Bagur M.J., Salinas M.R., Alonso G.L., Morin J.F., Lees M. (2018). Saffron. Food Integrity Handbook: A Guide to Food Authenticity Issues and Analytical Solutions.

[311] Moshiri E., Basti A.A., Noorbala A.A., Jamshidi A.H., Hesameddin Abbasi S., Akhondzadeh S. (2006). *Crocus sativus* L. (petal) in the treatment of mild-to-moderate depression: A double-blind, randomized and placebo-controlled trial. Phytomedicine.

[312] Iborra J.L., Castellar M.R., Cánovas M., Manjón A. (1992). Picrocrocin hydrolysis by immobilized β-glucosidase. Biotechnol. Lett..

[313] Sánchez A.M., Carmona M., Jarén-Galán M., Mínguez Mosquera M.I., Alonso G.L. (2011). Picrocrocin kinetics in aqueous saffron spice extracts (*Crocus sativus* L.) upon thermal treatment. J. Agric. Food Chem..

[314] Alonso G.L., Salinas M.R., Garijo J., Sánchez-Fernández M.A. (2001). Composition of crocins and picrocrocin from Spanish saffron (*Crocus sativus* L.). J. Food Qual..

[315] del Campo C.P., Carmona M., Maggi L., Kanakis C.D., Anastasaki E.G., Tarantilis P.A., Polissiou M.G., Alonso G.L. (2010). Picrocrocin content and quality categories in different (345) worldwide samples of sffron (*Crocus sativus* L.). J. Agric. Food Chem..

[316] Othman R., Hatta F.A.M., Hassan N.M., Kamoona S. (2020). Characterization of carotenoids content and composition of saffron from different localities. J. Pharm. Nutr. Sci..

[317] Liakopoulou-Kyriakides M., Kyriakidi D. (2002). *Croscus sativus*-biological active constitutents. Stud. Nat. Prod. Chem..

[318] Mashmoul M., Azlan A., Khaza’ai H., Yusof B.N.M., Noor S.M. (2013). Saffron: A natural potent antioxidant as a promising anti-obesity drug. Antioxidants.

[319] Yeats T.H., Nagel R. (2018). Subcellular spice trade routes: Crocin biosynthesis in the saffron crocus (*Crocus sativus*). Plant Physiol..

[320] Bathaie S.Z., Mousavi S.Z. (2010). New applications and mechanisms of action of saffron and its important ingredients. Crit. Rev. Food Sci. Nutr..

[321] Hosseinzadeh H., Motamedshariaty V., Hadizadeh F. (2007). Antidepressant effect of kaempferol, a constituent of saffron (*Crocus sativus*) petal, in mice and rats. Pharmacologyonline.

[322] Mottaghipisheh J., Sourestani M.M., Kiss T., Horváth A., Tóth B., Ayanmanesh M., Ayanmanesh M., Khamushi A., Csupor D. (2020). Comprehensive chemotaxonomic analysis of saffron crocus tepal and stamen samples, as raw materials with potential antidepressant activity. J. Pharm. Biomed. Anal..

[323] Maggi L., Carmona M., Zalacain A., Kanakis C.D., Anastasaki E., Tarantilis P.A., Polissiou M.G., Alonso G.L. (2010). Changes in saffron volatile profile according to its storage time. Food Res. Int..

[324] Srivastava R., Ahmed H., Dixit R.K. (2010). *Crocus sativus* L.: A comprehensive review. Pharmacogn. Rev..

[325] Nyeem M.A.B., Alam M.K., Mizanur M., Khan R., Alam M.S., Ahammed M.M. (2018). Pharmacological effects of *Crocus sativus* (zaffran) and its chemical constituents: A review. Int. J. Physiol. Nutr. Phys. Educ..

[326] Feyzi S., Varidi M., Housaindokht M.R., Es’haghi Z. (2020). Innovative method for analysis of safranal under static and dynamic conditions through combination of HS-SPME-GC technique with mathematical modelling. Phytochem. Anal..

[327] Singla R.K., Bhat G.V. (2011). Crocin: An overview. Indo Glob. Res. J. Pharm. Sci..

[328] Shahi T., Assadpour E., Jafari S.M. (2016). Main chemical compounds and pharmacological activities of stigmas and tepals of ‘red gold’; saffron. Trends Food Sci. Technol..

[329] Rocchi R., Mascini M., Sergi M., Compagnone D., Mastrocola D., Pittia P. (2018). Crocins pattern in saffron detected by UHPLC-MS/MS as marker of quality, process and traceability. Food Chem..

[330] Hoshyar R., Mollaei H. (2017). A comprehensive review on anticancer mechanisms of the main carotenoid of saffron, crocin. J. Pharm. Pharmacol..

[331] Finley J.W., Gao S. (2017). A perspective on *Crocus sativus* L. (Saffron) constituent crocin: A potent water-soluble antioxidant and potential therapy for Alzheimer’s disease. J. Agric. Food Chem..

[332] Khazdair M.R., Boskabady M.H., Hosseini M., Rezaee R., Tsatsakis A.M. (2015). The effects of *Crocus sativus* (saffron) and its constituents on nervous system: A review. Avicenna J. Phytomed..

[333] Milajerdi A., Bitarafan V., Mahmoudi M. (2015). A review on the effects of saffron extract and its constituents on factors related to neurologic, cardiovascular and gastrointestinal diseases. J. Med. Plants.

[334] Farkhondeh T., Samarghandian S., Shaterzadeh Yazdi H., Samini F. (2018). The protective effects of crocin in the management of neurodegenerative diseases: A review. Am. J. Neurodegener. Dis..

[335] Sarris J. (2018). Herbal medicines in the treatment of psychiatric disorders: 10-year updated review. Phytother. Res..

[336] Bian Y., Zhao C., Lee S.M. (2020). Neuroprotective potency of saffron against neuropsychiatric diseases, neurodegenerative diseases, and other brain disorders: From bench to bedside. Front. Pharmacol..

[337] Ghajar A., Neishabouri S.M., Velayati N., Jahangard L., Matinnia N., Haghighi M., Ghaleiha A., Afarideh M., Salimi S., Meysamie A. (2017). *Crocus sativus* L. versus citalopram in the treatment of major depressive disorder with anxious distress: A double-blind, controlled clinical trial. Pharmacopsychiatry.

[338] Noorbala A.A., Akhondzadeh S., Tamacebi-Pour N., Jamshidi A.H. (2005). Hydro-alcoholic extract of *Crocus sativus* L. versus fluoxetine in the treatment of mild to moderate depression: A double-blind, randomized pilot trial. J. Ethnopharmacol..

[339] Akhondzadeh Basti A., Moshri E., Noorbala A.A., Jamshidi A.H., Abassi S.H., Akhondzadeh S. (2007). Comparison of petal of *Crocus sativus* L. and fluoxetine in the treatment of depressed outpatients: A pilot double-blind randomized trial. Prog. Neuro-Psychopharmocol. Biol. Psychiatry.

[340] Dai L., Chen L., Wang W. (2020). Safety and efficacy of saffron (*Crocus sativus* L.) for treating mild to moderate depression: A systematic review and meta-analysis. J. Nerv. Ment. Dis..

[341] Kashani L., Eslatmanesh S., Saedi N., Niroomand N., Ebrahimi M., Hosseinian M., Foroughifar T., Salimi S., Akhondzadeh S. (2017). Comparison of saffron versus fluoxetine in treatment of mild to moderate postpartum depression: A double-blind, randomized clinical trial. Pharmacopsychiatry.

[342] Khaksarian M., Behzadifar M., Behzadifar M., Alipour M., Jahanpanah F., Re T.S., Bragazzi N.L. (2019). The efficacy of *Crocus sativus* (Saffron) versus placebo and fluoxetine in treating depression: A systematic review and meta-analysis. Psychol. Res. Behav. Manag..

[343] Ahmadpanah M., Ramezanshams F., Ghaleiha A., Akhondzadeh S., Sadeghi Bahmani D., Brand S. (2019). *Crocus sativus* L. (saffron) versus sertraline on symptoms of depression among older people with major depressive disorders-a double-blind, randomized intervention study. Psychiatry Res..

[344] Dorri S.A., Hosseinzadeh H., Abnous K., Hasani F.V., Robati R.Y., Razavi B.M. (2015). Involvement of brain-derived neurotrophic factor (BDNF) on malathion induced depressive-like behavior in subacute exposure and protective effects of crocin. Iran. J. Basic Med. Sci..

[345] Basiri-Moghadam M., Hamzei A., Moslem A.R., Pasban-Noghabi S., Ghorbani N., Ghenaati J. (2016). Comparison of the anxiolytic effects of saffron (*Crocus sativus*. L) and diazepam before herniorrhaphy surgery: A double blind randomized clinical trial. Zahedan J. Res. Med. Sci..

[346] Sahraian A., Jelodar S., Javid Z., Mowla A., Ahmadzadeh L. (2016). Study the effects of saffron on depression and lipid profiles: A double blind comparative study. Asian J. Psychiatr..

[347] Hooshmandi Z., Rohani A.H., Eidi A., Fatahi Z., Golmanesh L., Sahraei H. (2011). Reduction of metabolic and behavioral signs of acute stress in male Wistar rats by saffron water extract and its constituent safranal. Pharm. Biol..

[348] Georgiadou G., Tarantilis P., Pitsikas N. (2012). Effects of the active constituents of *Crocus sativus* L., crocins, in an animal model of obsessive–compulsive disorder. Neurosci. Lett..

[349] Ettehadi H., Mojabi S., Ranjbaran M., Shams J., Sahraei H., Hedayati M., Asefi F. (2013). Aqueous extract of saffron (*Crocus sativus*) increases brain dopamine and glutamate concentrations in rats. J. Behav. Brain. Sci..

[350] Reddy H.S.G., Rajashekharappa R.S., Jayaram K.S., Jyothi C.H. (2013). Evaluation of antidepressant-like activity of *Crocus sativus* Linn stigmas in mice. Int. J. Pharm. Sci. Rev. Res..

[351] Sunanda B.P.V., Rammohan B., Kumar A. (2014). The effective study of aqueous extract of *Crocus sativus* Linn. (Saffron) in depressed mice. Int. J. PharmTech Res..

[352] Razavi B.M., Sadeghi M., Abnous K., Vahdati Hasani F., Hosseinzadeh H. (2017). Study of the role of CREB, BDNF, and VGF neuropeptide in long term antidepressant activity of crocin in the rat cerebellum. Iran. J. Pharm. Sci..

[353] Asrari N., Yazdian-Robati R., Abnous K., Razavi B.M., Rashednia M., Hasani F.V., Hosseinzadeh H. (2018). Antidepressant effects of aqueous extract of saffron and its effects on CREB, P-CREB, BDNF, and VGF proteins in rat cerebellum. J. Pharmacopunct..

[354] Ceremuga T.E., Ayala M.P., Chicoine C.R.W., Chun C.S.M., De Groot C.J.M., Henson D.T., Randall C.S.A., Stanley C.L.R., Beaumont C.D.M. (2018). Investigation of the anxiolytic and antidepressant effects of crocin, a compound from saffron (*Crocus sativus* L.), in the male sprague-dawley rat. AANA J..

[355] Farkhondeh T., Samarghandian S., Samini F., Sanati A.R. (2018). Protective effects of crocetin on depression-like behavior induced by immobilization in rat. CNS Neurol. Disord. Drug Targets.

[356] Zhang L., Previn R., Lu L., Liao R.F., Jin Y., Wang R.K. (2018). Crocin, a natural product attenuates lipopolysaccharide-induced anxiety and depressive-like behaviors through suppressing NF-kB and NLRP3 signaling pathway. Brain Res. Bull..

[357] Ghalandari-Shamami M., Nourizade S., Yousefi B., Vafaei A.A., Pakdel R., Rashidy-Pour A. (2019). Beneficial effects of physical activity and crocin against adolescent stress induced anxiety or depressive-like symptoms and dendritic morphology remodeling in prefrontal cortex in adult male rats. Neurochem. Res..

[358] Lu L., Wu D., Wang K., Tang J., Chen G. (2020). Beneficial effects of crocin against depression via pituitary adenylate cyclase-activating polypeptide. BioMed Res. Int..

[359] Xiao Q., Xiong Z., Yu C., Zhou J., Shen Q., Wang L., Xie X., Fu Z. (2019). Antidepressant activity of crocin-I is associated with amelioration of neuroinflammation and attenuates oxidative damage induced by corticosterone in mice. Physiol. Behav..

[360] Xie Y., He Q., Chen H., Lin Z., Xu Y., Yang C. (2019). Crocin ameliorates chronic obstructive pulmonary disease-induced depression via PI3K/Akt mediated suppression of inflammation. Eur. J. Pharmacol..

[361] Gao W., Wang W., Peng Y., Deng Z. (2019). Antidepressive effects of kaempferol mediated by reduction of oxidative stress, proinflammatory cytokines and up-regulation of AKT/β-catenin cascade. Metab. Brain Dis..

[362] Xiao Q., Shu R., Wu C., Tong Y., Xiong Z., Zhou J., Yu C., Xie X., Fu Z. (2020). Crocin-I alleviates the depression-like behaviors probably via modulating “microbiota-gut-brain” axis in mice exposed to chronic restraint stress. J. Affect. Disord..

[363] Tang J., Lu L., Wang Q., Liu H., Xue W., Zhou T., Xu L., Wang K., Wu D., Wei F. (2020). Crocin reverses depression-like behavior in Parkinson disease mice via VTA-mPFC pathway. Mol. Neurobiol..

[364] Orio L., Alen F., Ballesta A., Martin R., Gomez de Heras R. (2020). Antianhedonic and antidepressant effects of affron, a standardized saffron (*Crocus sativus* L.) extract. Molecules.

[365] Wu R., Xiao D., Shan X., Dong Y., Tao W.W. (2020). Rapid and prolonged antidepressant-like effect of crocin is associated with GHSR mediated hippocampal plasticity-related proteins in mice exposed to prenatal stress. Acs. Chem. Neurosci..

[366] Lin S., Li Q., Jiang S., Xu Z., Jiang Y., Liu L., Jiang J., Tong Y., Wang P. (2021). Crocetin ameliorates chronic restraint stress-induced depression-like behaviors in mice by regulating MEK/ERK pathways and gut microbiota. J. Ethnopharmacol..

[367] Kiashemshaki B., Safakhah H.A., Ghanbari A., Khaleghian A., Miladi-Gorji H. (2021). Saffron (*Crocus sativus* L.) stigma reduces symptoms of morphine-induced dependence and spontaneous withdrawal in rats. Am. J. Drug Alcohol Abuse..

[368] Monchaux De Oliveira C., Pourtau L., Vancassel S., Pouchieu C., Capuron L., Gaudout D., Castanon N. (2021). Saffron extract-induced improvement of depressive-like behavior in mice is associated with modulation of monoaminergic neurotransmission. Nutrients.

[369] Agha-Hosseini M., Kashani L., Aleyaseen A., Ghoreishi A., Rahmanpour H., Zarrinara A.R., Akhondzadeh S. (2008). *Crocus sativus* L. (saffron) in the treatment of premenstrual syndrome: A double-blind, randomised and placebo-controlled trial. BJOG Int. J. Obstet..

[370] Mansoori P., Akhondzadeh S., Raisi F., Ghaeli P., Jamshidi A.H., Nasehi A.A., Sohrabi H., Saroukhani S. (2011). A randomized, double blind, placebo—Controlled study of safety of the adjunctive saffron on sexual dysfunction induced by a selective serotonin reuptake inhibitor. J. Med. Plants.

[371] Moosavi S.M., Ahmadi M., Amini M., Vazirzadeh B. (2014). The effects of 40 and 80 mg hydro-alcoholic extract of *Crocus sativus* in the treatment of mild to moderate depression. J. Mazandaran. Univ. Med. Sci..

[372] Shahmansouri N., Farokhnia M., Abbasi S.H., Kassaian S.E., Noorbala Tafti A.A., Gougol A., Yekehtaz H., Forghani S., Mahmoodian M., Saroukhani S. (2014). A randomized, double-blind, clinical trial comparing the efficacy and safety of *Crocus sativus* L. with fluoxetine for improving mild to moderate depression in post percutaneous coronary intervention patients. J. Affect. Disord..

[373] Esalatmanesh S., Biuseh M., Noorbala A.A., Mostafavi S.A., Rezaei F., Mesgarpour B., Mohammadinejad P., Akhondzadeh S. (2017). Comparison of saffron and fluvoxamine in the treatment of mild to moderate obsessive-compulsive disorder: A double blind randomized clinical trial. Iran. J. Psychiatry.

[374] Modabbernia A., Sohrabi H., Nasehi A.A., Raisi F., Saroukhani S., Jamshidi A., Tabrizi M., Ashrafi M., Akhondzadeh S. (2012). Effect of saffron on fluoxetine-induced sexual impairment in men: Randomized double-blind placebo-controlled trial. Psychopharmacology.

[375] Kashani L., Raisi F., Saroukhani S., Sohrabi H., Modabbernia A., Nasehi A.A., Jamshidi A., Ashrafi M., Mansouri P., Ghaeli P. (2013). Saffron for treatment of fluoxetine-induced sexual dysfunction in women: Randomized double-blind placebo-controlled study. Hum. Psychopharmacol. Clin. Exp..

[376] Lopresti A.L., Drummond P.D. (2017). Efficacy of curcumin, and a saffron/curcumin combination for the treatment of major depression: A randomised, double-blind, placebo-controlled study. J. Affect. Disord..

[377] Abedimanesh N., Ostadrahimi A., Bathaie S.Z., Abedimanesh S., Motlagh B., Jafarabadi M.A., Sadeghi M.T. (2017). Effects of saffron aqueous extract and its main constituent, crocin, on health-related quality of life, depression, and sexual desire in coronary artery disease patients: A double-blind, placebo-controlled, randomized clinical trial. Iran. Red Crescent Med. J..

[378] Bangratz M., Ait Abdellah S., Berlin A., Blondeau C., Guilbot A., Dubourdeaux M., Lemoine P. (2018). A preliminary assessment of a combination of rhodiola and saffron in the management of mild-moderate depression. Neuropsychiatr. Dis. Treat..

[379] Jelodar G., Javid Z., Sahraian A., Jelodar S. (2018). Saffron improved depression and reduced homocysteine level in patients with major depression: A randomized, double-blind study. Avicenna J. Phytomed..

[380] Shakiba M., Moazen-Zadeh E., Noorbala A.A., Jafarinia M., Divsalar P., Kashani L., Shahmansouri N., Tafakhori A., Bayat H., Akhondzadeh S. (2018). Saffron (*Crocus sativus*) versus duloxetine for treatment of patients with fibromyalgia: A randomized double-blind clinical trial. Avicenna J. Phytomed..

[381] Kashani L., Esalatmanesh S., Eftekhari F., Salimi S., Foroughifar T., Etesam F., Safiaghdam H., Moazen-Zadeh E., Akhondzadeh S. (2018). Efficacy of *Crocus sativus* (saffron) in treatment of major depressive disorder associated with post-menopausal hot flashes: A double-blind, randomized, placebo-controlled trial. Arch. Gynecol. Obstet..

[382] Kell G., Rao A., Beccaria G., Clayton P., Inarejos-García A.M., Prodanov M. (2017). Saffron a novel saffron extract (*Crocus sativus* L.) improves mood in healthy adults over 4 weeks in a double-blind, parallel, randomized, placebo-controlled clinical trial. Complement. Ther. Med..

[383] Lopresti A.L., Smith S.J., Hood S.D., Drummond P.D. (2019). Efficacy of a standardised saffron extract saffron as an add-on to antidepressant medication for the treatment of persistent depressive symptoms in adults: A randomised, double-blind, placebo-controlled study. J. Psychopharmacol..

[384] Ghaderi A. (2019). Clinical and metabolic responses to crocin in patients under methadone maintenance treatment, a randomized clinical trial. Phytother. Res..

[385] Lopresti A.L., Smith S.J. (2021). The effects of a saffron extract saffron on menopausal symptoms in women during perimenopause: A randomised, double-blind, placebo-controlled study. J. Menopausal. Med..

[386] Jalali F., Hashemi S. (2018). The effect of saffron on depression among recovered consumers of methamphetamine living with HIV/AIDS. Subst. Use Misuse..

[387] Milajerdi A., Jazayeri S., Shirzadi E., Hashemzadeh N., Azizgol A., Djazayery A., Esmaillzadeh A., Akhondzadeh S. (2018). The effects of alcoholic extract of saffron (*Crocus satious* L.) on mild to moderate comorbid depression-anxiety, sleep quality, and life satisfaction in type 2 diabetes mellitus: A double-blind, randomized and placebo-controlled clinical trial. Complement. Ther. Med..

[388] Moazen-Zadeh E., Abbasi S.H., Safi-Aghdam H., Shahmansouri N., Arjmandi-Beglar A., Hajhosseinn Talasaz A., Salehiomran A., Forghani S., Akhondzadeh S. (2018). Effects of saffron on cognition, anxiety, and depression in patients undergoing coronary artery bypass grafting: A randomized double-blind placebo-controlled trial. J. Altern. Complement. Med..

[389] Khalatbari-Mohseni A., Banafshe H.R., Mirhosseini N., Asemi Z., Ghaderi A., Omidi A. (2019). The effects of crocin on psychological parameters in patients under methadone maintenance treatment: A randomized clinical trial. Subst. Abuse Treat. Prev. Policy.

[390] Akhondzadeh S., Mostafavi S.A., Keshavarz S.A., Mohammadi M.R., Hosseini S., Eshraghian M.R. (2020). A placebo controlled randomized clinical trial of *Crocus sativus* L. (saffron) on depression and food craving among overweight women with mild to moderate depression. J. Clin. Pharm. Ther..

[391] Nemat-Shahi M., Asadi A., Nemat-Shahi M., Soroosh D., Mozari S., Bahrami-Taghanaki H., Mehrpour M. (2020). Comparison of saffron versus fluoxetine in treatment of women with premenstrual syndrome: A randomized clinical trial study. Indian J. Forensic Med. Toxicol..

[392] Jackson P.A., Forster J., Khan J., Pouchieu C., Dubreuil S., Gaudout D., Moras B., Pourtau L., Joffre F., Vaysse C. (2021). Effects of saffron extract supplementation on mood, well-being, and response to a psychosocial stressor in healthy adults: A randomized, double-blind, parallel group, clinical trial. Front. Nutr..

[393] Moghadam B.H., Bagheri R., Roozbeh B., Ashtary-Larky D., Gaeini A.A., Dutheil F., Wong A. (2021). Impact of saffron (*Crocus Sativus* Linn) supplementation and resistance training on markers implicated in depression and happiness levels in untrained young males. Physiol. Behav..

[394] Salek R., Dehghani M., Mohajeri S.A., Talaei A., Fanipakdel A., Javadinia S.A. (2021). Amelioration of anxiety, depression, and chemotherapy-related toxicity after crocin administration during chemotherapy of breast cancer: A double-blind, randomized clinical trial. Phytother. Res..

[395] Tajaddini A., Roshanravan N., Mobasseri M., Aeinehchi A., Sefid-Mooye Azar P., Hadi A., Ostadrahimi A. (2021). Saffron improves life and sleep quality, glycaemic status, lipid profile and liver function in diabetic patients: A double-blind, placebo-controlled, randomised clinical trial. Int. J. Clin. Pract..

[396] Jomehpour H., Aghayan S., Khosravi A., Afzaljavan F. (2022). The effect of krocina on decreasing substance user withdrawal syndrome, craving, depression and stress: A double-blind randomized parallel clinical trial. Subst. Use Misuse.

[397] Bashir H., Kumhar S.H., Qadir W. (2021). Effect of *Crocus sativus* (saffron) on corona virus. World J. Pharm. Pharm. Sci..

[398] Husaini A.M., Jan K.N., Wani G.A. (2021). Saffron: A potential drug-supplement for severe acute respiratory syndrome coronavirus (COVID) management. Heliyon.

[399] Mentis A.F.A., Dalamaga M., Lu C., Polissiou M.G. (2021). Saffron for “toning down” COVID-19-related cytokine storm: Hype or hope? A mini-review of current evidence. Metab. Open.

[400] Soheilipur K., Khazdair M.R., Moezi S.A., Mahmoudirad G. (2021). Comparing the effects of saffron, lippia, and saffron-lippia combination on anxiety among candidates for coronary angiography. Avicenna J. Phytomed..

[401] Shahdadi H., Balouchi A., Dehghanmehr S. (2017). Effect of saffron oral capsule on anxiety and quality of sleep of diabetic patients in a tertiary healthcare facility in southeastern Iran: A quasi-experimental study. Trop. J. Pharm. Res..

[402] Pachikian B.D., Copine S., Suchareau M., Deldicque L. (2021). Effects of saffron extract on sleep quality: A randomized double-blind controlled clinical trial. Nutrients.

[403] Lopresti A.L., Smith S.J., Metse A.P., Drummond P.D. (2020). Effects of saffron on sleep quality in healthy adults with self-reported poor sleep: A randomized, double-blind, placebo-controlled trial. J. Clin. Sleep. Med..

[404] Lopresti A.L., Smith S.J., Drummond P.D. (2021). An investigation into an evening intake of a saffron extract (Saffron^®^) on sleep quality, cortisol, and melatonin concentrations in adults with poor sleep: A randomised, double-blind, placebo-controlled, multi-dose study. Sleep Med..

[405] Umigai N., Takeda R., Mori A. (2018). Effect of crocetin on quality of sleep: A randomized, double-blind, placebo-controlled, crossover study. Complement. Ther. Clin. Pract..

[406] Fukui H., Toyoshima K., Komaki R. (2011). Psychological and neuroendocrinological effects of odor of saffron (*Crocus sativus*). Phytomedicine.

[407] Lechtenberg M., Schepmann D., Niehues M., Hellenbrand N., Wünsch B., Hensel A. (2008). Quality and functionality of saffron: Quality control, species assortment and affinity of extract and isolated saffron compounds to NMDA and sigma1 (sigma-1) receptors. Planta Med..

[408] Abe K., Sugiura M., Shoyama Y., Saito H. (1998). Crocin antagonizes ethanol inhibition of NMDA receptor-mediated responses in rat hippocampal neurons. Brain Res..

[409] Hausenblas H.A., Saha D., Dubyak P.J., Anton S.D. (2013). Saffron (*Crocus sativus* L.) and major depressive disorder: A meta-analysis of randomized clinical trials. J. Integr. Med..

[410] Dovrtělová G., Nosková K., Juřica J., Turjap M., Zendulka O. (2015). Can bioactive compounds of *Crocus sativus* L. influence the metabolic activity of selected CYP enzymes in the rat?. Physiol. Res..

[411] Dahchour A. (2021). The effects of saffron (*Crocus sativus* L.) and its components on depression: From basic to clinical studies. Moroccan J. Biol..

[412] Siddiqui S.A., Ali Redha A., Snoeck E.R., Singh S., Simal-Gandara J., Ibrahim S.A., Jafari S.M. (2022). Anti-depressant properties of crocin molecules in saffron. Molecules.

[413] Perlis R.H. (2007). Cytochrome P450 genotyping and antidepressants. Br. Med. J..

[414] Dubovsky S.L. (2015). The usefulness of genotyping cytochrome P450 enzymes in the treatment of depression. Expert. Opin. Drug. Metab. Toxicol..

